# Transformations, Lineage Comparisons, and Analysis of Down-to-Up Protomer States of Variants of the SARS-CoV-2 Prefusion Spike Protein, Including the UK Variant B.1.1.7

**DOI:** 10.1128/spectrum.00030-21

**Published:** 2021-08-04

**Authors:** Michael H. Peters, Oscar Bastidas, Daniel S. Kokron, Christopher E. Henze

**Affiliations:** a Department of Chemical and Life Science Engineering, Virginia Commonwealth University, Richmond, Virginia, USA; b College of Biological Sciences, University of Minnesota, Minneapolis, Minnesota, USA; c NASA Ames Research Center, Moffett Field, California, USA; University of Texas at San Antonio

**Keywords:** COVID-19, prefusion, SARS-CoV-2, Spike protein, variants, betacoronaviruses

## Abstract

Monitoring and strategic response to variants in severe acute respiratory syndrome coronavirus 2 (SARS-CoV-2) represent a considerable challenge in the current pandemic and for future viral outbreaks. Mutations/deletions of the virion’s prefusion Spike protein may have significant impact on vaccines and therapeutics that utilize this key structural protein in their mitigation strategies. In this study, we have demonstrated how dominant energetic landscape mappings (“glue points”) based on *ab inito* all-atom force fields coupled with phylogenetic sequence alignment information can identify key residue mutations and deletions associated with variants. We also found several examples of excellent homology of stabilizing residue glue points across the lineages of betacoronavirus Spike proteins that we have called “sequence homologous glue points.” SARS-CoV-2 demonstrates the least number of stabilizing glue points associated with interchain interactions among Down-state protomers across lineages. Additionally, we computationally studied variants among the trimeric Spike protein of SARS-CoV-2 using all-atom molecular dynamics to ascertain structural and energetic changes among variants. We examined both a theoretically based triple mutant and the UK or B.1.1.7 variant. For the theoretical triple mutant, we demonstrated through alanine substitutions that three key residues could cause the transition of Down-to-Up protomer states, where the transition is characterized by the “arm” length of the receptor-binding domain (RBD) rather than the hinge angle. For the B.1.1.7 variant, we demonstrated the critical importance of mutations D614G and N501Y on the structure and binding, respectively, of the Spike protein. We note that these same two key mutations are also found in the South African B.1.351 variant.

**IMPORTANCE** Viral variants represent a major challenge to monitoring viral outbreaks and formulating strategic health care responses. Variants represent transmitting viruses that have specific mutations and deletions associated with their genome. In the case of SARS-CoV-2 and other related viruses (betacoronaviruses), many of these mutations and deletions are associated with the Spike protein that the virus uses to infect cells. Here, we have analyzed both SARS-CoV-2 variants and related viruses, such as Middle Eastern respiratory syndrome coronavirus (MERS-CoV), in order to understand not only differences, but also key similarities between them. Understanding similarities can be as important as differences in determining key functional features of a class of viruses, such as the betacoronaviruses. We have used both phylogenetic analysis, which traces genetic similarities and differences, along with independent biophysics analysis, which adds function or behavior, in order to determine possible functional differences and hence possible transmission and infection differences among variants and lineages.

## INTRODUCTION

Betacoronaviruses represent one (B) of four genera (A, B, C, and D) of RNA positive-sense viruses in the *Nidovirales* order (1, 2). The current pandemic COVID-19, caused by SARS-CoV-2, is the latest in human viral outbreaks of this genera, preceded by the Middle Eastern respiratory syndrome coronavirus (MERS-CoV) and the SARS-CoV outbreak of 2002. In the current pandemic, SARS-CoV-2 continues to exhibit high rates of transmission and infection across the globe. Of great present concern are variants that may show relative increased transmission and infection rates and, in addition, may present challenges to current and developing vaccines, as well as therapeutics aimed at mitigation of this deadly virion. Of note is that this virus has a genome size of ∼30 kb and an intrinsic proofreading mechanism to reduce mutation rates (3). The mutation rate of SARS-CoV-2 has been estimated to be ∼10^– 3^ substitutions per site per year (3). Genomic sequences of SARS-CoV-2 continue to be deposited in the GSAID (Global Initiative on Sharing all Influenza Data), which has allowed for the study of structural implications of mutations (4). For example, the Spike protein mutation D614G has been associated with higher upper respiratory tract viral loads and appears to be omnipresent in recent genomic sequences across the globe (4–6). In addition, another variant called the UK variant or VOC 202012/01 or B.1.1.7 (classification system [7]) has been identified as a highly transmittable variant and involves both deletions and mutations in the Spike protein of this virion, including D614G. Thus, it is of great importance to determine how current and future variants may translate into altered transmission rates, viral loading differences, antibody and vaccine escape, and resistance to currently developing therapeutics. Here, we focus on the analysis of mutations of the prefusion Spike protein due to its importance to vaccines and therapeutics, as a partial guide to the potential effects of its mutations on structure, function, and possible behavioral changes of this virion.

**FIG 1:**
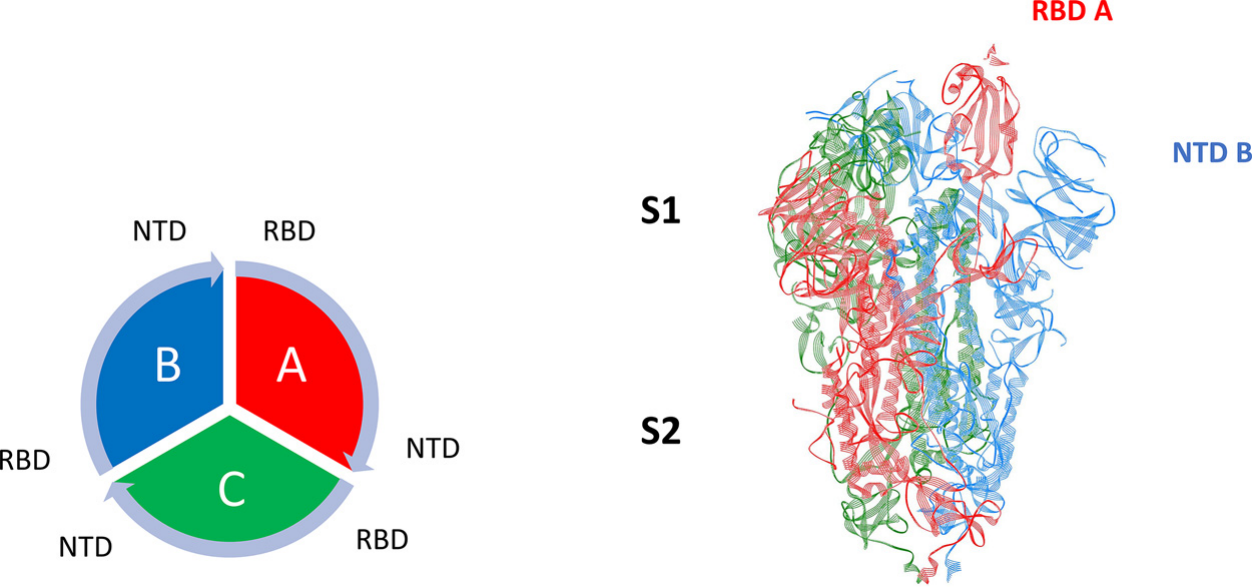
SARS-Cov-2 betacoronavirus (PDB ID: 6VSB) with one Up (A) and two Down (B and C) showing the S1 (binding ectodomain) and S2 (fusion) domains. Also shown is the overall chain interaction configuration looking at the trimer from the top view.

A distinct characteristic of the coronaviruses are their large, trimeric Spike proteins that densely decorate the virion surface (8–10). The Spike protein consists of three homologous protomers, or chains, where each one is ∼1,200 amino acid residues in length (Fig. 1). In its prefusion state, each protomer consists of two large domains called S1 (most distal from the virion membrane) and S2 (most proximal to the membrane). In general, the S1 domain represents a prefusion domain (∼650 residues) and the S2 domain (∼600 residues) is the fusion domain. The S2 or fusion machinery domain is relatively rigid with strong noncovalent intra- and interchain interactions facilitated by helical secondary structures, whereas the S1 domain, which contains the host cell receptor binding domain (RBD) and N-terminal domain (NTD) in a V-shaped configuration (Fig. 1), is weaker, flexible, and characterized by beta-strand secondary structural motifs (11). We note that the S1 domain of the Spike protein is noncovalently bound to the S2 domain in the native state via a furin cleavage site (10). The configuration of the RBD in the prefusion state is further characterized as being in the so-called “Up-state” or “Down-state,” depending on the position of the RBD relative to the center of mass of the prefusion Spike protein. For example, in the Up-state, the RBD of both SARS-CoV and SARS-CoV-2 is more exposed and able to bind to its ACE2 (angiotensin converting enzyme 2) receptor on the surface of human epithelial cells (type I and II pneumocytes; also, alveolar macrophage and nasal mucosal cells), whereas in the “Down-state,” the RBD is believed to be more hidden and significantly reduced to ACE2 binding and to cellular infection (12–14). Quantified structural comparisons of the RBD configuration in the Up versus Down protomer states of SARS-CoV-2 have recently been done that include angular positions of the RBD relative to the NTD of a given protomer (8). Henderson et al. (13) also quantified angular differences in the S1 subdomains of the Spike protein across the betacoronaviruses SARS-CoV-2, SARS-CoV, MERS-CoV, and human coronavirus HKU1 and developed mutational forms that can alter the equilibrium of Up versus Down states.

Given the critical importance of emerging variants of SARS-CoV-2 to vaccines and therapeutics, it is important to analyze the effects of mutations and/or deletions on the stability and dynamics of the Spike protein. Previously, we studied the stability and dynamics of the entire Spike protein of SARS-CoV-2 using a combination of all-atom dominant energetic analyses and biophysical computational molecular dynamics using published structures of the trimeric Spike protein (11). We determined energetically dominant, noncovalent intraprotomer and interprotomer interactions, “called” “glue points” or “hot spots” that help stabilize the entire trimeric protein structure. For example, we previously identified D614 as a key glue point with neighboring protomer residues (11, 15) (Table S1 in the supplemental material) prior to its mutational emergence (as D614G) in current variants. We also mutated a key hot spot (“latch” residues) associated with intraprotomer interactions in order to demonstrate the ability for single protomers to change from Down to Up states. However, it was further demonstrated that in complete trimeric structures, such transitions are held in check by interprotomer interactions, specifically, the RBD of any protomer in its Down state with the NTD of its neighboring protomer.

In the current study, we computationally analyze structure and dynamics of key mutations associated with the trimeric SARS-CoV-2 prefusion Spike protein and their role in helping to maintain Down states and potentially less transmission; in particular, we examine a theoretically based triple mutant, based on our glue point mappings, that is shown to destabilize neighboring RDB-NTD interactions and lead to a transition of Down-to-Up protomer states. We are also interested in how these stabilizing RBD-NTD interactions may be conserved across the lineages of the betacoronaviruses SARS-CoV, MERS-CoV, and HCoV, since such comparisons may be useful in identifying critical deletions/mutations across current and future variants of SARS-CoV-2 and possibly future emerging betacoronaviruses. We demonstrate through sequence alignment and energetic mappings highly conserved stabilizing glue point residues across the lineages of betacoronaviruses. In addition, using these tools, we critically examine the UK variant B.1.1.7, including the D614G mutation, in order to discern key differences in protomer configurations that could potentially impact vaccine and therapeutic efforts aimed at the debilitation of the current pandemic. We then dynamically analyze any associated conformational changes using all-atom molecular dynamics of the trimeric prefusion spike protein of B.1.1.7 over a 0.5 microsecond time period in order to ascertain key differences in behavior from the wild type. We are able to demonstrate dynamic changes in the B.1.1.7 spike protein that can be traced to two key mutations, resulting in a more accessible RBD (D614G) and simultaneously stronger binding to ACE2 (N501Y). These key mutations are also present in the rapidly emerging South African variant B.1.351. Our findings and analysis may have general applicability and may be important at ascertaining the potential effects of future variations of this virion on vaccine and therapeutics, as well as possible relation to infection and transmission rates, in an attempt to stay ahead via structure-to-function analysis of emerging variants. We note that our analysis here is focused on the prefusion state of the Spike protein and does not consider the additional steps of fusion, uptake, and virion replication in host cells, which are all important to the overall transmission and infection process.

## RESULTS

### Sequence alignment and glue point residues across lineages of betacoronaviruses.

**FIG 2:**
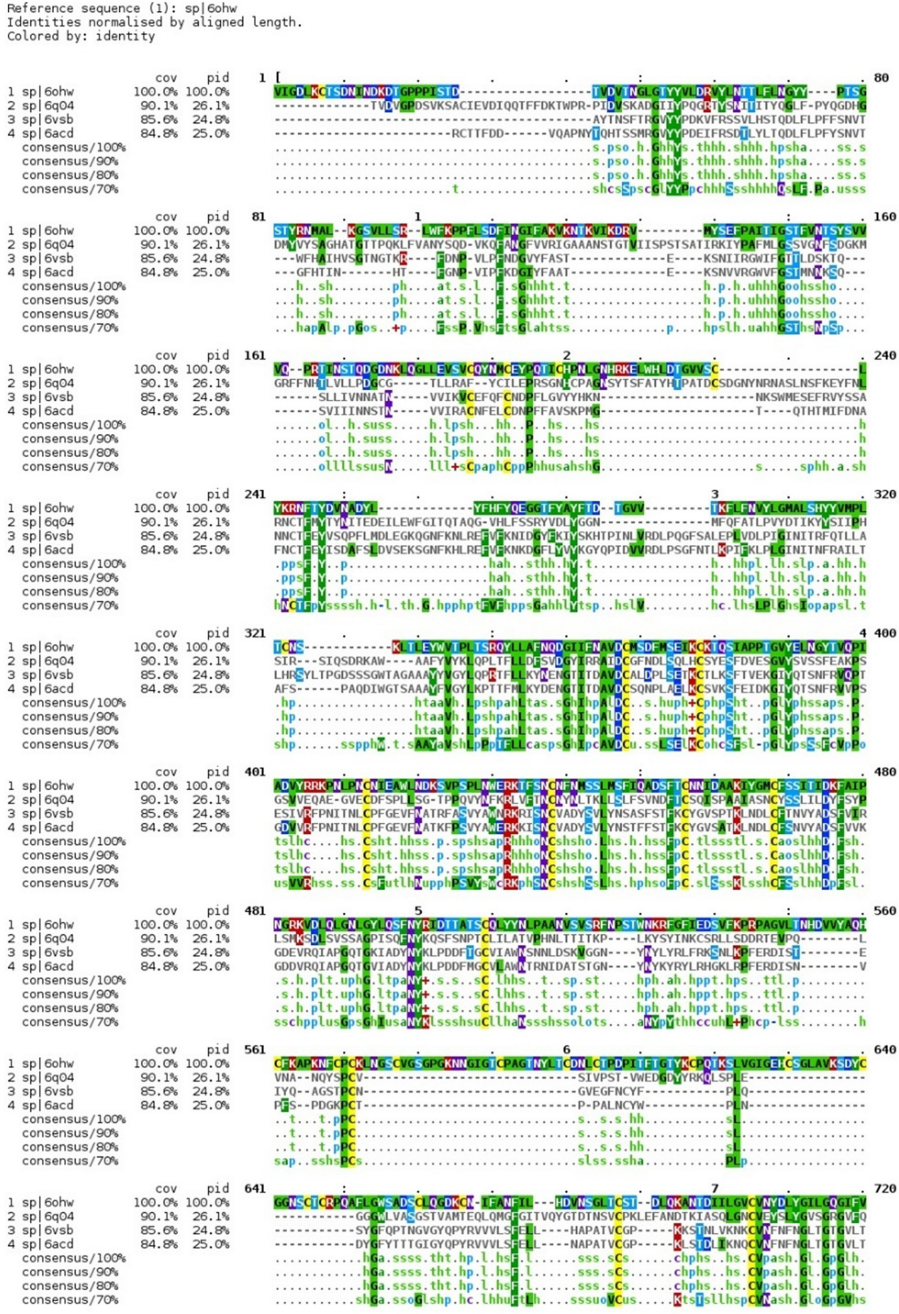
Sequence alignment for SARS-Cov, SARS-CoV-2, MERS-CoV, and HCoV. The original pdf file is included in the supplemental material for ease of viewing.

**FIG 3:**
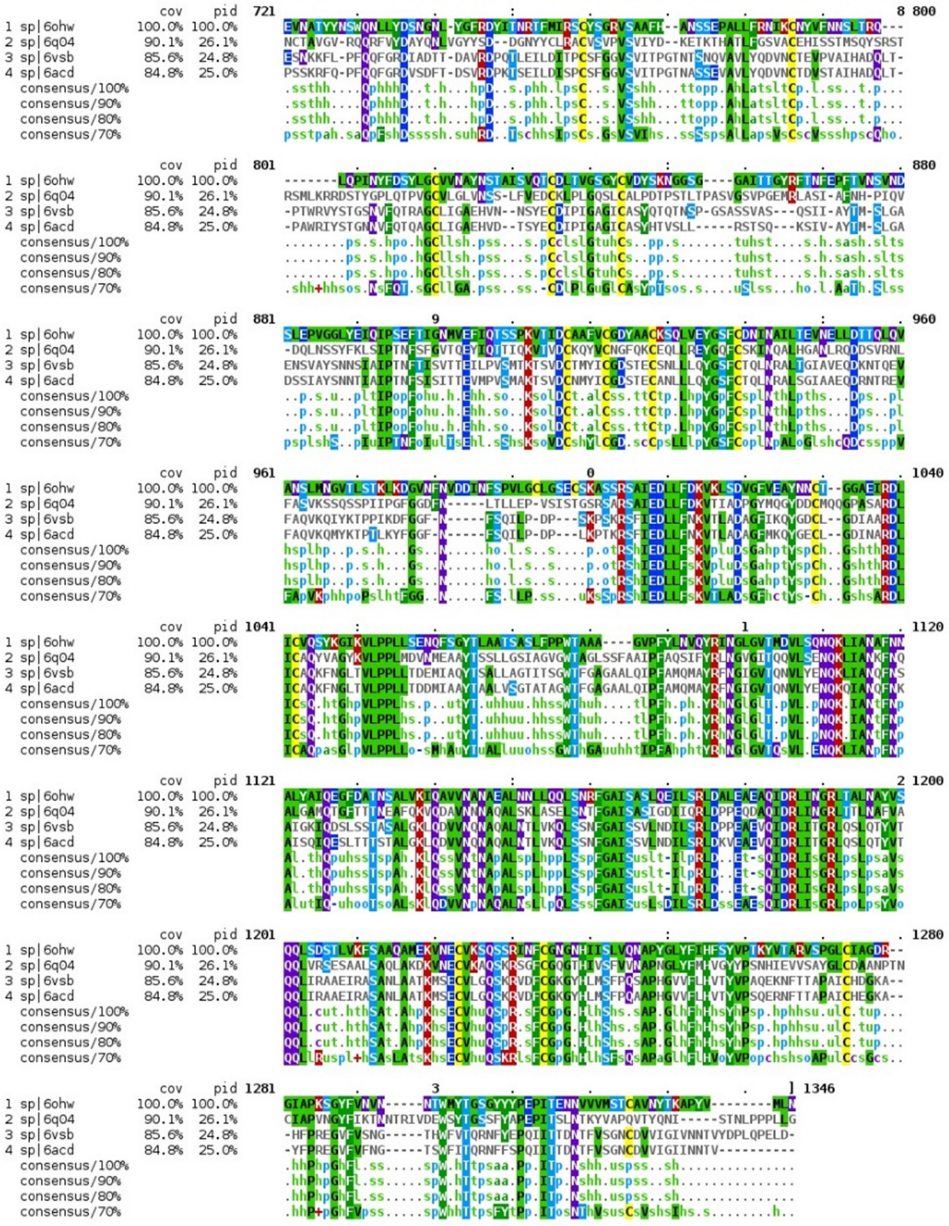
Sequence alignment for SARS-Cov, SARS-CoV-2, MERS-CoV, and HCoV. The original pdf file is included in the supplemental material for ease of viewing.

**FIG 4:**
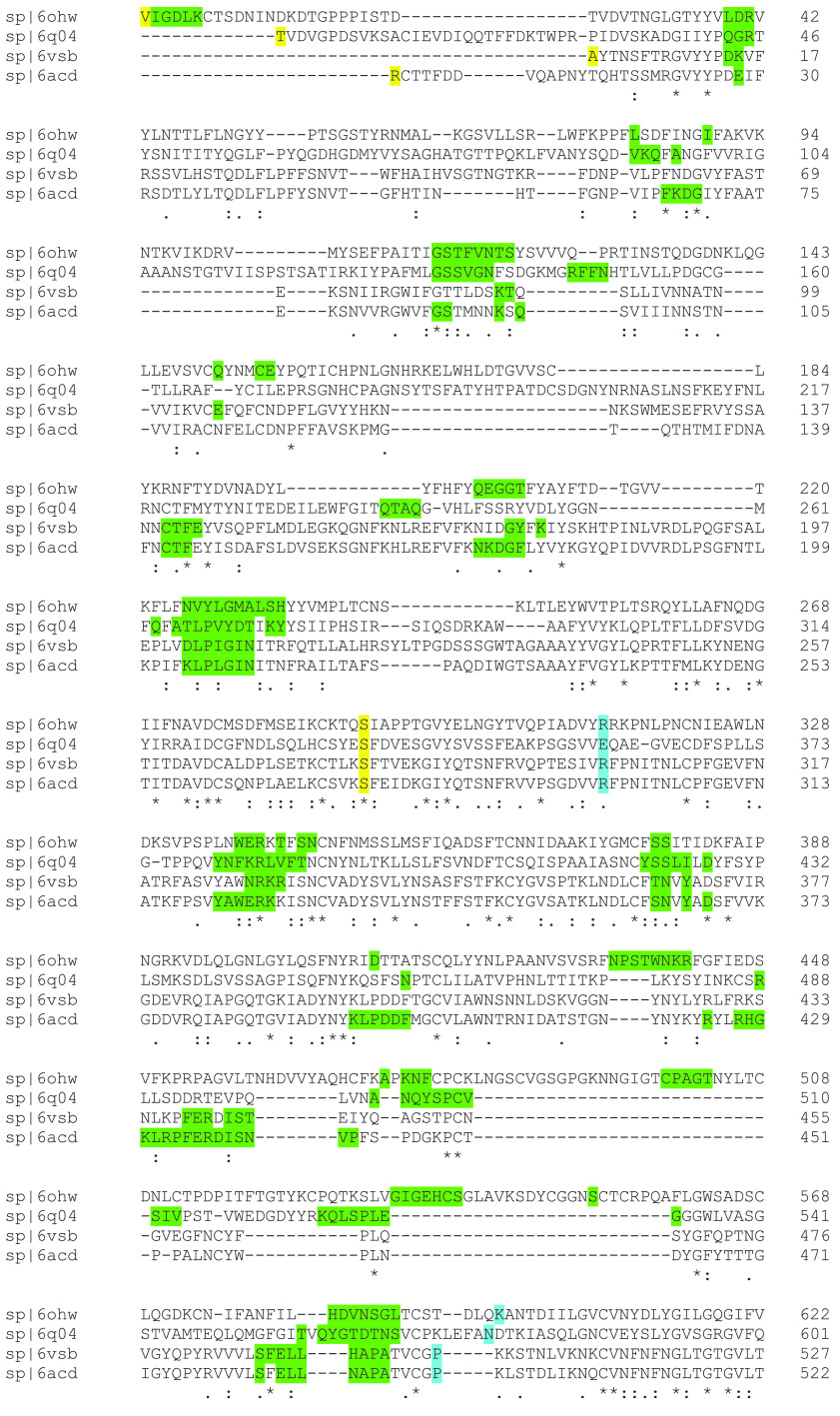
Combination of the sequence alignment map with the glue point map for the S1 domain across the betacoronaviruses. Green shaded letters are the residues associated with dominant energetic interactions or glue points between the RBD and its neighboring NTD both in the Down state. Yellow shaded letters mark the start and end of the NTD and blue shaded letters mark the beginning and end of the RBD across these lineages.

The color map sequence alignment across the entire Spike protein for the four betacoronaviruses, as obtained from Clustal Omega, is shown in Fig. 2 and 3. As can be seen, the greatest overall alignment homology is with the S2 or fusion domain and S1-NTD of this protein, and the greatest variation is in the RBD of S1. Next, the dominant energetic contacts or glue points of the stabilizing RBD-NTD neighboring Down-Down chain interactions across these lineages were determined and mapped over the sequence alignment, as shown in Fig. 4. Somewhat surprisingly, we found excellent sequence homology across many of the glue points despite clade differences among these lineages; below we refer to these as “sequence homologous glue points.” For example, 6OHW (residues 225 to 231), 6Q04 (residues 266 to 273), 6VSB (residues 202 to 209), and 6ACD (residues 204 to 211) demonstrate an exact alignment of their glue points. It is also interesting to note that SARS-CoV and SARS-CoV-2 have lost stabilizing RBD-NTD interactions compared to both MERS-CoV and HCoV in their acquired ACE2 receptor binding domain, as discussed more fully below. It is important to point out that the glue point analysis is completely independent of the phylogenetic sequence alignment and is based on *ab initio* force field models among the atoms of the residue-residue interactions. Thus, the combination analysis of phylogenetics and, independently, *ab initio* biophysics may be quite illuminating in general to structure-function relationships within structural viral proteins.

**FIG 5:**
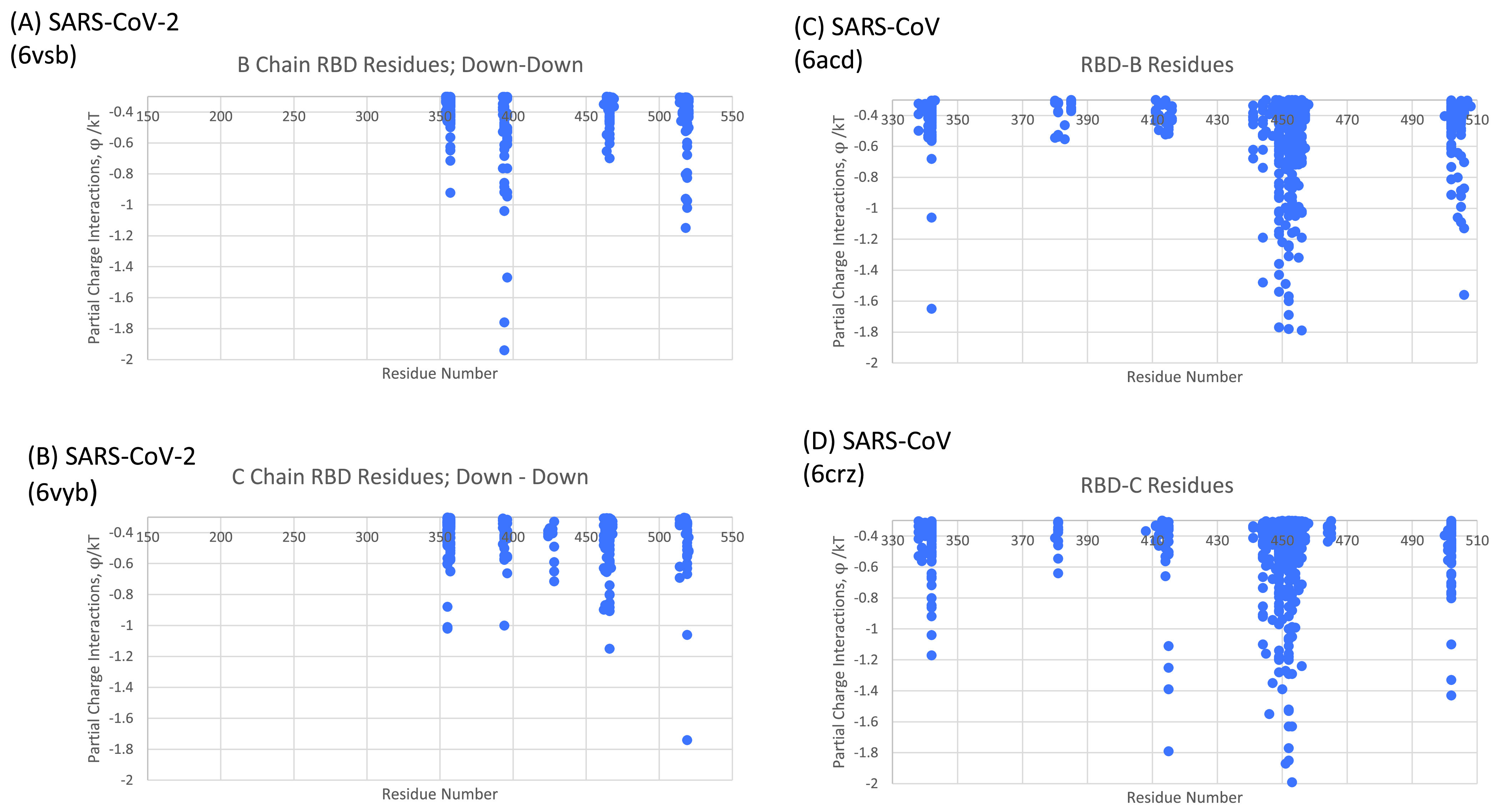
Comparison of SARS-CoV (C and D) and SARS-CoV-2 (A and B) RBD-NTD neighboring chain glue points. Note that all protomers are in the Down state here. See reference 11 and the supplemental material for (A) and (B). Data for (C) are in Table S1 and data for (D) are in Table S2.

Additionally, by comparing just the SARS-CoV and SARS-CoV-2 structures, as shown in Fig. 5, we found significantly larger numbers of atom-atom interactions for the same glue point residues associated with SARS-CoV, indicative of a much stronger and more stable Down-Down state configuration for SARS-CoV. Interestingly, SARS-CoV-2 demonstrates the least total number of glue points and potentially diminished stability across these lineages. Note that the results in Fig. 5 are based on multiple, independently obtained experimental PDB-deposited structure files: SARS-CoV-2: 6VSB (9) and 6VYB (10); SARS-CoV: 6ACD (14) and 6CRZ (16).

### Triple mutant versus wild type.

**FIG 6:**
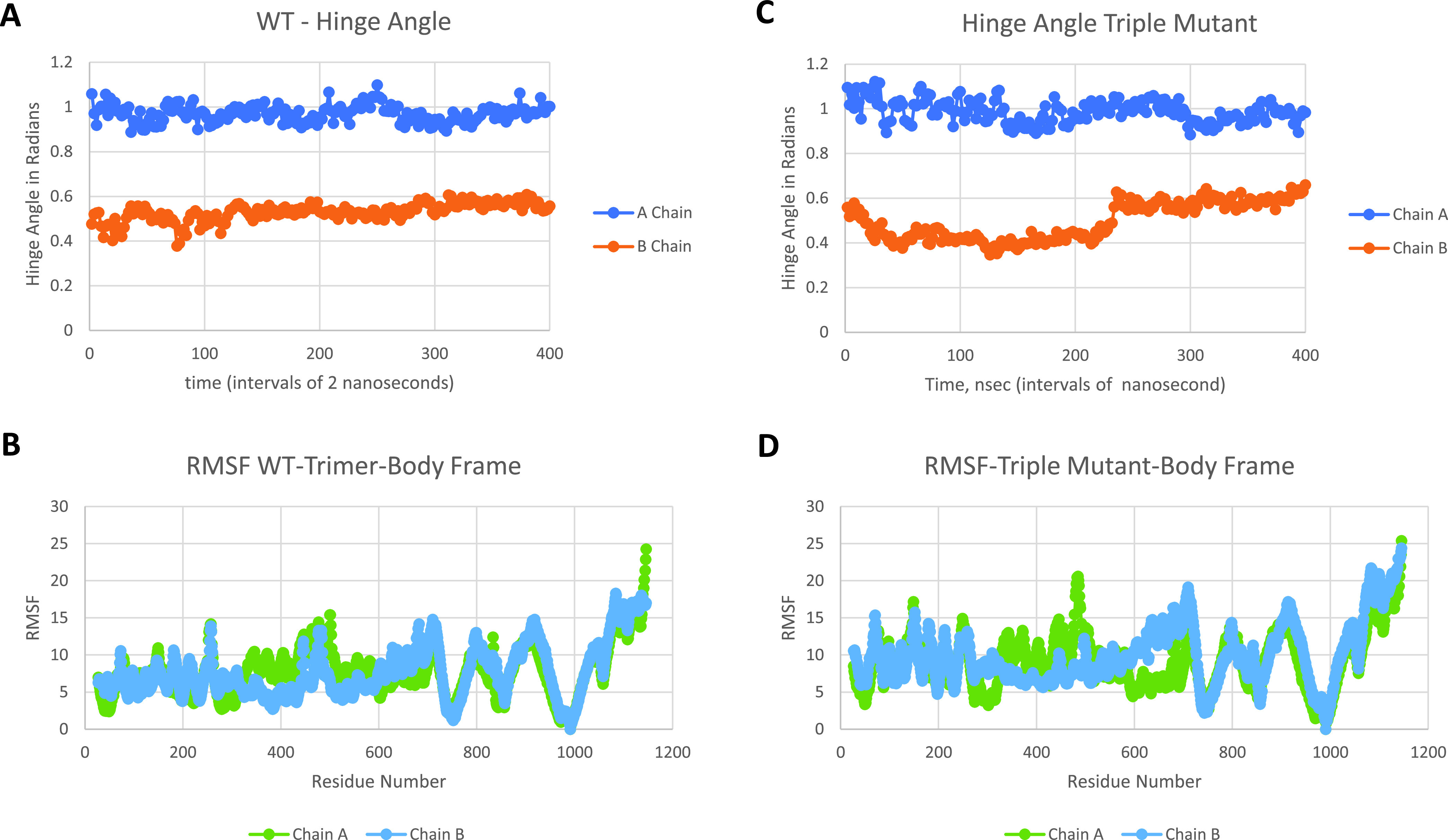
Hinge angle and RMSF values for wild-type (A and B) frames and triple mutant (C and D) frames, respectively; chain A is Up and chain B is Down. RMSF values are shown for the last 200 nsec of the simulation.

Previously, we identified three critical glue point residues that help stabilize RBD-NTD interprotomer interactions across both Down-Down and Up-Down states of SARS-CoV-2, *viz*, ARG357, ASN394, and HIS519 (11) (Fig. 3 and 4). These interactions helped prevent “latch” release from Down-to-Up states associated with Down-state intraprotomer latch residues: GLN564 to ALA520-PRO521-ALA522. It is further interesting to note from Fig. 4 that these three stabilizing residues are also part of the sequence homologous glue points across the lineages of betacoronaviruses. Here, we examine the triple alanine mutant ARG357ALA, ASN394ALA, and HIS519ALA across the entire SARS-CoV-2 Spike protein (mutating 6VSB, see Materials and Methods) in order to determine if these key glue points alone could cause a conformational change in the absence of any latch mutations. Note that this so-called “alanine screening” should diminish side chain interactions of those residues (i.e., “unglue”) without significant initial structure changes. Figure 6A to D shows molecular dynamic (MD) calculated values of the hinge angle and root mean square fluctuation (RMSF) values for what we have called wild-type (WT) SARS-CoV-2 and the theoretical triple mutant. It is clear that a longer time period of 150 nsec is required to reach a dynamic equilibrium state of either of these proteins from the starting configurations that include an initial constant number-volume-temperature equilibrium period. For fairness, we determined the RMSF values after the first 150 nsec of simulation. The WT hinge angle for the Up-state protomer (A chain) equilibrates at 1 rad or approximately 60 deg, putting it on the lower end of the ACE2-accessible region according to the criteria of Peng et al. (17). Additionally, the theoretical triple mutant Down B chain is also shown to equilibrate at around 0.6 rad, keeping it in the ACE-2 inaccessible region, according to the referenced definition, after approximately 220 nsec (Fig. 6C) and continues there for up to 720 nsec (Data Set S1). However, it was clear from visualization programs that the Down-state protomer “arm” was lengthening to an Up-state value, and the arm lengths were quantified as will be shown below. Thus, the hinge angle as defined may not be the single best criteria of Up versus Down protomers. The triple mutant also shows more flexibility in its S1 domain, according the calculated RMSF values, compared to WT, as expected given the negation of its three key stabilizing glue point residues. Note that, as expected, the RMSF values for the S2 domain are the same between WT and the theoretical triple mutant.

### UK variant B.1.1.7.

**FIG 7:**
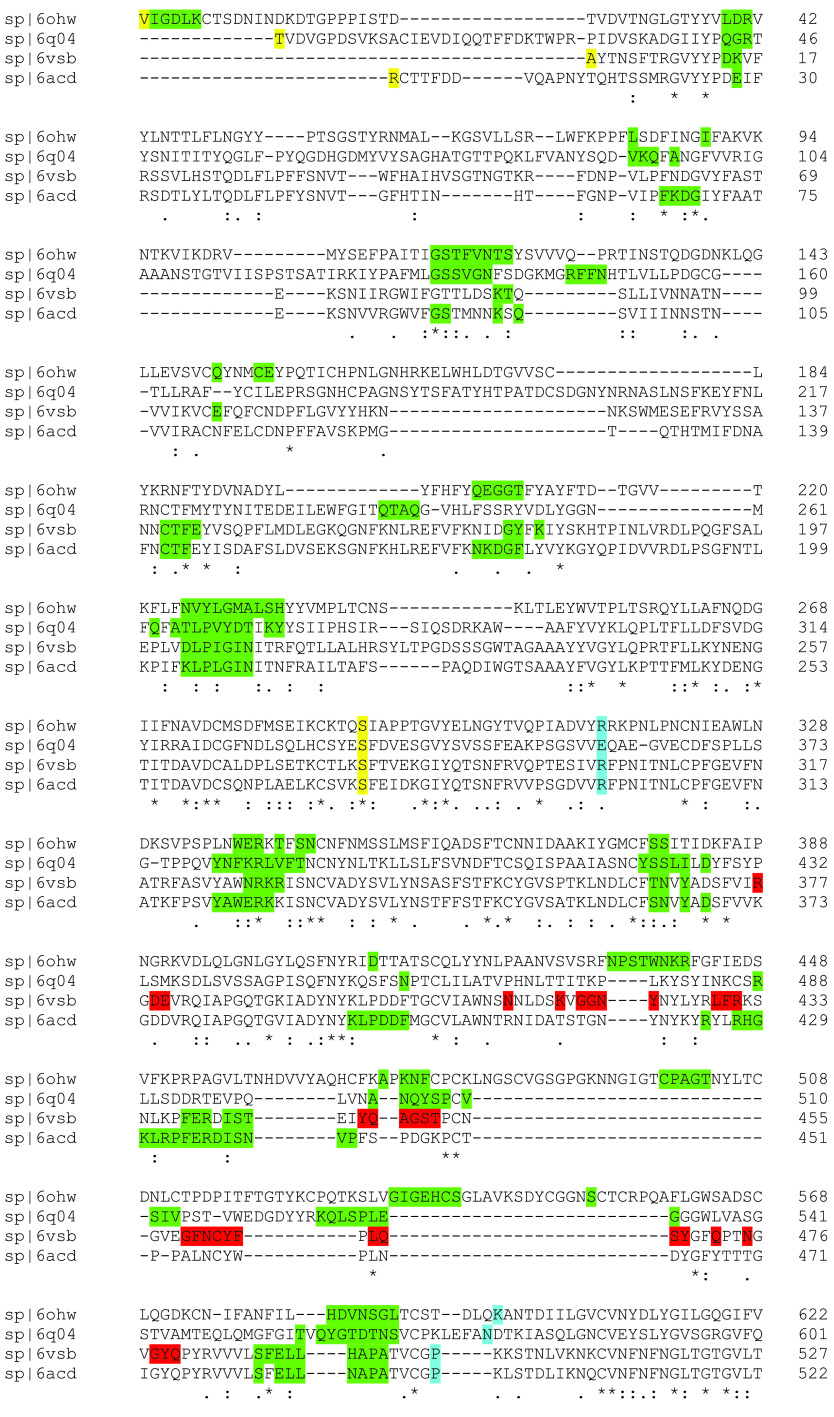
SARS-CoV-2 binding residues to hACE2 (red shaded letters) shown on the combination of the sequence alignment map with the glue point map for the S1 domain across the betacoronaviruses. See Table S3 for the complete data.

A summary of the mutations and deletions of the UK variant B.1.1.7 are given in Table 1. Also shown are the partner glue point residues predicted by OpenContact when the B.1.1.7 residues are in the Up state and mapped to a neighboring Down-state protomer, or in the Down state and mapped to a neighboring Down-state protomer. As can be seen, only A570D and D614G involve glue point partners within the Spike protein. None of the glue point partners are involved in the NTD-RBD sequence homologous regions presented previously. Additionally, we mapped hACE2 binding of SARS-CoV-2 RBD according to the full-length hACE2 structure file PDB ID 6M17 (12). We have overlaid the dominant glue point residues to hACE2 in red, as shown in Fig. 7. The residue N501 is a key binding partner to hACE2 (Table S3) and this includes conspicuously strong interactions with Y41 of ACE2. The N501Y mutation may demonstrate a significant increase in binding to hACE2 due to the highly favorable Y-Y hydrophobic interaction pair in the new mutant state (Table S3); however, this remains to be concretely verified. Note that both D614G and N501Y are also present in the South African variant (B.1.351).

**TABLE 1:** Summary of mutations and deletions of the B.1.1.7 variant

Mutation/deletion	Up → Down	Down → Down	NTD-RBD homologous	ACE2 binding
N501Y			No	Yes
A570D	S2:960-967	S2: 963-967, 875, 1000	No	No
D614G	S2: 854-860	S2: 733-735, 854-861	No	No
P681H			No	No
Deletion V69–S70			No	No
Deletion Y144–Y145			No	No

**FIG 8:**
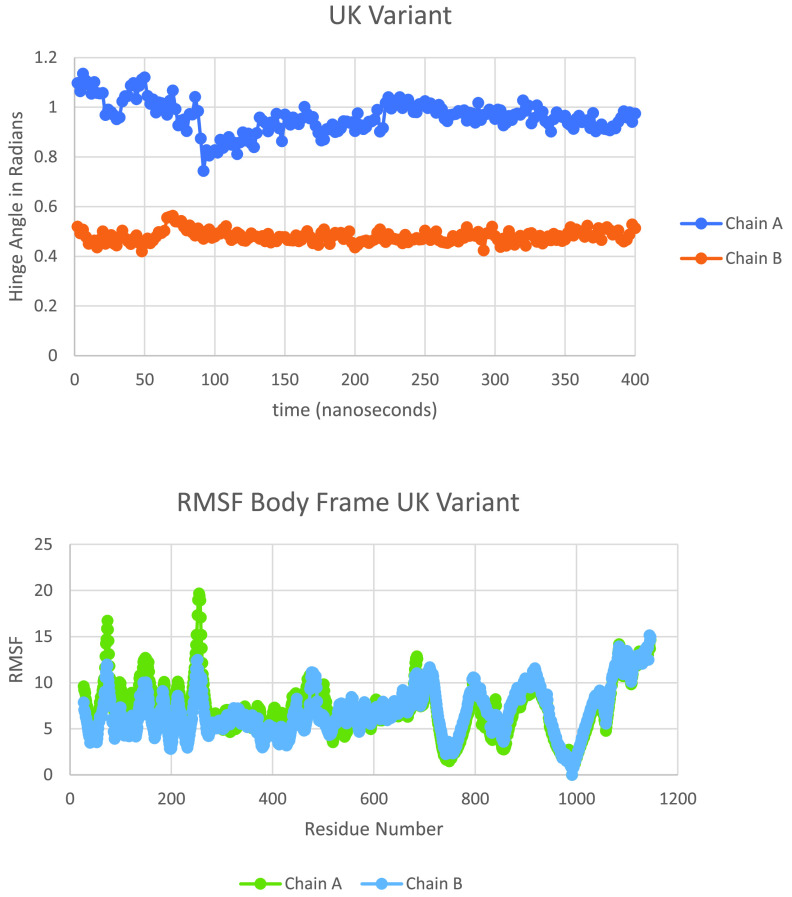
Hinge angle (A) and RMSF values (B) for the UK variant B.1.1.7; chain A is Up and chain B is Down.

**FIG 9:**
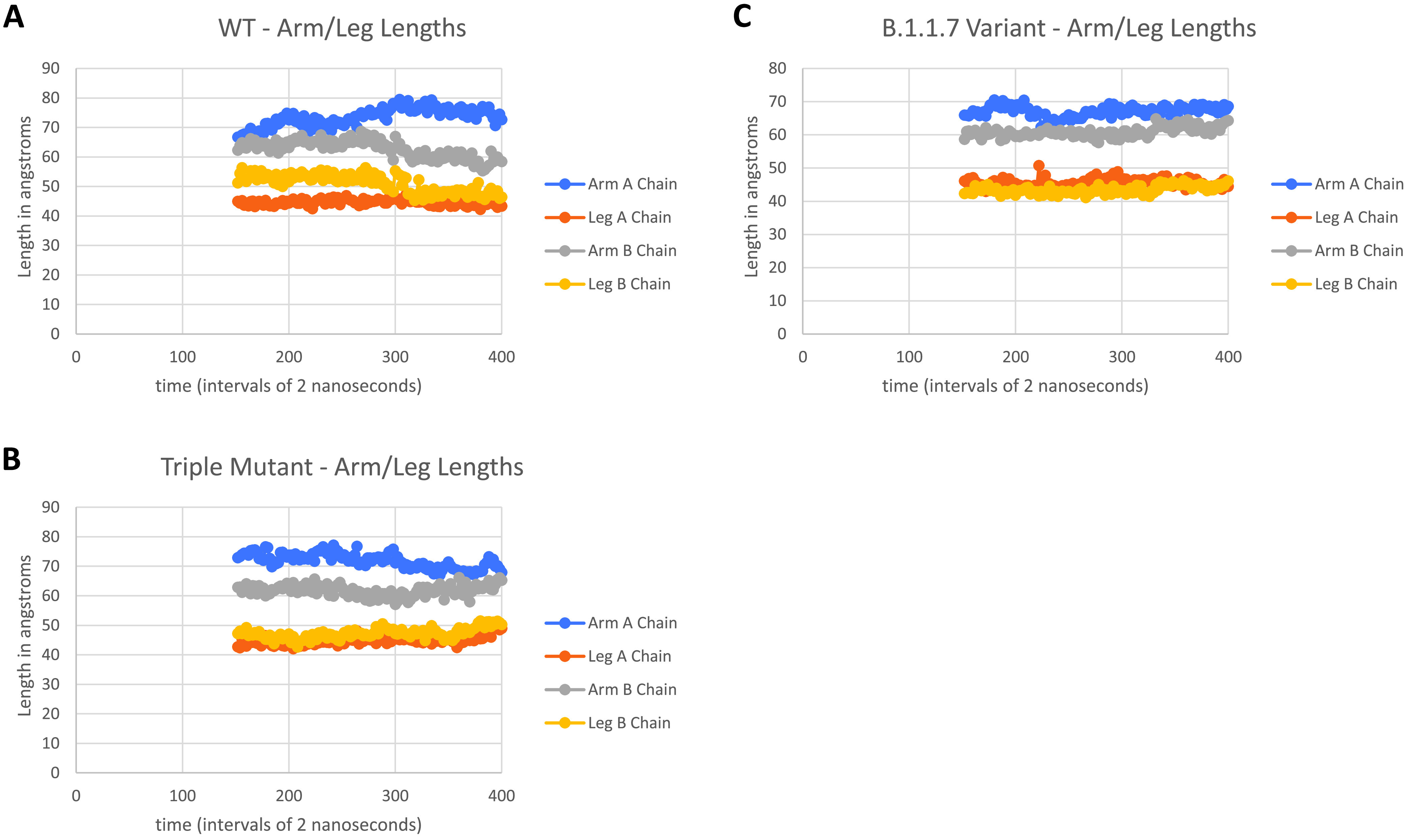
Arm and leg distance calculations after 150 nsec of simulation for WT, triple mutant, and the UK variant B.1.1.7; chain A is Up and chain B is Down for all three trimeric proteins.

Next, we performed long-time MD simulations of B.1.1.7 as described in the Materials and Methods section and shown in Fig. 8 (cf. Fig. 6). As can be seen, the B.1.1.7 variant equilibrates to the WT Up (chain A) and Down (chain B) hinge angles after approximately 150 nsec of simulation. Because of the lack of significant change of hinge angles also observed for this variant, we looked more closely at the specific length measures associated with this angle, i.e., the arm and leg lengths discussed in the Materials and Methods section. Figure 9 shows the results of arm and leg calculations across each of the Spike proteins: WT, triple mutant, and UK variant. The leg lengths associated with the more proximal S1 domain to S2 showed very few differences across all three variants, as expected; however, again, B.1.1.7 demonstrated less overall variability. Similarly, the Up chain arm lengths also showed little changes across the three Spike proteins, with B.1.1.7 again demonstrating less fluctuation. Interestingly, however, the Down chain arm lengths showed conspicuous differences, where both the triple mutant and UK variant demonstrated a longer “reach” compared to WT. Note that deletions associated with B.1.1.7 (Table 1) and modeled by structure-breaking glycine mutations showed increased flexibility of the NTD, as expected.

## DISCUSSION

Despite its relatively low mutation rate and an inherent error correction mechanism, SARS-CoV-2 continues to display significant numbers of variants due to its high transmission and infection rates. Thus, variants represent a considerable challenge in the current COVID-19 pandemic. The identification of key “glue point” residues that help stabilize or maintain the Down-state protomers of the prefusion Spike protein structure could be an important tool in helping to determining structure-to-function relationships. Here, we have demonstrated the presence of “sequence homologous glue points” by overlaying dominant energetic mappings to sequence alignment maps across lineages of betacoronaviruses. SARS-CoV-2 prefusion Spike protein was shown to exhibit the least number of stabilizing glue points across these lineages. Additionally, we analyzed a theoretical triple mutant based on the identification of three key stabilizing glue points between neighboring RBD and NTD interactions in the prefusion Spike protein of SARS-CoV-2. We demonstrated the ability to significantly alter protomer configurations by destabilizing these three key residues through alanine mutations or alanine screening. By the same methods, we then analyzed the emerging UK variant B.1.1.7 in order to determine key mutations or deletions in its Spike protein that could potentially be responsible for Down-to-Up protomer states and presumably a more infective and transmissive state of SARS-CoV-2. Our methodologies directly identify two key mutations (D614G and N501Y) as possible configuration and ACE2 binding changes, respectively. We have previously identified D614 as a key glue point associated with dominant energetic interactions between neighboring protomers (11), which in itself demonstrates the potential of glue point monitoring as a helpful tool in tracking possible variants of interest. Biophysical computations demonstrated the configurational changes associated with D614G, where the arm length of the Down-state transitions to an Up-state value, despite no changes in the hinge angle. The D614 mutation also demonstrates an overall more stable conformation with less conformational fluctuations. We further show that N501Y has a potential hACE2 glue point partner, 41Y, which may lead to a strong Y-Y hydrophobic residue pair interaction; this may be partially responsible for the higher infection rate of the UK (B.1.1.7) and SA (B.1.351) variants, although more studies are needed to verify this. It is clear that many tools are needed to quickly translate genome sequence information from WT and variants to potential virion function in order to help direct mitigation strategies and resources in an optimized way, and to ascertain the impact of variants on vaccines and therapeutics. Here, we have demonstrated that a combination of phylogenetic protein residue sequence alignment superimposed on *ab initio* biophysics of glue point and binding point residue identification can help uncover key differences and similarities across lineages and variants.

**Note added in proof.** After the initial writing of this manuscript, the complete molecular structure of the D614G mutation of the Spike protein of SARS-CoV-2 was determined (18). Those authors also experimentally showed a significantly enhanced stability of the prefusion complex of D614G. It was demonstrated that the less-stable WT configuration leads to premature transitions of the Spike protein from prefusion to postfusion complexes. On the other hand, D614G leads to a reduction in premature transitions compared to WT and increased infection rates. The structures analyzed here involve prefusion stabilized via forced covalent bonding at the S1/S2 interface (19).

## MATERIALS AND METHODS

### Molecular dynamics.

Explicit solvent molecular dynamics (MD) simulations of the novel coronavirus Spike protein were performed using the NAMD2 program (20). We used the CHARMM-Gui (21) with the CHARMM36m force field along with TIP3P water molecules to explicitly solvate proteins and add any missing residues from the experimental structure files. Disulfide bonds and glycosylated sites were all included. Simulations were carried out maintaining the number of simulated particles, pressure, and temperature (the NPT ensemble) constant with the Langevin piston method specifically used to maintain a constant pressure of 1 atm. We employed periodic boundary conditions and initial equilibration for a water box simulation volume, as well as the particle mesh Ewald (PME) method with a 20 Å cutoff distance between the simulated protein and water box edge. The integration time step was 2 femtoseconds, with our protein simulations conducted under physiological conditions (37°C, pH of 7.4, physiological ionic strength with NaCl ions, LYS and ARG were protonated and HIS was not). All mutations were added via the CHARMM-Gui (21) and for deletions we chose to use glycine as a structure-breaking mutation in lieu of deleting residues, since exact structural information on deletions is currently lacking. Any other methods to revise structure, such as the SWISS model (22), would still be approximate and not based on the actual protein folding dynamics, whereas the more straight-forward, structure-breaking glycine represents a good test of the potential role of deletions on structure, as we have demonstrated here. All MD results given here were also repeated several times in order to help confirm trends in data.

### Sequence alignment.

Multiple sequence alignment was preformed using Clustal Omega (23). Clustal Omega uses a structure-guided hidden Markov model (HMM) for multiple sequence alignment. Sequences were obtained directly from the PDB files across four different betacoronaviruses: SARS-CoV (6ACD) (14), SARS-CoV-2 (6VSB) (10), MERS-CoV (6Q04) (24), and HCoV (6OHW) (25). Output format was selected as ClustaW with character counts.

### All-atom energetic mappings.

Previously (11, 15), we analyzed the complete interprotomer and intraprotomer interactions across two independently published structure files (6VSB and 6VYB) for SARS-CoV-2 trimeric Spike protein using the open source energy mapping algorithm developed by Krall et al. (26). This spatial and energetic mapping algorithm efficiently parses the strongest or most dominant noncovalent atom-atom interactions (charge and partial atomic charge, Born, and van der Waals forces), according to empirically established parsing criteria, based on the *ab initio* AMBER03 force field model. Following our previous studies, the parsing criteria were taken as the upper limit of −0.1*kT* units for Lennard-Jones (van der Waals) criteria and −0.3*kT* units for Coulombic interactions, although lower values can also be specified in the analysis part of the mappings in order to further refine the results (26). Note that in the all-atom analysis, dominant van der Waals interaction forces are commonly associated with nonpolar atom-atom interactions and hydrophobic protein interaction regions, whereas the Coulombic partial charge and charge interactions are commonly associated with hydrophilic protein interaction regions and can include hydrogen bonding and backbone atom partial charge interactions.

### RMSF and hinge angle determinations.

Here, we follow the recent hinge angle designation of Peng et al. (17) in order to help quantify and compare Up versus Down protomer states, namely, ∠ASP406-VAL991-ALA622. Note that the vertex selected (VAL991) is in the rigid S2 domain and, therefore, is approximately fixed in the body frame of the protein. This designation helps to correct for any so-called “tumbling” effects associated with translation and rotations of the center of mass of the protein over large time scales necessary for these types of simulations. Those authors further designated hinge angles in the range of 52.2 deg to 84.8 deg as ACE2-accessible domains (“Up” states) and angles in the range of 31.6 deg to 52.2 deg as ACE2-inaccessible (“Down” states). Here, we also define the “arm” and “leg” of the hinge angle as the C-*α* distance between *ASP*406-*V AL*991 and *V AL*991- *ALA*622, respectively.

Root mean square fluctuations (RMSF) C-*α* values across the 1,124 residues for any protomer were determined according to the formula:

(1) $$\mathrm{RMSF}=\frac{1}{N}\sum_{i}^{N}[{\left(x_{i}-x_{991}\right)}^{2}+(y_{i}-y_{991}^{2})+{\left(z_{i}-z_{991}\right)}^{2}]$$

where (*x*, *y*, and *z*) are the cartesian coordinates of any C-*α* residue, *N* is the number of snapshots considered, and deviations are measured relative to the body frame or VAL991 for consistency with hinge angle calculations and to correct for tumbling effects. Here, we take snapshots of structures after every 1.0 nsec.
