# Supplementary material for: Transformations, Lineage Comparisons, and Analysis of Down-to-Up Protomer States of Variants of the SARS-CoV-2 Prefusion Spike Protein, Including the UK Variant B.1.1.7
Source: Microbiol Spectr. 2021 Aug 4;9(1):10.1128/spectrum.00030-21. doi: 10.1128/spectrum.00030-21 (PMC8562339; doi:10.1128/spectrum.00030-21)
Supplement: SUPPLEMENTAL FILE 1 — Supplemental material. Download SPECTRUM00030-21_Supp_2_seq14.pdf, PDF file, 0.1 MB [file spectrum00030-21_supp_2_seq14.pdf]

Table Headings for Supplemmentary Data - From OpenContact: open source code contactgui.f:

Distances rrs are in nanometers
Energies are in kT units
us148 are LJ 6-12 interactions
ucouls are partial atomic charge interactions
All atom force field parameters from AMBER 03

```
c write fine contact map for matplotlib
c
       write(16,127)resa(j),',',resnma(j),',',atnama(j),',',
     1                     atnuma(j),',', resb(i),',',resnmb(i),',',
     1                     atnamb(i),',',atnumb(i),',',
     1                     rrs,',',us148,',',ucouls
 127   format(a3,a1,i6,a1,a4,a1,i6,a1,a3,a1,i6,a1,a4,a1,i6,a1,e15.8,a1,
     1                     e15.8,a1,e15.8)
```

when citing OpenContact please use"

Krall,A., Brunn, J., Kankanala, S., and Peters, M.H. A Simple Contact Mapping Algorithm for Identifying Peptide Mimetics in Protein-Protein Interaction Partners. \emph{Proteins. Structure, Function and Bioinformatics}, 82, 2253-2262, 2014. DOI:10.1002/prot.24592.

To Download OpenContact go to the PDB website under third party software or visit:
http://people.vcu.edu/~mpeters

Table S1 6ACD-B-RBDtoA-NTD-finedata.txt

TYR	338	C	10827	GLN	112	OE1	771	0.951E+00	-0.324E+00	-0.122E-02
TYR	338	C	10827	GLN	112	NE2	772	0.896E+00	-0.500E+00	-0.204E-02
TYR	338	O	10828	GLN	112	CD	770	0.795E+00	-0.392E+00	-0.356E-02
ALA	339	C	10839	GLN	112	NE2	772	0.105E+01	-0.361E+00	-0.799E-03
TRP	340	C	10844	LEU	224	O	1671	0.846E+00	-0.326E+00	-0.246E-02
GLU	341	CD	10862	CYS	159	O	1134	0.825E+00	-0.467E+00	-0.285E-02
GLU	341	CD	10862	THR	160	O	1140	0.743E+00	-0.532E+00	-0.534E-02
GLU	341	CD	10862	THR	160	OG1	1142	0.976E+00	-0.367E+00	-0.115E-02
GLU	341	OE1	10863	CYS	159	C	1133	0.982E+00	-0.418E+00	-0.100E-02
GLU	341	OE1	10863	THR	160	C	1139	0.924E+00	-0.400E+00	-0.145E-02
GLU	341	OE2	10864	CYS	159	C	1133	0.877E+00	-0.499E+00	-0.197E-02
GLU	341	OE2	10864	THR	160	C	1139	0.770E+00	-0.544E+00	-0.430E-02
GLU	341	OE2	10864	PHE	161	C	1146	0.104E+01	-0.326E+00	-0.694E-03
ARG	342	C	10867	THR	160	O	1140	0.820E+00	-0.425E+00	-0.295E-02
ARG	342	C	10867	THR	160	OG1	1142	0.988E+00	-0.344E+00	-0.107E-02
ARG	342	C	10867	PRO	223	O	1664	0.685E+00	-0.467E+00	-0.864E-02
ARG	342	C	10867	LEU	224	O	1671	0.802E+00	-0.447E+00	-0.337E-02
ARG	342	O	10868	THR	160	C	1139	0.787E+00	-0.367E+00	-0.379E-02
ARG	342	O	10868	LEU	224	C	1670	0.830E+00	-0.342E+00	-0.275E-02
ARG	342	CD	10871	PRO	223	C	1663	0.438E+00	0.170E+00	-0.108E+00
ARG	342	CD	10871	PRO	223	O	1664	0.327E+00	-0.481E+00	-0.127E+00
ARG	342	CD	10871	PRO	223	CB	1665	0.419E+00	-0.170E-02	-0.145E+00
ARG	342	NE	10872	ASP	191	O	1401	0.686E+00	-0.341E+00	-0.104E-01
ARG	342	NE	10872	GLY	192	O	1409	0.664E+00	-0.370E+00	-0.127E-01
ARG	342	NE	10872	PHE	193	CE1	1418	0.437E+00	-0.295E+00	-0.122E+00
ARG	342	NE	10872	LEU	222	O	1656	0.673E+00	-0.395E+00	-0.117E-01
ARG	342	NE	10872	PRO	223	CA	1662	0.431E+00	-0.682E-01	-0.147E+00
ARG	342	NE	10872	PRO	223	C	1663	0.425E+00	0.673E+00	-0.138E+00
ARG	342	NE	10872	PRO	223	O	1664	0.336E+00	-0.165E+01	-0.288E+00
ARG	342	NE	10872	PRO	223	CB	1665	0.366E+00	-0.895E-02	-0.218E+00
ARG	342	NE	10872	LEU	224	N	1668	0.552E+00	-0.382E+00	-0.440E-01

64	ARG	342	NE	10872	LEU	224	O	1671	0.573E+00	-0.553E+00	-0.302E-01
65	ARG	342	CZ	10873	LYS	190	O	1392	0.871E+00	-0.304E+00	-0.206E-02
66	ARG	342	CZ	10873	ASP	191	O	1401	0.604E+00	-0.539E+00	-0.181E-01
67	ARG	342	CZ	10873	ASP	191	OD2	1405	0.101E+01	-0.312E+00	-0.835E-03
68	ARG	342	CZ	10873	GLY	192	O	1409	0.670E+00	-0.442E+00	-0.984E-02
69	ARG	342	CZ	10873	PHE	193	N	1410	0.675E+00	-0.317E+00	-0.110E-01
70	ARG	342	CZ	10873	PHE	193	CE1	1418	0.421E+00	-0.396E+00	-0.112E+00
71	ARG	342	CZ	10873	LEU	222	O	1656	0.725E+00	-0.417E+00	-0.619E-02
72	ARG	342	CZ	10873	PRO	223	O	1664	0.427E+00	-0.106E+01	-0.123E+00
73	ARG	342	CZ	10873	LEU	224	N	1668	0.636E+00	-0.343E+00	-0.157E-01
74	ARG	342	CZ	10873	LEU	224	O	1671	0.639E+00	-0.533E+00	-0.130E-01
75	ARG	342	NH1	10874	LYS	190	C	1391	0.993E+00	-0.386E+00	-0.111E-02
76	ARG	342	NH1	10874	ASP	191	C	1400	0.724E+00	-0.402E+00	-0.732E-02
77	ARG	342	NH1	10874	ASP	191	CG	1403	0.981E+00	-0.404E+00	-0.119E-02
78	ARG	342	NH1	10874	GLY	192	C	1408	0.698E+00	-0.565E+00	-0.906E-02
79	ARG	342	NH1	10874	PHE	193	C	1412	0.939E+00	-0.318E+00	-0.155E-02
80	ARG	342	NH1	10874	LEU	222	C	1655	0.847E+00	-0.392E+00	-0.286E-02
81	ARG	342	NH1	10874	PRO	223	C	1663	0.596E+00	-0.448E+00	-0.229E-01
82	ARG	342	NH1	10874	LEU	224	C	1670	0.694E+00	-0.564E+00	-0.941E-02
83	ARG	342	NH1	10874	GLY	225	C	1678	0.837E+00	-0.406E+00	-0.308E-02
84	ARG	342	NH2	10875	LYS	190	C	1391	0.886E+00	-0.462E+00	-0.219E-02
85	ARG	342	NH2	10875	ASP	191	C	1400	0.663E+00	-0.476E+00	-0.123E-01
86	ARG	342	NH2	10875	ASP	191	CG	1403	0.881E+00	-0.478E+00	-0.227E-02
87	ARG	342	NH2	10875	GLY	192	C	1408	0.636E+00	-0.681E+00	-0.157E-01
88	ARG	342	NH2	10875	PHE	193	C	1412	0.819E+00	-0.397E+00	-0.349E-02
89	ARG	342	NH2	10875	PHE	193	CD1	1416	0.429E+00	0.241E+00	-0.133E+00
90	ARG	342	NH2	10875	PHE	193	CZ	1420	0.373E+00	0.418E+00	-0.196E+00
91	ARG	342	NH2	10875	LYS	221	C	1646	0.926E+00	-0.430E+00	-0.168E-02
92	ARG	342	NH2	10875	LEU	222	C	1655	0.722E+00	-0.523E+00	-0.744E-02
93	ARG	342	NH2	10875	PRO	223	C	1663	0.550E+00	-0.536E+00	-0.365E-01
94	ARG	342	NH2	10875	LEU	224	C	1670	0.732E+00	-0.509E+00	-0.683E-02
95	ARG	342	NH2	10875	GLY	225	C	1678	0.912E+00	-0.353E+00	-0.185E-02
96	LYS	343	C	10878	THR	160	O	1140	0.101E+01	-0.303E+00	-0.849E-03
97	LYS	343	C	10878	PRO	223	O	1664	0.865E+00	-0.304E+00	-0.215E-02
98	SER	380	C	11170	GLU	45	OE1	228	0.100E+01	-0.304E+00	-0.881E-03
99	SER	380	O	11171	GLU	45	CD	227	0.105E+01	-0.319E+00	-0.674E-03
100	SER	380	OG	11173	GLU	45	CD	227	0.796E+00	-0.545E+00	-0.391E-02
101	ASN	381	CG	11179	GLU	45	OE1	228	0.992E+00	-0.374E+00	-0.944E-03
102	ASN	381	CG	11179	GLU	45	OE2	229	0.983E+00	-0.380E+00	-0.100E-02
103	ASN	381	CG	11179	LYS	221	O	1647	0.852E+00	-0.325E+00	-0.235E-02
104	ASN	381	ND2	11181	GLU	45	CD	227	0.903E+00	-0.538E+00	-0.195E-02
105	ASN	381	ND2	11181	PHE	220	C	1635	0.103E+01	-0.317E+00	-0.904E-03
106	ASN	381	ND2	11181	LYS	221	C	1646	0.885E+00	-0.526E+00	-0.220E-02
107	ASN	381	ND2	11181	LEU	222	C	1655	0.105E+01	-0.322E+00	-0.802E-03
108	TYR	383	CD2	11196	PHE	193	CE1	1418	0.429E+00	0.122E+00	-0.104E+00
109	TYR	383	CD2	11196	PHE	193	CZ	1420	0.412E+00	0.860E-01	-0.120E+00
110	TYR	383	CE2	11198	PHE	193	CE1	1418	0.373E+00	0.175E+00	-0.137E+00
111	TYR	383	CE2	11198	PRO	223	CB	1665	0.408E+00	0.263E-02	-0.140E+00
112	TYR	383	CZ	11199	PRO	223	CB	1665	0.406E+00	-0.303E-02	-0.143E+00
113	TYR	383	OH	11200	LYS	221	C	1646	0.618E+00	-0.554E+00	-0.176E-01
114	TYR	383	OH	11200	LEU	222	C	1655	0.601E+00	-0.463E+00	-0.206E-01
115	TYR	383	OH	11200	PRO	223	CB	1665	0.403E+00	0.630E-02	-0.193E+00
116	TYR	383	OH	11200	PRO	223	CG	1666	0.416E+00	-0.251E-01	-0.169E+00
117	TYR	383	OH	11200	PRO	223	CD	1667	0.464E+00	0.176E-01	-0.100E+00
118	ASP	385	CG	11211	ASP	191	O	1401	0.968E+00	-0.301E+00	-0.109E-02
119	ASP	385	CG	11211	PRO	223	O	1664	0.809E+00	-0.350E+00	-0.321E-02
120	ASP	385	CG	11211	LEU	224	O	1671	0.906E+00	-0.372E+00	-0.163E-02
121	ASP	385	OD1	11212	LEU	224	C	1670	0.925E+00	-0.362E+00	-0.144E-02
122	ASP	385	OD2	11213	GLY	192	C	1408	0.103E+01	-0.313E+00	-0.757E-03
123	ASP	385	OD2	11213	LEU	224	C	1670	0.993E+00	-0.325E+00	-0.941E-03
124	LYS	411	C	11407	ASP	191	N	1398	0.102E+01	-0.301E+00	-0.928E-03
125	LYS	411	C	11407	ASP	191	O	1401	0.825E+00	-0.379E+00	-0.285E-02
126	LYS	411	C	11407	ASP	191	OD1	1404	0.953E+00	-0.437E+00	-0.120E-02
127	LYS	411	O	11408	ASP	191	C	1400	0.761E+00	-0.300E+00	-0.461E-02
128	LYS	411	O	11408	ASP	191	CG	1403	0.918E+00	-0.367E+00	-0.150E-02
129	LEU	412	C	11416	ASP	191	O	1401	0.776E+00	-0.334E+00	-0.410E-02

130	LEU	412	C	11416	ASP	191	OD1	1404	0.767E+00	-0.496E+00	-0.440E-02
131	LEU	412	C	11416	ASP	191	OD2	1405	0.873E+00	-0.398E+00	-0.204E-02
132	LEU	412	O	11417	ASP	191	CG	1403	0.834E+00	-0.426E+00	-0.268E-02
133	PRO	413	C	11424	ASP	191	OD1	1404	0.714E+00	-0.331E+00	-0.676E-02
134	PRO	413	O	11425	ASP	191	CG	1403	0.854E+00	-0.319E+00	-0.231E-02
135	ASP	414	N	11429	ASP	191	CG	1403	0.743E+00	-0.523E+00	-0.624E-02
136	ASP	414	C	11431	ASP	191	OD1	1404	0.880E+00	-0.303E+00	-0.194E-02
137	ASP	414	CG	11434	ASP	191	OD1	1404	0.879E+00	-0.510E+00	-0.195E-02
138	ASP	414	CG	11434	ASP	191	OD2	1405	0.980E+00	-0.431E+00	-0.102E-02
139	ASP	414	OD1	11435	ASP	191	CG	1403	0.995E+00	-0.421E+00	-0.931E-03
140	ASP	414	OD2	11436	ASP	191	CG	1403	0.958E+00	-0.446E+00	-0.117E-02
141	ASP	415	N	11437	ASP	191	CG	1403	0.817E+00	-0.442E+00	-0.356E-02
142	ASP	415	C	11439	ASP	191	OD2	1405	0.825E+00	-0.338E+00	-0.285E-02
143	ASP	415	CG	11442	ASP	191	OD1	1404	0.918E+00	-0.476E+00	-0.150E-02
144	ASP	415	CG	11442	ASP	191	OD2	1405	0.870E+00	-0.520E+00	-0.208E-02
145	ASP	415	OD1	11443	ASP	191	CG	1403	0.103E+01	-0.403E+00	-0.777E-03
146	ASP	415	OD2	11444	ASP	191	CG	1403	0.900E+00	-0.492E+00	-0.170E-02
147	PHE	416	N	11445	ASP	191	CG	1403	0.753E+00	-0.340E+00	-0.579E-02
148	PHE	416	C	11447	ASP	191	OD1	1404	0.892E+00	-0.367E+00	-0.179E-02
149	PHE	416	C	11447	ASP	191	OD2	1405	0.808E+00	-0.433E+00	-0.322E-02
150	PHE	416	O	11448	ASP	191	CG	1403	0.795E+00	-0.421E+00	-0.357E-02
151	PHE	416	CD2	11452	ASP	191	OD2	1405	0.426E+00	0.261E+00	-0.124E+00
152	PHE	416	CE2	11454	ASP	191	OD2	1405	0.434E+00	0.472E+00	-0.114E+00
153	ARG	441	NE	11655	GLN	112	NE2	772	0.843E+00	-0.413E+00	-0.360E-02
154	ARG	441	CZ	11656	GLN	112	OE1	771	0.783E+00	-0.407E+00	-0.391E-02
155	ARG	441	CZ	11656	GLN	112	NE2	772	0.747E+00	-0.622E+00	-0.604E-02
156	ARG	441	CZ	11656	ASN	227	ND2	1695	0.101E+01	-0.336E+00	-0.101E-02
157	ARG	441	NH1	11657	LYS	110	C	751	0.929E+00	-0.428E+00	-0.165E-02
158	ARG	441	NH1	11657	GLN	112	CD	770	0.683E+00	-0.678E+00	-0.103E-01
159	ARG	441	NH2	11658	LYS	110	C	751	0.995E+00	-0.385E+00	-0.109E-02
160	ARG	441	NH2	11658	GLN	112	CD	770	0.846E+00	-0.458E+00	-0.288E-02
161	ARG	444	C	11681	ASN	227	ND2	1695	0.979E+00	-0.453E+00	-0.120E-02
162	ARG	444	NE	11686	ASN	227	OD1	1694	0.710E+00	-0.336E+00	-0.853E-02
163	ARG	444	NE	11686	ASN	227	ND2	1695	0.586E+00	-0.739E+00	-0.312E-01
164	ARG	444	CZ	11687	SER	105	OG	717	0.837E+00	-0.369E+00	-0.289E-02
165	ARG	444	CZ	11687	ASN	227	OD1	1694	0.622E+00	-0.533E+00	-0.153E-01
166	ARG	444	CZ	11687	ASN	227	ND2	1695	0.518E+00	-0.119E+01	-0.511E-01
167	ARG	444	NH1	11688	ASN	227	C	1690	0.965E+00	-0.343E+00	-0.131E-02
168	ARG	444	NH1	11688	ASN	227	CG	1693	0.666E+00	-0.623E+00	-0.120E-01
169	ARG	444	NH2	11689	GLN	112	CD	770	0.104E+01	-0.334E+00	-0.853E-03
170	ARG	444	NH2	11689	GLY	225	C	1678	0.970E+00	-0.321E+00	-0.127E-02
171	ARG	444	NH2	11689	ILE	226	C	1682	0.798E+00	-0.432E+00	-0.409E-02
172	ARG	444	NH2	11689	ASN	227	C	1690	0.750E+00	-0.524E+00	-0.593E-02
173	ARG	444	NH2	11689	ASN	227	CG	1693	0.454E+00	-0.148E+01	-0.103E+00
174	ARG	444	NH2	11689	ASN	227	ND2	1695	0.394E+00	0.284E+01	-0.239E+00
175	ARG	444	NH2	11689	ILE	228	C	1698	0.946E+00	-0.326E+00	-0.147E-02
176	HIS	445	C	11692	ASN	227	ND2	1695	0.860E+00	-0.504E+00	-0.262E-02
177	HIS	445	O	11693	ASN	227	CG	1693	0.859E+00	-0.301E+00	-0.223E-02
178	GLY	446	C	11702	ASN	227	ND2	1695	0.926E+00	-0.393E+00	-0.168E-02
179	LYS	447	C	11706	ASN	227	OD1	1694	0.900E+00	-0.345E+00	-0.169E-02
180	LYS	447	C	11706	ASN	227	ND2	1695	0.878E+00	-0.533E+00	-0.231E-02
181	LYS	447	O	11707	ASN	227	CG	1693	0.799E+00	-0.363E+00	-0.346E-02
182	LEU	448	C	11715	ASN	189	ND2	1388	0.101E+01	-0.342E+00	-0.102E-02
183	LEU	448	C	11715	ASP	191	OD1	1404	0.102E+01	-0.314E+00	-0.821E-03
184	LEU	448	C	11715	ASN	227	OD1	1694	0.848E+00	-0.301E+00	-0.242E-02
185	LEU	448	C	11715	ASN	227	ND2	1695	0.902E+00	-0.403E+00	-0.196E-02
186	ARG	449	C	11723	ASN	189	OD1	1387	0.865E+00	-0.371E+00	-0.215E-02
187	ARG	449	C	11723	ASN	189	ND2	1388	0.836E+00	-0.583E+00	-0.310E-02
188	ARG	449	C	11723	LYS	190	O	1392	0.924E+00	-0.357E+00	-0.145E-02
189	ARG	449	C	11723	ASP	191	N	1398	0.739E+00	-0.519E+00	-0.648E-02
190	ARG	449	C	11723	ASP	191	O	1401	0.711E+00	-0.500E+00	-0.691E-02
191	ARG	449	C	11723	ASP	191	OD1	1404	0.756E+00	-0.651E+00	-0.482E-02
192	ARG	449	C	11723	ASP	191	OD2	1405	0.949E+00	-0.443E+00	-0.123E-02
193	ARG	449	C	11723	GLY	192	N	1406	0.646E+00	-0.452E+00	-0.144E-01
194	ARG	449	C	11723	GLY	192	O	1409	0.904E+00	-0.334E+00	-0.165E-02
195	ARG	449	C	11723	GLY	225	O	1679	0.825E+00	-0.388E+00	-0.285E-02

196	ARG	449 C	11723	ILE	226 O	1683	0.856E+00	-0.444E+00	-0.228E-02
197	ARG	449 C	11723	ASN	227 OD1	1694	0.876E+00	-0.363E+00	-0.199E-02
198	ARG	449 C	11723	ASN	227 ND2	1695	0.981E+00	-0.452E+00	-0.119E-02
199	ARG	449 O	11724	LYS	190 C	1391	0.922E+00	-0.364E+00	-0.146E-02
200	ARG	449 O	11724	ASP	191 C	1400	0.703E+00	-0.358E+00	-0.738E-02
201	ARG	449 O	11724	ASP	191 CG	1403	0.906E+00	-0.386E+00	-0.163E-02
202	ARG	449 O	11724	GLY	192 C	1408	0.845E+00	-0.337E+00	-0.248E-02
203	ARG	449 O	11724	GLY	225 C	1678	0.786E+00	-0.382E+00	-0.382E-02
204	ARG	449 O	11724	ILE	226 C	1682	0.852E+00	-0.325E+00	-0.235E-02
205	ARG	449 O	11724	ASN	227 CG	1693	0.889E+00	-0.311E+00	-0.182E-02
206	ARG	449 NE	11728	ASP	85 OD1	561	0.673E+00	-0.517E+00	-0.117E-01
207	ARG	449 NE	11728	ASP	85 OD2	562	0.853E+00	-0.335E+00	-0.284E-02
208	ARG	449 NE	11728	ASN	189 OD1	1387	0.636E+00	-0.418E+00	-0.163E-01
209	ARG	449 NE	11728	ASN	189 ND2	1388	0.479E+00	-0.117E+01	-0.971E-01
210	ARG	449 NE	11728	ILE	226 O	1683	0.571E+00	-0.619E+00	-0.308E-01
211	ARG	449 NE	11728	ASN	227 N	1688	0.646E+00	-0.331E+00	-0.176E-01
212	ARG	449 NE	11728	ASN	227 O	1691	0.713E+00	-0.331E+00	-0.829E-02
213	ARG	449 NE	11728	ASN	227 OD1	1694	0.450E+00	-0.923E+00	-0.118E+00
214	ARG	449 NE	11728	ASN	227 ND2	1695	0.631E+00	-0.630E+00	-0.201E-01
215	ARG	449 NE	11728	ILE	228 N	1696	0.578E+00	-0.438E+00	-0.337E-01
216	ARG	449 NE	11728	ILE	228 O	1699	0.602E+00	-0.552E+00	-0.226E-01
217	ARG	449 CZ	11729	ASP	85 N	555	0.778E+00	-0.365E+00	-0.474E-02
218	ARG	449 CZ	11729	ASP	85 OD1	561	0.634E+00	-0.710E+00	-0.137E-01
219	ARG	449 CZ	11729	ASP	85 OD2	562	0.829E+00	-0.428E+00	-0.278E-02
220	ARG	449 CZ	11729	SER	105 OG	717	0.866E+00	-0.348E+00	-0.235E-02
221	ARG	449 CZ	11729	ASN	189 O	1384	0.774E+00	-0.347E+00	-0.418E-02
222	ARG	449 CZ	11729	ASN	189 OD1	1387	0.565E+00	-0.657E+00	-0.269E-01
223	ARG	449 CZ	11729	ASN	189 ND2	1388	0.380E+00	-0.258E+01	-0.194E+00
224	ARG	449 CZ	11729	ASP	191 N	1398	0.785E+00	-0.359E+00	-0.449E-02
225	ARG	449 CZ	11729	ASP	191 OD1	1404	0.971E+00	-0.332E+00	-0.108E-02
226	ARG	449 CZ	11729	GLY	225 O	1679	0.789E+00	-0.326E+00	-0.374E-02
227	ARG	449 CZ	11729	ILE	226 N	1680	0.713E+00	-0.347E+00	-0.799E-02
228	ARG	449 CZ	11729	ILE	226 O	1683	0.488E+00	-0.108E+01	-0.618E-01
229	ARG	449 CZ	11729	ASN	227 N	1688	0.577E+00	-0.511E+00	-0.277E-01
230	ARG	449 CZ	11729	ASN	227 O	1691	0.614E+00	-0.545E+00	-0.165E-01
231	ARG	449 CZ	11729	ASN	227 OD1	1694	0.397E+00	-0.154E+01	-0.169E+00
232	ARG	449 CZ	11729	ASN	227 ND2	1695	0.605E+00	-0.838E+00	-0.209E-01
233	ARG	449 CZ	11729	ILE	228 N	1696	0.462E+00	-0.899E+00	-0.937E-01
234	ARG	449 CZ	11729	ILE	228 O	1699	0.476E+00	-0.115E+01	-0.704E-01
235	ARG	449 CZ	11729	THR	229 OG1	1709	0.825E+00	-0.356E+00	-0.316E-02
236	ARG	449 NH1	11730	PHE	83 C	537	0.850E+00	-0.374E+00	-0.281E-02
237	ARG	449 NH1	11730	LYS	84 C	548	0.776E+00	-0.578E+00	-0.483E-02
238	ARG	449 NH1	11730	ASP	85 C	557	0.765E+00	-0.363E+00	-0.527E-02
239	ARG	449 NH1	11730	ASP	85 CG	560	0.643E+00	-0.855E+00	-0.148E-01
240	ARG	449 NH1	11730	GLY	86 C	565	0.995E+00	-0.309E+00	-0.109E-02
241	ARG	449 NH1	11730	GLY	104 C	710	0.101E+01	-0.302E+00	-0.998E-03
242	ARG	449 NH1	11730	LYS	188 C	1374	0.846E+00	-0.498E+00	-0.289E-02
243	ARG	449 NH1	11730	ASN	189 C	1383	0.649E+00	-0.694E+00	-0.140E-01
244	ARG	449 NH1	11730	ASN	189 CG	1386	0.423E+00	-0.177E+01	-0.142E+00
245	ARG	449 NH1	11730	LYS	190 C	1391	0.788E+00	-0.563E+00	-0.440E-02
246	ARG	449 NH1	11730	ASP	191 C	1400	0.823E+00	-0.319E+00	-0.340E-02
247	ARG	449 NH1	11730	ASP	191 CG	1403	0.100E+01	-0.391E+00	-0.104E-02
248	ARG	449 NH1	11730	GLY	192 C	1408	0.824E+00	-0.417E+00	-0.338E-02
249	ARG	449 NH1	11730	PHE	193 C	1412	0.902E+00	-0.339E+00	-0.197E-02
250	ARG	449 NH1	11730	GLY	225 C	1678	0.823E+00	-0.418E+00	-0.340E-02
251	ARG	449 NH1	11730	ILE	226 C	1682	0.639E+00	-0.660E+00	-0.152E-01
252	ARG	449 NH1	11730	ASN	227 C	1690	0.564E+00	-0.934E+00	-0.315E-01
253	ARG	449 NH1	11730	ASN	227 CG	1693	0.599E+00	-0.776E+00	-0.223E-01
254	ARG	449 NH1	11730	ILE	228 C	1698	0.521E+00	-0.103E+01	-0.496E-01
255	ARG	449 NH1	11730	ILE	228 O	1699	0.434E+00	0.175E+01	-0.142E+00
256	ARG	449 NH1	11730	THR	229 C	1706	0.803E+00	-0.420E+00	-0.392E-02
257	ARG	449 NH2	11731	PHE	83 C	537	0.953E+00	-0.311E+00	-0.141E-02
258	ARG	449 NH2	11731	LYS	84 C	548	0.940E+00	-0.420E+00	-0.154E-02
259	ARG	449 NH2	11731	ASP	85 CG	560	0.853E+00	-0.505E+00	-0.275E-02
260	ARG	449 NH2	11731	GLY	104 C	710	0.875E+00	-0.377E+00	-0.235E-02
261	ARG	449 NH2	11731	LYS	188 C	1374	0.988E+00	-0.389E+00	-0.114E-02

262	ARG	449	NH2	11731	ASN	189	C	1383	0.809E+00	-0.457E+00	-0.376E-02
263	ARG	449	NH2	11731	ASN	189	CG	1386	0.529E+00	-0.102E+01	-0.452E-01
264	ARG	449	NH2	11731	ASN	189	ND2	1388	0.400E+00	0.272E+01	-0.226E+00
265	ARG	449	NH2	11731	LYS	190	C	1391	0.905E+00	-0.445E+00	-0.192E-02
266	ARG	449	NH2	11731	GLY	192	C	1408	0.827E+00	-0.414E+00	-0.330E-02
267	ARG	449	NH2	11731	PHE	193	C	1412	0.951E+00	-0.312E+00	-0.144E-02
268	ARG	449	NH2	11731	LEU	224	C	1670	0.960E+00	-0.321E+00	-0.135E-02
269	ARG	449	NH2	11731	GLY	225	C	1678	0.678E+00	-0.598E+00	-0.108E-01
270	ARG	449	NH2	11731	ILE	226	C	1682	0.455E+00	-0.143E+01	-0.102E+00
271	ARG	449	NH2	11731	ILE	226	O	1683	0.384E+00	0.241E+01	-0.247E+00
272	ARG	449	NH2	11731	ASN	227	N	1688	0.449E+00	0.111E+01	-0.136E+00
273	ARG	449	NH2	11731	ASN	227	CA	1689	0.353E+00	-0.220E+00	-0.184E+00
274	ARG	449	NH2	11731	ASN	227	C	1690	0.387E+00	-0.235E+01	-0.189E+00
275	ARG	449	NH2	11731	ASN	227	CB	1692	0.430E+00	0.272E+00	-0.149E+00
276	ARG	449	NH2	11731	ASN	227	CG	1693	0.394E+00	-0.212E+01	-0.182E+00
277	ARG	449	NH2	11731	ILE	228	N	1696	0.349E+00	0.226E+01	-0.252E+00
278	ARG	449	NH2	11731	ILE	228	CA	1697	0.455E+00	0.256E+00	-0.114E+00
279	ARG	449	NH2	11731	ILE	228	C	1698	0.464E+00	-0.136E+01	-0.921E-01
280	ARG	449	NH2	11731	ILE	228	O	1699	0.426E+00	0.183E+01	-0.156E+00
281	ARG	449	NH2	11731	THR	229	C	1706	0.770E+00	-0.453E+00	-0.505E-02
282	ARG	449	NH2	11731	ASN	230	C	1713	0.104E+01	-0.308E+00	-0.844E-03
283	PRO	450	C	11734	ASP	191	N	1398	0.622E+00	-0.333E+00	-0.178E-01
284	PRO	450	C	11734	ASP	191	O	1401	0.463E+00	-0.587E+00	-0.820E-01
285	PRO	450	C	11734	ASP	191	OD1	1404	0.652E+00	-0.396E+00	-0.116E-01
286	PRO	450	C	11734	GLY	192	N	1406	0.472E+00	-0.419E+00	-0.846E-01
287	PRO	450	O	11735	ASN	189	CG	1386	0.756E+00	-0.310E+00	-0.479E-02
288	PRO	450	O	11735	LYS	190	C	1391	0.628E+00	-0.553E+00	-0.145E-01
289	PRO	450	O	11735	ASP	191	CA	1399	0.429E+00	-0.129E-01	-0.136E+00
290	PRO	450	O	11735	ASP	191	C	1400	0.368E+00	-0.122E+01	-0.212E+00
291	PRO	450	O	11735	ASP	191	O	1401	0.391E+00	0.117E+01	-0.208E+00
292	PRO	450	O	11735	ASP	191	CG	1403	0.624E+00	-0.575E+00	-0.150E-01
293	PRO	450	O	11735	GLY	192	N	1406	0.358E+00	0.111E+01	-0.299E+00
294	PRO	450	O	11735	GLY	192	CA	1407	0.394E+00	0.296E+00	-0.197E+00
295	PRO	450	O	11735	GLY	192	C	1408	0.545E+00	-0.604E+00	-0.332E-01
296	PRO	450	O	11735	GLY	225	C	1678	0.680E+00	-0.377E+00	-0.905E-02
297	PHE	451	N	11739	ASP	191	C	1400	0.529E+00	-0.420E+00	-0.456E-01
298	PHE	451	N	11739	ASP	191	CG	1403	0.768E+00	-0.328E+00	-0.513E-02
299	PHE	451	N	11739	GLY	192	C	1408	0.678E+00	-0.324E+00	-0.108E-01
300	PHE	451	C	11741	ASN	189	OD1	1387	0.800E+00	-0.318E+00	-0.344E-02
301	PHE	451	C	11741	ASN	189	ND2	1388	0.846E+00	-0.429E+00	-0.289E-02
302	PHE	451	C	11741	LYS	190	O	1392	0.821E+00	-0.325E+00	-0.294E-02
303	PHE	451	C	11741	ASP	191	N	1398	0.750E+00	-0.378E+00	-0.589E-02
304	PHE	451	C	11741	ASP	191	O	1401	0.522E+00	-0.723E+00	-0.423E-01
305	PHE	451	C	11741	ASP	191	OD1	1404	0.873E+00	-0.380E+00	-0.203E-02
306	PHE	451	C	11741	ASP	191	OD2	1405	0.983E+00	-0.316E+00	-0.100E-02
307	PHE	451	C	11741	GLY	192	N	1406	0.502E+00	-0.590E+00	-0.604E-01
308	PHE	451	C	11741	GLY	192	CA	1407	0.440E+00	-0.281E+00	-0.105E+00
309	PHE	451	C	11741	GLY	192	O	1409	0.582E+00	-0.576E+00	-0.227E-01
310	PHE	451	C	11741	PHE	193	N	1410	0.666E+00	-0.317E+00	-0.120E-01
311	PHE	451	C	11741	PRO	223	O	1664	0.699E+00	-0.337E+00	-0.766E-02
312	PHE	451	C	11741	LEU	224	O	1671	0.592E+00	-0.607E+00	-0.204E-01
313	PHE	451	C	11741	GLY	225	N	1676	0.566E+00	-0.449E+00	-0.308E-01
314	PHE	451	C	11741	GLY	225	CA	1677	0.443E+00	-0.277E+00	-0.103E+00
315	PHE	451	C	11741	GLY	225	O	1679	0.460E+00	-0.996E+00	-0.853E-01
316	PHE	451	C	11741	ILE	226	N	1680	0.603E+00	-0.473E+00	-0.215E-01
317	PHE	451	C	11741	ILE	226	O	1683	0.708E+00	-0.469E+00	-0.712E-02
318	PHE	451	O	11742	ASN	189	CG	1386	0.776E+00	-0.345E+00	-0.412E-02
319	PHE	451	O	11742	LYS	190	C	1391	0.751E+00	-0.454E+00	-0.500E-02
320	PHE	451	O	11742	ASP	191	C	1400	0.486E+00	-0.702E+00	-0.634E-01
321	PHE	451	O	11742	ASP	191	CG	1403	0.845E+00	-0.379E+00	-0.247E-02
322	PHE	451	O	11742	GLY	192	N	1406	0.424E+00	0.827E+00	-0.159E+00
323	PHE	451	O	11742	GLY	192	CA	1407	0.340E+00	0.516E+00	-0.216E+00
324	PHE	451	O	11742	GLY	192	C	1408	0.450E+00	-0.111E+01	-0.952E-01
325	PHE	451	O	11742	PHE	193	C	1412	0.758E+00	-0.338E+00	-0.475E-02
326	PHE	451	O	11742	LEU	224	C	1670	0.528E+00	-0.746E+00	-0.398E-01
327	PHE	451	O	11742	GLY	225	N	1676	0.456E+00	0.689E+00	-0.110E+00

328	PHE	451	O	11742	GLY	225	CA	1677	0.335E+00	0.535E+00	-0.194E+00
329	PHE	451	O	11742	GLY	225	C	1678	0.401E+00	-0.149E+01	-0.163E+00
330	PHE	451	O	11742	GLY	225	O	1679	0.398E+00	0.133E+01	-0.192E+00
331	PHE	451	O	11742	ILE	226	C	1682	0.645E+00	-0.478E+00	-0.123E-01
332	PHE	451	CB	11743	ASP	191	C	1400	0.428E+00	-0.187E+00	-0.118E+00
333	PHE	451	CB	11743	ASP	191	O	1401	0.358E+00	0.340E+00	-0.246E+00
334	PHE	451	CB	11743	GLY	192	N	1406	0.449E+00	0.140E+00	-0.122E+00
335	PHE	451	CB	11743	GLY	192	CA	1407	0.411E+00	0.604E-01	-0.154E+00
336	PHE	451	CG	11744	ASP	191	O	1401	0.402E+00	-0.527E-01	-0.161E+00
337	PHE	451	CD2	11746	ASP	191	O	1401	0.354E+00	0.294E+00	-0.217E+00
338	GLU	452	N	11750	GLY	192	C	1408	0.673E+00	-0.374E+00	-0.112E-01
339	GLU	452	N	11750	LEU	224	C	1670	0.682E+00	-0.360E+00	-0.104E-01
340	GLU	452	N	11750	GLY	225	C	1678	0.501E+00	-0.713E+00	-0.615E-01
341	GLU	452	N	11750	GLY	225	O	1679	0.442E+00	0.848E+00	-0.130E+00
342	GLU	452	N	11750	ILE	226	C	1682	0.701E+00	-0.338E+00	-0.884E-02
343	GLU	452	CA	11751	GLY	225	CA	1677	0.453E+00	-0.153E-01	-0.104E+00
344	GLU	452	CA	11751	GLY	225	C	1678	0.418E+00	0.840E-01	-0.129E+00
345	GLU	452	CA	11751	GLY	225	O	1679	0.344E+00	-0.124E+00	-0.232E+00
346	GLU	452	C	11752	GLN	112	NE2	772	0.102E+01	-0.310E+00	-0.940E-03
347	GLU	452	C	11752	LEU	224	O	1671	0.658E+00	-0.418E+00	-0.110E-01
348	GLU	452	C	11752	GLY	225	O	1679	0.388E+00	-0.131E+01	-0.184E+00
349	GLU	452	C	11752	ILE	226	N	1680	0.587E+00	-0.428E+00	-0.250E-01
350	GLU	452	C	11752	ILE	226	O	1683	0.682E+00	-0.433E+00	-0.891E-02
351	GLU	452	C	11752	ASN	227	N	1688	0.662E+00	-0.318E+00	-0.124E-01
352	GLU	452	C	11752	ASN	227	ND2	1695	0.867E+00	-0.353E+00	-0.249E-02
353	GLU	452	O	11753	GLN	112	CD	770	0.101E+01	-0.300E+00	-0.851E-03
354	GLU	452	O	11753	LEU	224	C	1670	0.828E+00	-0.353E+00	-0.279E-02
355	GLU	452	O	11753	GLY	225	C	1678	0.612E+00	-0.637E+00	-0.168E-01
356	GLU	452	O	11753	ILE	226	C	1682	0.755E+00	-0.413E+00	-0.486E-02
357	GLU	452	O	11753	ASN	227	CG	1693	0.886E+00	-0.321E+00	-0.186E-02
358	GLU	452	CB	11754	GLY	225	O	1679	0.416E+00	-0.176E+00	-0.158E+00
359	GLU	452	CG	11755	GLY	225	O	1679	0.395E+00	0.907E-01	-0.194E+00
360	GLU	452	CG	11755	ILE	226	C	1682	0.433E+00	-0.801E-01	-0.113E+00
361	GLU	452	CG	11755	ILE	226	O	1683	0.453E+00	0.780E-01	-0.104E+00
362	GLU	452	CG	11755	ASN	227	N	1688	0.440E+00	0.583E-01	-0.135E+00
363	GLU	452	CD	11756	ASP	85	OD1	561	0.105E+01	-0.402E+00	-0.683E-03
364	GLU	452	CD	11756	SER	105	N	712	0.994E+00	-0.321E+00	-0.110E-02
365	GLU	452	CD	11756	SER	105	OG	717	0.799E+00	-0.541E+00	-0.382E-02
366	GLU	452	CD	11756	GLN	112	O	767	0.955E+00	-0.356E+00	-0.119E-02
367	GLU	452	CD	11756	GLN	112	OE1	771	0.910E+00	-0.426E+00	-0.158E-02
368	GLU	452	CD	11756	ASN	189	OD1	1387	0.780E+00	-0.465E+00	-0.399E-02
369	GLU	452	CD	11756	ASN	189	ND2	1388	0.667E+00	-0.927E+00	-0.118E-01
370	GLU	452	CD	11756	ASP	191	N	1398	0.931E+00	-0.366E+00	-0.162E-02
371	GLU	452	CD	11756	ASP	191	O	1401	0.911E+00	-0.340E+00	-0.157E-02
372	GLU	452	CD	11756	GLY	192	N	1406	0.756E+00	-0.349E+00	-0.564E-02
373	GLU	452	CD	11756	GLY	192	O	1409	0.827E+00	-0.405E+00	-0.282E-02
374	GLU	452	CD	11756	LEU	224	O	1671	0.867E+00	-0.410E+00	-0.212E-02
375	GLU	452	CD	11756	GLY	225	N	1676	0.773E+00	-0.335E+00	-0.493E-02
376	GLU	452	CD	11756	GLY	225	O	1679	0.524E+00	-0.102E+01	-0.414E-01
377	GLU	452	CD	11756	ILE	226	N	1680	0.567E+00	-0.753E+00	-0.306E-01
378	GLU	452	CD	11756	ILE	226	O	1683	0.460E+00	-0.169E+01	-0.849E-01
379	GLU	452	CD	11756	ASN	227	N	1688	0.448E+00	-0.125E+01	-0.109E+00
380	GLU	452	CD	11756	ASN	227	CA	1689	0.424E+00	0.151E+00	-0.123E+00
381	GLU	452	CD	11756	ASN	227	O	1691	0.656E+00	-0.643E+00	-0.112E-01
382	GLU	452	CD	11756	ASN	227	CB	1692	0.442E+00	-0.283E+00	-0.104E+00
383	GLU	452	CD	11756	ASN	227	OD1	1694	0.440E+00	-0.160E+01	-0.106E+00
384	GLU	452	CD	11756	ASN	227	ND2	1695	0.523E+00	-0.157E+01	-0.487E-01
385	GLU	452	CD	11756	ILE	228	N	1696	0.584E+00	-0.706E+00	-0.258E-01
386	GLU	452	CD	11756	ILE	228	O	1699	0.769E+00	-0.561E+00	-0.435E-02
387	GLU	452	CD	11756	THR	229	OG1	1709	0.959E+00	-0.377E+00	-0.128E-02
388	GLU	452	OE1	11757	ASN	189	C	1383	0.995E+00	-0.394E+00	-0.927E-03
389	GLU	452	OE1	11757	ASN	189	CG	1386	0.756E+00	-0.586E+00	-0.479E-02
390	GLU	452	OE1	11757	LYS	190	C	1391	0.951E+00	-0.496E+00	-0.122E-02
391	GLU	452	OE1	11757	ASP	191	C	1400	0.802E+00	-0.401E+00	-0.338E-02
392	GLU	452	OE1	11757	GLY	192	C	1408	0.827E+00	-0.498E+00	-0.280E-02
393	GLU	452	OE1	11757	LEU	224	C	1670	0.930E+00	-0.406E+00	-0.140E-02

394	GLU	452	OE1	11757	GLY	225	C	1678	0.632E+00	-0.828E+00	-0.139E-01
395	GLU	452	OE1	11757	ILE	226	C	1682	0.559E+00	-0.105E+01	-0.284E-01
396	GLU	452	OE1	11757	ASN	227	C	1690	0.641E+00	-0.855E+00	-0.128E-01
397	GLU	452	OE1	11757	ASN	227	CG	1693	0.527E+00	-0.124E+01	-0.399E-01
398	GLU	452	OE1	11757	ILE	228	C	1698	0.821E+00	-0.494E+00	-0.295E-02
399	GLU	452	OE2	11758	GLY	104	C	710	0.943E+00	-0.402E+00	-0.128E-02
400	GLU	452	OE2	11758	SER	105	C	714	0.928E+00	-0.342E+00	-0.141E-02
401	GLU	452	OE2	11758	GLN	112	CD	770	0.952E+00	-0.456E+00	-0.121E-02
402	GLU	452	OE2	11758	ASN	189	CG	1386	0.786E+00	-0.547E+00	-0.382E-02
403	GLU	452	OE2	11758	ASP	191	C	1400	0.949E+00	-0.304E+00	-0.124E-02
404	GLU	452	OE2	11758	GLY	192	C	1408	0.905E+00	-0.429E+00	-0.163E-02
405	GLU	452	OE2	11758	LEU	224	C	1670	0.919E+00	-0.413E+00	-0.150E-02
406	GLU	452	OE2	11758	GLY	225	C	1678	0.598E+00	-0.929E+00	-0.192E-01
407	GLU	452	OE2	11758	ILE	226	C	1682	0.428E+00	-0.201E+01	-0.123E+00
408	GLU	452	OE2	11758	ILE	226	O	1683	0.436E+00	0.208E+01	-0.121E+00
409	GLU	452	OE2	11758	ASN	227	N	1688	0.383E+00	0.202E+01	-0.249E+00
410	GLU	452	OE2	11758	ASN	227	CA	1689	0.330E+00	-0.316E+00	-0.160E+00
411	GLU	452	OE2	11758	ASN	227	C	1690	0.464E+00	-0.178E+01	-0.813E-01
412	GLU	452	OE2	11758	ASN	227	CB	1692	0.328E+00	0.674E+00	-0.136E+00
413	GLU	452	OE2	11758	ASN	227	CG	1693	0.326E+00	-0.426E+01	-0.101E+00
414	GLU	452	OE2	11758	ASN	227	OD1	1694	0.324E+00	0.390E+01	-0.332E+00
415	GLU	452	OE2	11758	ASN	227	ND2	1695	0.401E+00	0.326E+01	-0.208E+00
416	GLU	452	OE2	11758	ILE	228	C	1698	0.687E+00	-0.686E+00	-0.851E-02
417	GLU	452	OE2	11758	THR	229	C	1706	0.968E+00	-0.372E+00	-0.109E-02
418	ARG	453	N	11759	GLY	225	C	1678	0.450E+00	-0.658E+00	-0.107E+00
419	ARG	453	N	11759	GLY	225	O	1679	0.332E+00	0.128E+01	-0.272E+00
420	ARG	453	N	11759	ILE	226	C	1682	0.585E+00	-0.349E+00	-0.255E-01
421	ARG	453	CA	11760	GLY	225	O	1679	0.435E+00	0.274E+00	-0.128E+00
422	ARG	453	C	11761	SER	105	OG	717	0.904E+00	-0.420E+00	-0.183E-02
423	ARG	453	C	11761	LYS	110	O	752	0.976E+00	-0.328E+00	-0.104E-02
424	ARG	453	C	11761	GLN	112	O	767	0.827E+00	-0.429E+00	-0.282E-02
425	ARG	453	C	11761	GLN	112	OE1	771	0.587E+00	-0.927E+00	-0.214E-01
426	ARG	453	C	11761	GLN	112	NE2	772	0.682E+00	-0.956E+00	-0.104E-01
427	ARG	453	C	11761	ASN	129	OD1	897	0.962E+00	-0.313E+00	-0.113E-02
428	ARG	453	C	11761	ASN	129	ND2	898	0.999E+00	-0.440E+00	-0.107E-02
429	ARG	453	C	11761	THR	160	OG1	1142	0.100E+01	-0.336E+00	-0.970E-03
430	ARG	453	C	11761	LEU	224	O	1671	0.755E+00	-0.498E+00	-0.485E-02
431	ARG	453	C	11761	GLY	225	O	1679	0.486E+00	-0.116E+01	-0.634E-01
432	ARG	453	C	11761	ILE	226	N	1680	0.660E+00	-0.522E+00	-0.127E-01
433	ARG	453	C	11761	ILE	226	O	1683	0.758E+00	-0.549E+00	-0.472E-02
434	ARG	453	C	11761	ASN	227	N	1688	0.638E+00	-0.533E+00	-0.155E-01
435	ARG	453	C	11761	ASN	227	O	1691	0.913E+00	-0.338E+00	-0.155E-02
436	ARG	453	C	11761	ASN	227	OD1	1694	0.866E+00	-0.370E+00	-0.213E-02
437	ARG	453	C	11761	ASN	227	ND2	1695	0.807E+00	-0.620E+00	-0.384E-02
438	ARG	453	O	11762	LYS	110	C	751	0.995E+00	-0.325E+00	-0.929E-03
439	ARG	453	O	11762	GLN	112	CD	770	0.527E+00	-0.996E+00	-0.402E-01
440	ARG	453	O	11762	LEU	224	C	1670	0.781E+00	-0.381E+00	-0.396E-02
441	ARG	453	O	11762	GLY	225	C	1678	0.551E+00	-0.781E+00	-0.310E-01
442	ARG	453	O	11762	GLY	225	O	1679	0.441E+00	0.117E+01	-0.113E+00
443	ARG	453	O	11762	ILE	226	C	1682	0.583E+00	-0.676E+00	-0.224E-01
444	ARG	453	O	11762	ASN	227	C	1690	0.779E+00	-0.412E+00	-0.403E-02
445	ARG	453	O	11762	ASN	227	CG	1693	0.735E+00	-0.434E+00	-0.568E-02
446	ARG	453	CB	11763	GLY	225	O	1679	0.436E+00	0.769E-01	-0.126E+00
447	ARG	453	NE	11766	GLN	112	OE1	771	0.792E+00	-0.327E+00	-0.443E-02
448	ARG	453	NE	11766	GLN	112	NE2	772	0.811E+00	-0.441E+00	-0.454E-02
449	ARG	453	NE	11766	ASN	129	ND2	898	0.934E+00	-0.310E+00	-0.196E-02
450	ARG	453	NE	11766	THR	160	OG1	1142	0.686E+00	-0.411E+00	-0.116E-01
451	ARG	453	NE	11766	LEU	224	O	1671	0.558E+00	-0.587E+00	-0.353E-01
452	ARG	453	NE	11766	GLY	225	O	1679	0.555E+00	-0.541E+00	-0.364E-01
453	ARG	453	CZ	11767	GLN	112	OE1	771	0.879E+00	-0.334E+00	-0.195E-02
454	ARG	453	CZ	11767	GLN	112	NE2	772	0.895E+00	-0.456E+00	-0.206E-02
455	ARG	453	CZ	11767	ALA	127	O	883	0.856E+00	-0.308E+00	-0.228E-02
456	ARG	453	CZ	11767	ASN	129	ND2	898	0.969E+00	-0.357E+00	-0.128E-02
457	ARG	453	CZ	11767	ASN	158	O	1126	0.814E+00	-0.317E+00	-0.310E-02
458	ARG	453	CZ	11767	CYS	159	O	1134	0.809E+00	-0.357E+00	-0.320E-02
459	ARG	453	CZ	11767	THR	160	O	1140	0.760E+00	-0.377E+00	-0.466E-02

460	ARG	453	CZ	11767	THR	160	OG1	1142	0.612E+00	-0.630E+00	-0.185E-01
461	ARG	453	CZ	11767	THR	160	CG2	1143	0.483E+00	-0.315E+00	-0.672E-01
462	ARG	453	CZ	11767	PRO	223	O	1664	0.730E+00	-0.320E+00	-0.591E-02
463	ARG	453	CZ	11767	LEU	224	O	1671	0.511E+00	-0.874E+00	-0.477E-01
464	ARG	453	CZ	11767	GLY	225	O	1679	0.594E+00	-0.568E+00	-0.200E-01
465	ARG	453	CZ	11767	ILE	226	N	1680	0.753E+00	-0.313E+00	-0.576E-02
466	ARG	453	NH1	11768	GLN	112	CD	770	0.965E+00	-0.372E+00	-0.131E-02
467	ARG	453	NH1	11768	ALA	127	C	882	0.100E+01	-0.300E+00	-0.106E-02
468	ARG	453	NH1	11768	ASN	158	C	1125	0.970E+00	-0.340E+00	-0.127E-02
469	ARG	453	NH1	11768	CYS	159	C	1133	0.862E+00	-0.428E+00	-0.258E-02
470	ARG	453	NH1	11768	THR	160	C	1139	0.761E+00	-0.463E+00	-0.544E-02
471	ARG	453	NH1	11768	THR	160	CB	1141	0.598E+00	-0.318E+00	-0.255E-01
472	ARG	453	NH1	11768	LEU	224	C	1670	0.677E+00	-0.591E+00	-0.108E-01
473	ARG	453	NH1	11768	GLY	225	C	1678	0.750E+00	-0.493E+00	-0.592E-02
474	ARG	453	NH2	11769	GLN	112	CD	770	0.810E+00	-0.494E+00	-0.374E-02
475	ARG	453	NH2	11769	ALA	127	C	882	0.785E+00	-0.446E+00	-0.451E-02
476	ARG	453	NH2	11769	CYS	128	C	887	0.893E+00	-0.404E+00	-0.208E-02
477	ARG	453	NH2	11769	ASN	129	CG	896	0.964E+00	-0.325E+00	-0.132E-02
478	ARG	453	NH2	11769	ASN	158	C	1125	0.838E+00	-0.431E+00	-0.306E-02
479	ARG	453	NH2	11769	CYS	159	C	1133	0.723E+00	-0.585E+00	-0.737E-02
480	ARG	453	NH2	11769	THR	160	C	1139	0.623E+00	-0.685E+00	-0.177E-01
481	ARG	453	NH2	11769	THR	160	CB	1141	0.423E+00	-0.718E+00	-0.160E+00
482	ARG	453	NH2	11769	THR	160	CG2	1143	0.383E+00	0.685E+00	-0.216E+00
483	ARG	453	NH2	11769	PHE	161	C	1146	0.892E+00	-0.345E+00	-0.211E-02
484	ARG	453	NH2	11769	LEU	224	C	1670	0.568E+00	-0.855E+00	-0.304E-01
485	ARG	453	NH2	11769	LEU	224	O	1671	0.462E+00	0.135E+01	-0.103E+00
486	ARG	453	NH2	11769	GLY	225	C	1678	0.640E+00	-0.672E+00	-0.151E-01
487	ARG	453	NH2	11769	ILE	226	C	1682	0.878E+00	-0.367E+00	-0.230E-02
488	ASP	454	N	11770	GLN	112	CD	770	0.643E+00	-0.623E+00	-0.148E-01
489	ASP	454	N	11770	GLY	225	C	1678	0.732E+00	-0.420E+00	-0.685E-02
490	ASP	454	N	11770	ILE	226	C	1682	0.764E+00	-0.379E+00	-0.528E-02
491	ASP	454	N	11770	ASN	227	CG	1693	0.815E+00	-0.348E+00	-0.361E-02
492	ASP	454	C	11772	GLN	112	OE1	771	0.526E+00	-0.718E+00	-0.403E-01
493	ASP	454	C	11772	GLN	112	NE2	772	0.528E+00	-0.100E+01	-0.459E-01
494	ASP	454	C	11772	ASN	129	ND2	898	0.864E+00	-0.335E+00	-0.254E-02
495	ASP	454	C	11772	ASN	227	ND2	1695	0.906E+00	-0.310E+00	-0.192E-02
496	ASP	454	O	11773	GLN	112	CD	770	0.642E+00	-0.560E+00	-0.126E-01
497	ASP	454	CG	11775	SER	105	OG	717	0.750E+00	-0.590E+00	-0.556E-02
498	ASP	454	CG	11775	LYS	110	O	752	0.924E+00	-0.364E+00	-0.145E-02
499	ASP	454	CG	11775	GLN	112	O	767	0.955E+00	-0.347E+00	-0.119E-02
500	ASP	454	CG	11775	GLN	112	OE1	771	0.623E+00	-0.835E+00	-0.152E-01
501	ASP	454	CG	11775	GLN	112	NE2	772	0.735E+00	-0.846E+00	-0.669E-02
502	ASP	454	CG	11775	GLY	225	O	1679	0.769E+00	-0.448E+00	-0.434E-02
503	ASP	454	CG	11775	ILE	226	N	1680	0.886E+00	-0.312E+00	-0.219E-02
504	ASP	454	CG	11775	ILE	226	O	1683	0.866E+00	-0.444E+00	-0.213E-02
505	ASP	454	CG	11775	ASN	227	N	1688	0.697E+00	-0.455E+00	-0.914E-02
506	ASP	454	CG	11775	ASN	227	O	1691	0.871E+00	-0.372E+00	-0.206E-02
507	ASP	454	CG	11775	ASN	227	OD1	1694	0.732E+00	-0.508E+00	-0.582E-02
508	ASP	454	CG	11775	ASN	227	ND2	1695	0.594E+00	-0.115E+01	-0.233E-01
509	ASP	454	OD1	11776	GLY	104	C	710	0.103E+01	-0.311E+00	-0.737E-03
510	ASP	454	OD1	11776	SER	105	C	714	0.931E+00	-0.302E+00	-0.139E-02
511	ASP	454	OD1	11776	LYS	110	C	751	0.981E+00	-0.419E+00	-0.101E-02
512	ASP	454	OD1	11776	SER	111	C	760	0.933E+00	-0.301E+00	-0.136E-02
513	ASP	454	OD1	11776	GLN	112	CD	770	0.638E+00	-0.827E+00	-0.132E-01
514	ASP	454	OD1	11776	GLY	225	C	1678	0.775E+00	-0.494E+00	-0.413E-02
515	ASP	454	OD1	11776	ILE	226	C	1682	0.664E+00	-0.650E+00	-0.104E-01
516	ASP	454	OD1	11776	ASN	227	C	1690	0.725E+00	-0.594E+00	-0.619E-02
517	ASP	454	OD1	11776	ASN	227	CG	1693	0.537E+00	-0.105E+01	-0.358E-01
518	ASP	454	OD1	11776	ILE	228	C	1698	0.101E+01	-0.314E+00	-0.838E-03
519	ASP	454	OD2	11777	GLN	112	CD	770	0.820E+00	-0.515E+00	-0.296E-02
520	ASP	454	OD2	11777	GLY	225	C	1678	0.943E+00	-0.356E+00	-0.128E-02
521	ASP	454	OD2	11777	ILE	226	C	1682	0.849E+00	-0.413E+00	-0.240E-02
522	ASP	454	OD2	11777	ASN	227	C	1690	0.892E+00	-0.413E+00	-0.179E-02
523	ASP	454	OD2	11777	ASN	227	CG	1693	0.653E+00	-0.691E+00	-0.115E-01
524	ILE	455	N	11778	LYS	110	C	751	0.824E+00	-0.342E+00	-0.336E-02
525	ILE	455	N	11778	GLN	112	CD	770	0.423E+00	-0.132E+01	-0.141E+00

526	ILE	455	N	11778	GLN	112	OE1	771	0.430E+00	0.119E+01	-0.149E+00
527	ILE	455	N	11778	GLN	112	NE2	772	0.400E+00	0.202E+01	-0.226E+00
528	ILE	455	CA	11779	GLN	112	NE2	772	0.389E+00	0.494E+00	-0.211E+00
529	ILE	455	C	11780	LYS	110	O	752	0.630E+00	-0.557E+00	-0.142E-01
530	ILE	455	C	11780	SER	111	N	758	0.845E+00	-0.308E+00	-0.290E-02
531	ILE	455	C	11780	GLN	112	OE1	771	0.511E+00	-0.991E+00	-0.480E-01
532	ILE	455	C	11780	GLN	112	NE2	772	0.377E+00	-0.299E+01	-0.196E+00
533	ILE	455	C	11780	ASN	129	OD1	897	0.683E+00	-0.443E+00	-0.876E-02
534	ILE	455	C	11780	ASN	129	ND2	898	0.654E+00	-0.717E+00	-0.133E-01
535	ILE	455	C	11780	ASN	158	ND2	1130	0.757E+00	-0.542E+00	-0.560E-02
536	ILE	455	O	11781	LYS	110	C	751	0.814E+00	-0.481E+00	-0.309E-02
537	ILE	455	O	11781	GLN	112	CD	770	0.582E+00	-0.854E+00	-0.226E-01
538	ILE	455	O	11781	ASN	129	CG	896	0.778E+00	-0.419E+00	-0.405E-02
539	ILE	455	O	11781	ASN	158	CG	1128	0.785E+00	-0.412E+00	-0.383E-02
540	ILE	455	CB	11782	GLN	112	CG	769	0.429E+00	-0.527E-02	-0.133E+00
541	ILE	455	CB	11782	GLN	112	CD	770	0.392E+00	0.222E+00	-0.154E+00
542	ILE	455	CB	11782	GLN	112	NE2	772	0.337E+00	-0.441E+00	-0.637E-01
543	ILE	455	CG1	11783	GLN	112	CG	769	0.453E+00	-0.163E-02	-0.105E+00
544	ILE	455	CG1	11783	GLN	112	NE2	772	0.438E+00	-0.783E-01	-0.137E+00
545	ILE	455	CG2	11784	GLN	112	NE2	772	0.407E+00	0.556E+00	-0.184E+00
546	ILE	455	CD1	11785	GLN	112	CG	769	0.408E+00	0.978E-02	-0.159E+00
547	ILE	455	CD1	11785	GLN	112	NE2	772	0.460E+00	0.319E+00	-0.109E+00
548	SER	456	N	11786	ASN	109	C	743	0.899E+00	-0.302E+00	-0.200E-02
549	SER	456	N	11786	LYS	110	C	751	0.633E+00	-0.676E+00	-0.161E-01
550	SER	456	N	11786	SER	111	C	760	0.744E+00	-0.328E+00	-0.620E-02
551	SER	456	N	11786	GLN	112	CD	770	0.404E+00	-0.179E+01	-0.169E+00
552	SER	456	N	11786	GLN	112	OE1	771	0.429E+00	0.144E+01	-0.151E+00
553	SER	456	N	11786	ASN	129	CG	896	0.679E+00	-0.472E+00	-0.107E-01
554	SER	456	N	11786	ASN	158	CG	1128	0.848E+00	-0.315E+00	-0.284E-02
555	SER	456	CA	11787	GLN	112	NE2	772	0.359E+00	-0.708E+00	-0.205E+00
556	SER	456	C	11788	LYS	110	O	752	0.573E+00	-0.580E+00	-0.248E-01
557	SER	456	C	11788	GLN	112	OE1	771	0.627E+00	-0.534E+00	-0.146E-01
558	SER	456	C	11788	GLN	112	NE2	772	0.509E+00	-0.119E+01	-0.564E-01
559	SER	456	C	11788	ASN	129	ND2	898	0.783E+00	-0.432E+00	-0.456E-02
560	SER	456	C	11788	ASN	158	ND2	1130	0.873E+00	-0.359E+00	-0.239E-02
561	SER	456	O	11789	LYS	110	C	751	0.735E+00	-0.541E+00	-0.569E-02
562	SER	456	O	11789	GLN	112	CD	770	0.656E+00	-0.622E+00	-0.112E-01
563	SER	456	CB	11790	LYS	110	C	751	0.441E+00	0.421E+00	-0.104E+00
564	SER	456	CB	11790	LYS	110	O	752	0.331E+00	-0.701E+00	-0.165E+00
565	SER	456	CB	11790	LYS	110	CB	753	0.449E+00	-0.600E-01	-0.108E+00
566	SER	456	CB	11790	GLN	112	CD	770	0.430E+00	0.414E+00	-0.116E+00
567	SER	456	CB	11790	GLN	112	OE1	771	0.435E+00	-0.379E+00	-0.128E+00
568	SER	456	CB	11790	GLN	112	NE2	772	0.338E+00	-0.103E+01	-0.805E-01
569	SER	456	OG	11791	THR	106	C	720	0.929E+00	-0.308E+00	-0.154E-02
570	SER	456	OG	11791	ASN	108	C	735	0.843E+00	-0.397E+00	-0.277E-02
571	SER	456	OG	11791	ASN	108	CG	738	0.763E+00	-0.448E+00	-0.503E-02
572	SER	456	OG	11791	ASN	109	C	743	0.659E+00	-0.626E+00	-0.120E-01
573	SER	456	OG	11791	ASN	109	CG	746	0.911E+00	-0.331E+00	-0.174E-02
574	SER	456	OG	11791	LYS	110	CA	750	0.421E+00	0.111E+00	-0.161E+00
575	SER	456	OG	11791	LYS	110	C	751	0.416E+00	-0.212E+01	-0.149E+00
576	SER	456	OG	11791	LYS	110	O	752	0.334E+00	0.298E+01	-0.339E+00
577	SER	456	OG	11791	LYS	110	CB	753	0.349E+00	0.505E+00	-0.230E+00
578	SER	456	OG	11791	LYS	110	CG	754	0.408E+00	-0.102E+00	-0.184E+00
579	SER	456	OG	11791	LYS	110	CD	755	0.413E+00	0.143E+00	-0.174E+00
580	SER	456	OG	11791	SER	111	C	760	0.670E+00	-0.475E+00	-0.109E-01
581	SER	456	OG	11791	GLN	112	CD	770	0.546E+00	-0.102E+01	-0.362E-01
582	SER	456	OG	11791	GLN	112	NE2	772	0.452E+00	0.210E+01	-0.126E+00
583	SER	456	OG	11791	ASN	129	C	893	0.987E+00	-0.310E+00	-0.108E-02
584	SER	456	OG	11791	ASN	129	CG	896	0.692E+00	-0.538E+00	-0.896E-02
585	SER	456	OG	11791	ASN	158	CG	1128	0.969E+00	-0.301E+00	-0.120E-02
586	ASN	457	N	11792	LYS	110	C	751	0.728E+00	-0.407E+00	-0.705E-02
587	ASN	457	N	11792	GLN	112	CD	770	0.710E+00	-0.394E+00	-0.821E-02
588	ASN	457	C	11794	LYS	110	O	752	0.753E+00	-0.427E+00	-0.492E-02
589	ASN	457	C	11794	LYS	110	NZ	757	0.582E+00	-0.319E+00	-0.265E-01
590	ASN	457	C	11794	GLN	112	OE1	771	0.906E+00	-0.347E+00	-0.163E-02
591	ASN	457	C	11794	GLN	112	NE2	772	0.792E+00	-0.612E+00	-0.429E-02

ASN	457	C	11794	ASN	129	ND2	898	0.101E+01	-0.365E+00	-0.986E-03
ASN	457	C	11794	ASN	158	ND2	1130	0.104E+01	-0.352E+00	-0.857E-03
ASN	457	O	11795	LYS	110	C	751	0.826E+00	-0.396E+00	-0.284E-02
ASN	457	CG	11797	GLN	112	NE2	772	0.967E+00	-0.417E+00	-0.129E-02
ASN	457	CG	11797	ASN	158	ND2	1130	0.103E+01	-0.337E+00	-0.896E-03
VAL	458	N	11800	LYS	110	C	751	0.839E+00	-0.331E+00	-0.302E-02
VAL	458	CB	11804	GLN	112	NE2	772	0.875E+00	-0.330E+00	-0.266E-02
PRO	459	CD	11813	LYS	110	NZ	757	0.445E+00	0.116E-01	-0.127E+00
SER	500	OG	12138	ASP	191	CG	1403	0.937E+00	-0.405E+00	-0.147E-02
GLU	502	C	12152	GLU	45	OE1	228	0.987E+00	-0.303E+00	-0.972E-03
GLU	502	O	12153	GLU	45	CD	227	0.981E+00	-0.359E+00	-0.101E-02
GLU	502	CD	12156	GLU	45	OE1	228	0.866E+00	-0.606E+00	-0.213E-02
GLU	502	CD	12156	GLU	45	OE2	229	0.836E+00	-0.644E+00	-0.264E-02
GLU	502	CD	12156	LYS	190	O	1392	0.934E+00	-0.367E+00	-0.136E-02
GLU	502	CD	12156	ASP	191	O	1401	0.957E+00	-0.315E+00	-0.117E-02
GLU	502	CD	12156	ASP	191	OD2	1405	0.986E+00	-0.438E+00	-0.980E-03
GLU	502	CD	12156	PHE	193	CE2	1419	0.399E+00	-0.616E+00	-0.132E+00
GLU	502	CD	12156	PHE	193	CZ	1420	0.430E+00	-0.323E+00	-0.103E+00
GLU	502	CD	12156	LEU	194	O	1424	0.973E+00	-0.342E+00	-0.106E-02
GLU	502	CD	12156	TYR	195	OH	1440	0.678E+00	-0.483E+00	-0.102E-01
GLU	502	CD	12156	PHE	220	O	1636	0.961E+00	-0.317E+00	-0.115E-02
GLU	502	CD	12156	LYS	221	N	1644	0.813E+00	-0.358E+00	-0.367E-02
GLU	502	CD	12156	LYS	221	O	1647	0.712E+00	-0.587E+00	-0.685E-02
GLU	502	CD	12156	LYS	221	NZ	1652	0.512E+00	-0.528E+00	-0.547E-01
GLU	502	CD	12156	LEU	222	O	1656	0.854E+00	-0.420E+00	-0.231E-02
GLU	502	OE1	12157	GLU	45	CD	227	0.947E+00	-0.526E+00	-0.125E-02
GLU	502	OE1	12157	GLY	192	C	1408	0.100E+01	-0.366E+00	-0.880E-03
GLU	502	OE1	12157	PHE	193	C	1412	0.967E+00	-0.365E+00	-0.110E-02
GLU	502	OE1	12157	PHE	193	CE2	1419	0.420E+00	0.581E+00	-0.134E+00
GLU	502	OE1	12157	PHE	193	CZ	1420	0.411E+00	0.389E+00	-0.147E+00
GLU	502	OE1	12157	LEU	194	C	1423	0.104E+01	-0.343E+00	-0.712E-03
GLU	502	OE1	12157	PHE	220	C	1635	0.932E+00	-0.387E+00	-0.138E-02
GLU	502	OE1	12157	LYS	221	C	1646	0.713E+00	-0.813E+00	-0.681E-02
GLU	502	OE1	12157	LEU	222	C	1655	0.790E+00	-0.532E+00	-0.370E-02
GLU	502	OE2	12158	GLU	45	CD	227	0.776E+00	-0.733E+00	-0.411E-02
GLU	502	OE2	12158	LYS	190	C	1391	0.100E+01	-0.458E+00	-0.890E-03
GLU	502	OE2	12158	ASP	191	CG	1403	0.102E+01	-0.455E+00	-0.777E-03
GLU	502	OE2	12158	GLY	192	C	1408	0.998E+00	-0.369E+00	-0.914E-03
GLU	502	OE2	12158	PHE	193	C	1412	0.908E+00	-0.403E+00	-0.161E-02
GLU	502	OE2	12158	PHE	193	CE2	1419	0.396E+00	0.673E+00	-0.171E+00
GLU	502	OE2	12158	LEU	194	C	1423	0.946E+00	-0.395E+00	-0.126E-02
GLU	502	OE2	12158	ILE	219	C	1627	0.101E+01	-0.356E+00	-0.857E-03
GLU	502	OE2	12158	PHE	220	C	1635	0.824E+00	-0.473E+00	-0.287E-02
GLU	502	OE2	12158	LYS	221	C	1646	0.672E+00	-0.914E+00	-0.972E-02
GLU	502	OE2	12158	LYS	221	CD	1650	0.366E+00	0.254E+00	-0.241E+00
GLU	502	OE2	12158	LYS	221	CE	1651	0.443E+00	0.225E+00	-0.116E+00
GLU	502	OE2	12158	LYS	221	NZ	1652	0.395E+00	0.108E+01	-0.221E+00
GLU	502	OE2	12158	LEU	222	C	1655	0.810E+00	-0.509E+00	-0.317E-02
LEU	503	C	12161	GLU	45	OE1	228	0.850E+00	-0.469E+00	-0.238E-02
LEU	503	C	12161	GLU	45	OE2	229	0.971E+00	-0.379E+00	-0.108E-02
LEU	503	O	12162	GLU	45	CD	227	0.873E+00	-0.405E+00	-0.203E-02
LEU	504	C	12169	GLU	45	O	224	0.742E+00	-0.429E+00	-0.539E-02
LEU	504	C	12169	GLU	45	OE1	228	0.560E+00	-0.106E+01	-0.284E-01
LEU	504	C	12169	GLU	45	OE2	229	0.713E+00	-0.642E+00	-0.681E-02
LEU	504	O	12170	ASP	44	CG	218	0.956E+00	-0.342E+00	-0.118E-02
LEU	504	O	12170	GLU	45	C	223	0.680E+00	-0.391E+00	-0.900E-02
LEU	504	O	12170	GLU	45	CD	227	0.608E+00	-0.801E+00	-0.175E-01
ASN	505	N	12175	GLU	45	CD	227	0.589E+00	-0.662E+00	-0.247E-01
ASN	505	CA	12176	GLU	45	OE1	228	0.400E+00	-0.188E+00	-0.185E+00
ASN	505	C	12177	GLU	45	N	221	0.691E+00	-0.378E+00	-0.966E-02
ASN	505	C	12177	GLU	45	O	224	0.693E+00	-0.526E+00	-0.808E-02
ASN	505	C	12177	GLU	45	CD	227	0.385E+00	0.265E+01	-0.139E+00
ASN	505	C	12177	GLU	45	OE1	228	0.321E+00	-0.470E+01	-0.429E-01
ASN	505	C	12177	GLU	45	OE2	229	0.415E+00	-0.235E+01	-0.141E+00
ASN	505	C	12177	ILE	219	O	1628	0.971E+00	-0.307E+00	-0.108E-02
ASN	505	C	12177	LYS	221	NZ	1652	0.532E+00	-0.388E+00	-0.439E-01

ASN	505	O	12178	GLU	45	C	223	0.726E+00	-0.324E+00	-0.611E-02
ASN	505	O	12178	GLU	45	CG	226	0.433E+00	0.738E-01	-0.130E+00
ASN	505	O	12178	GLU	45	OE2	229	0.315E+00	0.419E+01	-0.295E+00
ASN	505	O	12178	LYS	221	C	1646	0.949E+00	-0.316E+00	-0.124E-02
ASN	505	CB	12179	LYS	221	NZ	1652	0.351E+00	0.169E+00	-0.178E+00
ASN	505	CG	12180	ASP	44	N	213	0.818E+00	-0.345E+00	-0.352E-02
ASN	505	CG	12180	ASP	44	O	216	0.824E+00	-0.306E+00	-0.287E-02
ASN	505	CG	12180	ASP	44	OD1	219	0.943E+00	-0.358E+00	-0.128E-02
ASN	505	CG	12180	ASP	44	OD2	220	0.915E+00	-0.375E+00	-0.154E-02
ASN	505	CG	12180	GLU	45	N	221	0.623E+00	-0.441E+00	-0.178E-01
ASN	505	CG	12180	GLU	45	O	224	0.771E+00	-0.408E+00	-0.428E-02
ASN	505	CG	12180	GLU	45	OE1	228	0.557E+00	-0.109E+01	-0.292E-01
ASN	505	CG	12180	GLU	45	OE2	229	0.603E+00	-0.921E+00	-0.184E-01
ASN	505	CG	12180	ASP	191	OD2	1405	0.101E+01	-0.323E+00	-0.846E-03
ASN	505	CG	12180	TYR	195	OH	1440	0.666E+00	-0.382E+00	-0.113E-01
ASN	505	CG	12180	LYS	221	NZ	1652	0.347E+00	-0.109E+01	-0.135E+00
ASN	505	OD1	12181	ASP	44	C	215	0.615E+00	-0.428E+00	-0.164E-01
ASN	505	OD1	12181	ASP	44	CG	218	0.751E+00	-0.485E+00	-0.501E-02
ASN	505	OD1	12181	GLU	45	C	223	0.650E+00	-0.404E+00	-0.118E-01
ASN	505	OD1	12181	GLU	45	CD	227	0.538E+00	-0.991E+00	-0.356E-01
ASN	505	OD1	12181	LYS	221	NZ	1652	0.404E+00	0.653E+00	-0.201E+00
ASN	505	ND2	12182	ASP	44	C	215	0.804E+00	-0.379E+00	-0.391E-02
ASN	505	ND2	12182	ASP	44	CG	218	0.932E+00	-0.498E+00	-0.161E-02
ASN	505	ND2	12182	GLU	45	C	223	0.870E+00	-0.351E+00	-0.243E-02
ASN	505	ND2	12182	GLU	45	CD	227	0.683E+00	-0.886E+00	-0.103E-01
ASN	505	ND2	12182	ASP	191	CG	1403	0.992E+00	-0.454E+00	-0.111E-02
ASN	505	ND2	12182	LYS	221	C	1646	0.101E+01	-0.431E+00	-0.101E-02
ASN	505	ND2	12182	LYS	221	NZ	1652	0.351E+00	0.141E+01	-0.257E+00
ALA	506	N	12183	GLU	45	CD	227	0.456E+00	-0.113E+01	-0.999E-01
ALA	506	N	12183	GLU	45	OE1	228	0.365E+00	0.216E+01	-0.288E+00
ALA	506	CA	12184	GLU	45	OE1	228	0.383E+00	0.131E+00	-0.216E+00
ALA	506	C	12185	GLU	45	O	224	0.791E+00	-0.380E+00	-0.366E-02
ALA	506	C	12185	GLU	45	OE1	228	0.473E+00	-0.156E+01	-0.728E-01
ALA	506	C	12185	GLU	45	OE2	229	0.611E+00	-0.872E+00	-0.170E-01
ALA	506	O	12186	GLU	45	CD	227	0.646E+00	-0.703E+00	-0.123E-01
ALA	506	CB	12187	GLU	45	CD	227	0.561E+00	-0.394E+00	-0.298E-01
PRO	507	C	12190	GLU	45	OE1	228	0.760E+00	-0.332E+00	-0.465E-02
PRO	507	O	12191	GLU	45	CD	227	0.892E+00	-0.305E+00	-0.179E-02
ALA	508	C	12197	GLU	45	OE1	228	0.104E+01	-0.342E+00	-0.717E-03

Table S2 6CRZ-C-RBDtoA-NTD-finedata.txt

TYR	338	C	19214	SER	111	OG	763	0.101E+01	-0.303E+00	-0.957E-03
TYR	338	C	19214	GLN	112	OE1	771	0.930E+00	-0.335E+00	-0.139E-02
TYR	338	C	19214	GLN	112	NE2	772	0.866E+00	-0.529E+00	-0.251E-02
TYR	338	C	19214	ASN	158	ND2	1130	0.930E+00	-0.417E+00	-0.163E-02
TYR	338	O	19215	GLN	112	CD	770	0.816E+00	-0.374E+00	-0.304E-02
ALA	339	C	19226	GLN	112	OE1	771	0.906E+00	-0.320E+00	-0.163E-02
ALA	339	C	19226	GLN	112	NE2	772	0.795E+00	-0.561E+00	-0.419E-02
ALA	339	C	19226	ASN	158	ND2	1130	0.816E+00	-0.474E+00	-0.357E-02
ALA	339	O	19227	GLN	112	CD	770	0.918E+00	-0.325E+00	-0.150E-02
ALA	339	O	19227	ASN	158	CG	1128	0.863E+00	-0.314E+00	-0.218E-02
TRP	340	C	19231	GLN	112	OE1	771	0.934E+00	-0.313E+00	-0.136E-02
TRP	340	C	19231	GLN	112	NE2	772	0.850E+00	-0.512E+00	-0.280E-02
TRP	340	C	19231	ASN	158	ND2	1130	0.919E+00	-0.399E+00	-0.176E-02
TRP	340	O	19232	GLN	112	CD	770	0.824E+00	-0.346E+00	-0.288E-02
GLU	341	C	19245	GLN	112	NE2	772	0.966E+00	-0.336E+00	-0.130E-02
ARG	342	C	19254	CYS	159	O	1134	0.972E+00	-0.342E+00	-0.107E-02
ARG	342	C	19254	THR	160	O	1140	0.838E+00	-0.410E+00	-0.261E-02
ARG	342	C	19254	THR	160	OG1	1142	0.996E+00	-0.340E+00	-0.102E-02
ARG	342	O	19255	THR	160	C	1139	0.836E+00	-0.330E+00	-0.263E-02
ARG	342	NE	19259	GLN	112	NE2	772	0.874E+00	-0.389E+00	-0.292E-02
ARG	342	NE	19259	CYS	159	O	1134	0.686E+00	-0.399E+00	-0.105E-01
ARG	342	NE	19259	THR	160	CA	1138	0.528E+00	-0.323E+00	-0.517E-01
ARG	342	NE	19259	THR	160	O	1140	0.438E+00	-0.104E+01	-0.136E+00

ARG	342	NE	19259	THR	160	OG1	1142	0.623E+00	-0.499E+00	-0.204E-01
ARG	342	NE	19259	PHE	161	N	1144	0.618E+00	-0.313E+00	-0.229E-01
ARG	342	NE	19259	PRO	223	O	1664	0.638E+00	-0.343E+00	-0.160E-01
ARG	342	NE	19259	LEU	224	O	1671	0.686E+00	-0.380E+00	-0.105E-01
ARG	342	CZ	19260	GLN	112	OE1	771	0.809E+00	-0.384E+00	-0.320E-02
ARG	342	CZ	19260	GLN	112	NE2	772	0.840E+00	-0.507E+00	-0.302E-02
ARG	342	CZ	19260	ALA	127	O	883	0.770E+00	-0.370E+00	-0.430E-02
ARG	342	CZ	19260	ASN	158	O	1126	0.824E+00	-0.311E+00	-0.288E-02
ARG	342	CZ	19260	ASN	158	ND2	1130	0.101E+01	-0.334E+00	-0.978E-03
ARG	342	CZ	19260	CYS	159	O	1134	0.587E+00	-0.669E+00	-0.214E-01
ARG	342	CZ	19260	THR	160	N	1137	0.566E+00	-0.304E+00	-0.310E-01
ARG	342	CZ	19260	THR	160	CA	1138	0.424E+00	-0.672E+00	-0.123E+00
ARG	342	CZ	19260	THR	160	C	1139	0.387E+00	0.175E+01	-0.139E+00
ARG	342	CZ	19260	THR	160	CB	1141	0.431E+00	0.566E+00	-0.116E+00
ARG	342	CZ	19260	THR	160	OG1	1142	0.531E+00	-0.862E+00	-0.421E-01
ARG	342	CZ	19260	PHE	161	N	1144	0.490E+00	-0.641E+00	-0.690E-01
ARG	342	CZ	19260	PHE	161	O	1147	0.747E+00	-0.357E+00	-0.514E-02
ARG	342	CZ	19260	GLU	162	N	1155	0.723E+00	-0.317E+00	-0.734E-02
ARG	342	CZ	19260	GLU	162	O	1158	0.887E+00	-0.311E+00	-0.185E-02
ARG	342	CZ	19260	GLU	162	OE1	1162	0.103E+01	-0.341E+00	-0.735E-03
ARG	342	CZ	19260	PRO	223	O	1664	0.570E+00	-0.531E+00	-0.255E-01
ARG	342	CZ	19260	LEU	224	O	1671	0.623E+00	-0.564E+00	-0.152E-01
ARG	342	NH1	19261	GLN	112	CD	770	0.789E+00	-0.518E+00	-0.438E-02
ARG	342	NH1	19261	ARG	126	C	871	0.937E+00	-0.425E+00	-0.157E-02
ARG	342	NH1	19261	ALA	127	C	882	0.780E+00	-0.451E+00	-0.468E-02
ARG	342	NH1	19261	ASN	158	C	1125	0.839E+00	-0.430E+00	-0.304E-02
ARG	342	NH1	19261	ASN	158	CG	1128	0.928E+00	-0.345E+00	-0.165E-02
ARG	342	NH1	19261	CYS	159	C	1133	0.601E+00	-0.847E+00	-0.218E-01
ARG	342	NH1	19261	THR	160	CA	1138	0.374E+00	0.113E+01	-0.221E+00
ARG	342	NH1	19261	THR	160	CB	1141	0.352E+00	-0.117E+01	-0.181E+00
ARG	342	NH1	19261	THR	160	OG1	1142	0.429E+00	0.175E+01	-0.165E+00
ARG	342	NH1	19261	THR	160	CG2	1143	0.457E+00	0.437E+00	-0.112E+00
ARG	342	NH1	19261	PHE	161	N	1144	0.412E+00	0.119E+01	-0.201E+00
ARG	342	NH1	19261	PHE	161	CA	1145	0.454E+00	0.759E-01	-0.116E+00
ARG	342	NH1	19261	PHE	161	C	1146	0.603E+00	-0.717E+00	-0.214E-01
ARG	342	NH1	19261	PHE	161	CB	1148	0.456E+00	0.247E+00	-0.113E+00
ARG	342	NH1	19261	GLY	192	C	1408	0.982E+00	-0.315E+00	-0.118E-02
ARG	342	NH1	19261	LEU	222	C	1655	0.882E+00	-0.367E+00	-0.224E-02
ARG	342	NH1	19261	PRO	223	C	1663	0.580E+00	-0.476E+00	-0.269E-01
ARG	342	NH1	19261	PRO	223	O	1664	0.459E+00	0.107E+01	-0.106E+00
ARG	342	NH1	19261	LEU	224	C	1670	0.586E+00	-0.800E+00	-0.254E-01
ARG	342	NH1	19261	GLY	225	C	1678	0.772E+00	-0.468E+00	-0.499E-02
ARG	342	NH2	19262	GLN	112	CD	770	0.918E+00	-0.402E+00	-0.177E-02
ARG	342	NH2	19262	ARG	126	C	871	0.101E+01	-0.377E+00	-0.975E-03
ARG	342	NH2	19262	ALA	127	C	882	0.872E+00	-0.372E+00	-0.241E-02
ARG	342	NH2	19262	ASN	158	C	1125	0.840E+00	-0.429E+00	-0.301E-02
ARG	342	NH2	19262	ASN	158	CG	1128	0.953E+00	-0.331E+00	-0.142E-02
ARG	342	NH2	19262	CYS	159	C	1133	0.579E+00	-0.918E+00	-0.270E-01
ARG	342	NH2	19262	THR	160	CA	1138	0.415E+00	0.860E+00	-0.173E+00
ARG	342	NH2	19262	THR	160	C	1139	0.361E+00	-0.256E+01	-0.187E+00
ARG	342	NH2	19262	THR	160	CB	1141	0.467E+00	-0.559E+00	-0.999E-01
ARG	342	NH2	19262	PHE	161	N	1144	0.471E+00	0.857E+00	-0.107E+00
ARG	342	NH2	19262	PHE	161	C	1146	0.626E+00	-0.663E+00	-0.172E-01
ARG	342	NH2	19262	GLU	162	C	1157	0.859E+00	-0.314E+00	-0.262E-02
ARG	342	NH2	19262	GLU	162	CD	1161	0.101E+01	-0.397E+00	-0.993E-03
ARG	342	NH2	19262	LEU	224	C	1670	0.803E+00	-0.430E+00	-0.393E-02
ARG	342	NH2	19262	GLY	225	C	1678	0.997E+00	-0.308E+00	-0.108E-02
SER	380	OG	19531	GLU	45	CD	227	0.943E+00	-0.411E+00	-0.142E-02
ASN	381	O	19535	ASP	191	CG	1403	0.986E+00	-0.306E+00	-0.979E-03
ASN	381	CG	19537	ASP	191	OD2	1405	0.938E+00	-0.361E+00	-0.132E-02
ASN	381	CG	19537	LYS	221	O	1647	0.807E+00	-0.357E+00	-0.325E-02
ASN	381	OD1	19538	LYS	221	C	1646	0.902E+00	-0.344E+00	-0.167E-02
ASN	381	ND2	19539	LYS	190	C	1391	0.101E+01	-0.431E+00	-0.101E-02
ASN	381	ND2	19539	ASP	191	C	1400	0.906E+00	-0.310E+00	-0.191E-02
ASN	381	ND2	19539	ASP	191	CG	1403	0.881E+00	-0.545E+00	-0.226E-02
ASN	381	ND2	19539	GLY	192	C	1408	0.955E+00	-0.375E+00	-0.140E-02

ASN	381	ND2	19539	PHE	193	C	1412	0.103E+01	-0.316E+00	-0.894E-03
ASN	381	ND2	19539	PHE	193	CE2	1419	0.436E+00	0.501E+00	-0.125E+00
ASN	381	ND2	19539	PHE	193	CZ	1420	0.382E+00	0.448E+00	-0.193E+00
ASN	381	ND2	19539	LYS	221	C	1646	0.789E+00	-0.640E+00	-0.438E-02
ASN	381	ND2	19539	LEU	222	C	1655	0.834E+00	-0.460E+00	-0.314E-02
TYR	383	OH	19558	PRO	223	CB	1665	0.446E+00	0.487E-02	-0.123E+00
TYR	408	C	19733	ASN	227	ND2	1695	0.101E+01	-0.368E+00	-0.996E-03
LYS	411	C	19765	ASP	191	O	1401	0.894E+00	-0.332E+00	-0.177E-02
LYS	411	C	19765	ASP	191	OD1	1404	0.959E+00	-0.433E+00	-0.116E-02
LYS	411	O	19766	ASP	191	CG	1403	0.974E+00	-0.335E+00	-0.105E-02
LEU	412	C	19774	ASP	191	OD1	1404	0.799E+00	-0.462E+00	-0.346E-02
LEU	412	C	19774	ASP	191	OD2	1405	0.960E+00	-0.342E+00	-0.115E-02
LEU	412	O	19775	ASP	191	CG	1403	0.878E+00	-0.391E+00	-0.197E-02
PRO	413	C	19782	ASP	191	OD1	1404	0.687E+00	-0.356E+00	-0.849E-02
PRO	413	O	19783	ASP	191	CG	1403	0.887E+00	-0.300E+00	-0.185E-02
ASP	414	N	19787	ASP	191	CG	1403	0.716E+00	-0.562E+00	-0.781E-02
ASP	414	C	19789	ASP	191	OD1	1404	0.764E+00	-0.387E+00	-0.451E-02
ASP	414	C	19789	ASP	191	OD2	1405	0.842E+00	-0.326E+00	-0.253E-02
ASP	414	O	19790	ASP	191	CG	1403	0.936E+00	-0.317E+00	-0.134E-02
ASP	414	CG	19792	ASP	191	N	1398	0.102E+01	-0.309E+00	-0.925E-03
ASP	414	CG	19792	ASP	191	OD1	1404	0.759E+00	-0.659E+00	-0.470E-02
ASP	414	CG	19792	ASP	191	OD2	1405	0.917E+00	-0.477E+00	-0.151E-02
ASP	414	OD1	19793	ASP	191	CG	1403	0.939E+00	-0.460E+00	-0.132E-02
ASP	414	OD2	19794	ASP	191	CG	1403	0.858E+00	-0.532E+00	-0.226E-02
ASP	415	N	19795	ASP	191	CG	1403	0.759E+00	-0.503E+00	-0.550E-02
ASP	415	CG	19800	LYS	190	O	1392	0.977E+00	-0.334E+00	-0.103E-02
ASP	415	CG	19800	LYS	190	NZ	1397	0.646E+00	-0.309E+00	-0.144E-01
ASP	415	CG	19800	ASP	191	N	1398	0.870E+00	-0.397E+00	-0.243E-02
ASP	415	CG	19800	ASP	191	O	1401	0.884E+00	-0.347E+00	-0.188E-02
ASP	415	CG	19800	ASP	191	OD1	1404	0.555E+00	-0.125E+01	-0.299E-01
ASP	415	CG	19800	ASP	191	OD2	1405	0.529E+00	-0.139E+01	-0.391E-01
ASP	415	OD1	19801	LYS	190	C	1391	0.957E+00	-0.435E+00	-0.117E-02
ASP	415	OD1	19801	ASP	191	CG	1403	0.585E+00	-0.111E+01	-0.219E-01
ASP	415	OD2	19802	LYS	190	C	1391	0.856E+00	-0.519E+00	-0.228E-02
ASP	415	OD2	19802	ASP	191	C	1400	0.795E+00	-0.360E+00	-0.355E-02
ASP	415	OD2	19802	ASP	191	CG	1403	0.475E+00	-0.179E+01	-0.714E-01
ASP	415	OD2	19802	ASP	191	OD2	1405	0.431E+00	0.224E+01	-0.129E+00
ARG	441	CZ	20014	SER	111	OG	763	0.870E+00	-0.346E+00	-0.229E-02
ARG	441	CZ	20014	ASN	227	ND2	1695	0.101E+01	-0.335E+00	-0.993E-03
ARG	441	NH2	20016	LYS	110	C	751	0.952E+00	-0.412E+00	-0.143E-02
LEU	443	C	20031	ASN	227	ND2	1695	0.992E+00	-0.348E+00	-0.111E-02
ARG	444	C	20039	SER	105	OG	717	0.968E+00	-0.377E+00	-0.121E-02
ARG	444	C	20039	ASN	227	OD1	1694	0.896E+00	-0.350E+00	-0.174E-02
ARG	444	C	20039	ASN	227	ND2	1695	0.679E+00	-0.854E+00	-0.107E-01
ARG	444	O	20040	ASN	227	CG	1693	0.845E+00	-0.338E+00	-0.247E-02
ARG	444	NE	20044	SER	105	OG	717	0.816E+00	-0.317E+00	-0.412E-02
ARG	444	NE	20044	SER	111	OG	763	0.819E+00	-0.315E+00	-0.402E-02
ARG	444	NE	20044	ASN	227	OD1	1694	0.669E+00	-0.378E+00	-0.122E-01
ARG	444	NE	20044	ASN	227	ND2	1695	0.492E+00	-0.110E+01	-0.845E-01
ARG	444	CZ	20045	SER	105	OG	717	0.750E+00	-0.448E+00	-0.557E-02
ARG	444	CZ	20045	SER	111	OG	763	0.805E+00	-0.395E+00	-0.364E-02
ARG	444	CZ	20045	GLY	225	O	1679	0.769E+00	-0.341E+00	-0.435E-02
ARG	444	CZ	20045	ILE	226	O	1683	0.729E+00	-0.457E+00	-0.595E-02
ARG	444	CZ	20045	ASN	227	N	1688	0.572E+00	-0.520E+00	-0.290E-01
ARG	444	CZ	20045	ASN	227	O	1691	0.686E+00	-0.435E+00	-0.860E-02
ARG	444	CZ	20045	ASN	227	CB	1692	0.416E+00	-0.245E+00	-0.132E+00
ARG	444	CZ	20045	ASN	227	CG	1693	0.432E+00	0.138E+01	-0.101E+00
ARG	444	CZ	20045	ASN	227	OD1	1694	0.537E+00	-0.735E+00	-0.358E-01
ARG	444	CZ	20045	ASN	227	ND2	1695	0.372E+00	-0.273E+01	-0.196E+00
ARG	444	NH1	20046	GLY	225	C	1678	0.900E+00	-0.361E+00	-0.200E-02
ARG	444	NH1	20046	ILE	226	C	1682	0.706E+00	-0.542E+00	-0.849E-02
ARG	444	NH1	20046	ASN	227	C	1690	0.663E+00	-0.664E+00	-0.123E-01
ARG	444	NH1	20046	ASN	227	CB	1692	0.416E+00	0.296E+00	-0.170E+00
ARG	444	NH1	20046	ASN	227	CG	1693	0.385E+00	-0.225E+01	-0.191E+00
ARG	444	NH1	20046	ILE	228	C	1698	0.879E+00	-0.366E+00	-0.229E-02
ARG	444	NH2	20047	GLY	104	C	710	0.926E+00	-0.344E+00	-0.168E-02

856	ARG	444	NH2	20047	GLN	112	CD	770	0.952E+00	-0.379E+00	-0.142E-02
857	ARG	444	NH2	20047	GLY	225	C	1678	0.718E+00	-0.535E+00	-0.765E-02
858	ARG	444	NH2	20047	ILE	226	C	1682	0.548E+00	-0.921E+00	-0.373E-01
859	ARG	444	NH2	20047	ASN	227	N	1688	0.452E+00	0.110E+01	-0.132E+00
860	ARG	444	NH2	20047	ASN	227	CA	1689	0.444E+00	-0.120E+00	-0.128E+00
861	ARG	444	NH2	20047	ASN	227	C	1690	0.572E+00	-0.907E+00	-0.291E-01
862	ARG	444	NH2	20047	ASN	227	CG	1693	0.389E+00	-0.219E+01	-0.187E+00
863	ARG	444	NH2	20047	ASN	227	ND2	1695	0.366E+00	0.344E+01	-0.276E+00
864	ARG	444	NH2	20047	ILE	228	C	1698	0.847E+00	-0.390E+00	-0.287E-02
865	HIS	445	N	20048	ASN	227	CG	1693	0.739E+00	-0.392E+00	-0.645E-02
866	HIS	445	C	20050	SER	105	O	715	0.956E+00	-0.316E+00	-0.118E-02
867	HIS	445	C	20050	SER	105	OG	717	0.780E+00	-0.488E+00	-0.441E-02
868	HIS	445	C	20050	ASN	227	O	1691	0.820E+00	-0.366E+00	-0.296E-02
869	HIS	445	C	20050	ASN	227	OD1	1694	0.638E+00	-0.592E+00	-0.131E-01
870	HIS	445	C	20050	ASN	227	ND2	1695	0.436E+00	-0.211E+01	-0.124E+00
871	HIS	445	C	20050	ILE	228	O	1699	0.966E+00	-0.332E+00	-0.111E-02
872	HIS	445	C	20050	THR	229	OG1	1709	0.782E+00	-0.458E+00	-0.435E-02
873	HIS	445	O	20051	ASN	227	C	1690	0.721E+00	-0.435E+00	-0.640E-02
874	HIS	445	O	20051	ASN	227	CG	1693	0.451E+00	-0.116E+01	-0.943E-01
875	HIS	445	O	20051	ASN	227	ND2	1695	0.321E+00	0.383E+01	-0.176E+00
876	HIS	445	O	20051	ILE	228	C	1698	0.837E+00	-0.306E+00	-0.261E-02
877	HIS	445	CG	20053	ASN	227	ND2	1695	0.704E+00	-0.303E+00	-0.863E-02
878	GLY	446	N	20058	ASN	227	CG	1693	0.618E+00	-0.396E+00	-0.186E-01
879	GLY	446	CA	20059	ASN	227	ND2	1695	0.439E+00	0.404E+00	-0.136E+00
880	GLY	446	C	20060	ASN	227	OD1	1694	0.622E+00	-0.549E+00	-0.154E-01
881	GLY	446	C	20060	ASN	227	ND2	1695	0.468E+00	-0.155E+01	-0.883E-01
882	GLY	446	O	20061	ASN	227	CG	1693	0.596E+00	-0.583E+00	-0.197E-01
883	LYS	447	N	20062	ASN	227	CG	1693	0.622E+00	-0.456E+00	-0.179E-01
884	LYS	447	C	20064	ASN	189	ND2	1388	0.104E+01	-0.412E+00	-0.840E-03
885	LYS	447	C	20064	ILE	226	O	1683	0.912E+00	-0.398E+00	-0.156E-02
886	LYS	447	C	20064	ASN	227	N	1688	0.853E+00	-0.308E+00	-0.274E-02
887	LYS	447	C	20064	ASN	227	O	1691	0.949E+00	-0.316E+00	-0.123E-02
888	LYS	447	C	20064	ASN	227	OD1	1694	0.624E+00	-0.678E+00	-0.150E-01
889	LYS	447	C	20064	ASN	227	ND2	1695	0.546E+00	-0.135E+01	-0.381E-01
890	LYS	447	C	20064	ILE	228	O	1699	0.101E+01	-0.340E+00	-0.845E-03
891	LYS	447	O	20065	ILE	226	C	1682	0.825E+00	-0.335E+00	-0.285E-02
892	LYS	447	O	20065	ASN	227	C	1690	0.784E+00	-0.397E+00	-0.388E-02
893	LYS	447	O	20065	ASN	227	CG	1693	0.504E+00	-0.942E+00	-0.518E-01
894	LYS	447	O	20065	ASN	227	ND2	1695	0.464E+00	0.154E+01	-0.101E+00
895	LEU	448	N	20071	ASN	227	CG	1693	0.671E+00	-0.318E+00	-0.115E-01
896	LEU	448	C	20073	ASN	189	ND2	1388	0.103E+01	-0.332E+00	-0.911E-03
897	LEU	448	C	20073	ASP	191	N	1398	0.858E+00	-0.313E+00	-0.266E-02
898	LEU	448	C	20073	ASP	191	OD1	1404	0.935E+00	-0.356E+00	-0.135E-02
899	LEU	448	C	20073	ILE	226	O	1683	0.832E+00	-0.366E+00	-0.271E-02
900	LEU	448	C	20073	ASN	227	OD1	1694	0.723E+00	-0.400E+00	-0.629E-02
901	LEU	448	C	20073	ASN	227	ND2	1695	0.711E+00	-0.614E+00	-0.815E-02
902	LEU	448	O	20074	LYS	190	C	1391	0.974E+00	-0.324E+00	-0.106E-02
903	LEU	448	O	20074	ASP	191	CG	1403	0.993E+00	-0.323E+00	-0.942E-03
904	LEU	448	O	20074	ASN	227	CG	1693	0.819E+00	-0.344E+00	-0.297E-02
905	ARG	449	C	20081	ASN	189	O	1384	0.944E+00	-0.321E+00	-0.127E-02
906	ARG	449	C	20081	ASN	189	ND2	1388	0.103E+01	-0.423E+00	-0.913E-03
907	ARG	449	C	20081	LYS	190	N	1389	0.850E+00	-0.316E+00	-0.280E-02
908	ARG	449	C	20081	LYS	190	O	1392	0.796E+00	-0.457E+00	-0.354E-02
909	ARG	449	C	20081	ASP	191	N	1398	0.642E+00	-0.682E+00	-0.148E-01
910	ARG	449	C	20081	ASP	191	O	1401	0.569E+00	-0.793E+00	-0.258E-01
911	ARG	449	C	20081	ASP	191	OD1	1404	0.709E+00	-0.734E+00	-0.707E-02
912	ARG	449	C	20081	ASP	191	OD2	1405	0.888E+00	-0.493E+00	-0.184E-02
913	ARG	449	C	20081	GLY	192	N	1406	0.489E+00	-0.837E+00	-0.696E-01
914	ARG	449	C	20081	GLY	192	O	1409	0.721E+00	-0.495E+00	-0.639E-02
915	ARG	449	C	20081	PHE	193	N	1410	0.746E+00	-0.339E+00	-0.612E-02
916	ARG	449	C	20081	PHE	193	O	1413	0.890E+00	-0.341E+00	-0.182E-02
917	ARG	449	C	20081	LEU	224	O	1671	0.102E+01	-0.305E+00	-0.804E-03
918	ARG	449	C	20081	GLY	225	O	1679	0.735E+00	-0.477E+00	-0.566E-02
919	ARG	449	C	20081	ILE	226	N	1680	0.827E+00	-0.343E+00	-0.331E-02
920	ARG	449	C	20081	ILE	226	O	1683	0.773E+00	-0.530E+00	-0.421E-02
921	ARG	449	C	20081	ASN	227	N	1688	0.844E+00	-0.316E+00	-0.293E-02

922	ARG	449	C	20081	ASN	227	OD1	1694	0.855E+00	-0.378E+00	-0.230E-02
923	ARG	449	C	20081	ASN	227	ND2	1695	0.899E+00	-0.517E+00	-0.200E-02
924	ARG	449	O	20082	LYS	190	C	1391	0.839E+00	-0.425E+00	-0.257E-02
925	ARG	449	O	20082	ASP	191	C	1400	0.605E+00	-0.486E+00	-0.180E-01
926	ARG	449	O	20082	ASP	191	CG	1403	0.866E+00	-0.414E+00	-0.213E-02
927	ARG	449	O	20082	GLY	192	C	1408	0.696E+00	-0.479E+00	-0.788E-02
928	ARG	449	O	20082	GLY	225	C	1678	0.705E+00	-0.467E+00	-0.730E-02
929	ARG	449	O	20082	ILE	226	C	1682	0.770E+00	-0.388E+00	-0.431E-02
930	ARG	449	O	20082	ASN	227	CG	1693	0.833E+00	-0.347E+00	-0.270E-02
931	ARG	449	CB	20083	GLY	192	N	1406	0.390E+00	0.751E-01	-0.209E+00
932	ARG	449	CB	20083	GLY	192	CA	1407	0.429E+00	0.202E-01	-0.132E+00
933	ARG	449	CG	20084	GLY	192	N	1406	0.455E+00	-0.165E-01	-0.115E+00
934	ARG	449	CD	20085	ASN	189	CB	1385	0.418E+00	-0.537E-01	-0.146E+00
935	ARG	449	CD	20085	LYS	190	N	1389	0.460E+00	-0.196E+00	-0.108E+00
936	ARG	449	CD	20085	ASP	191	N	1398	0.459E+00	-0.251E+00	-0.109E+00
937	ARG	449	CD	20085	GLY	192	N	1406	0.405E+00	-0.231E+00	-0.188E+00
938	ARG	449	CD	20085	GLY	192	CA	1407	0.457E+00	-0.589E-01	-0.100E+00
939	ARG	449	NE	20086	LYS	84	O	549	0.742E+00	-0.331E+00	-0.657E-02
940	ARG	449	NE	20086	ASP	85	O	558	0.636E+00	-0.398E+00	-0.164E-01
941	ARG	449	NE	20086	ASP	85	OD1	561	0.779E+00	-0.392E+00	-0.491E-02
942	ARG	449	NE	20086	LYS	188	O	1375	0.716E+00	-0.353E+00	-0.807E-02
943	ARG	449	NE	20086	ASN	189	N	1381	0.555E+00	-0.458E+00	-0.428E-01
944	ARG	449	NE	20086	ASN	189	CA	1382	0.441E+00	0.829E-01	-0.133E+00
945	ARG	449	NE	20086	ASN	189	C	1383	0.412E+00	0.135E+01	-0.158E+00
946	ARG	449	NE	20086	ASN	189	O	1384	0.413E+00	-0.114E+01	-0.182E+00
947	ARG	449	NE	20086	ASN	189	CB	1385	0.344E+00	-0.333E+00	-0.132E+00
948	ARG	449	NE	20086	ASN	189	CG	1386	0.444E+00	0.105E+01	-0.114E+00
949	ARG	449	NE	20086	ASN	189	OD1	1387	0.531E+00	-0.620E+00	-0.470E-01
950	ARG	449	NE	20086	ASN	189	ND2	1388	0.475E+00	-0.120E+01	-0.102E+00
951	ARG	449	NE	20086	LYS	190	N	1389	0.448E+00	-0.772E+00	-0.138E+00
952	ARG	449	NE	20086	LYS	190	O	1392	0.630E+00	-0.455E+00	-0.173E-01
953	ARG	449	NE	20086	ASP	191	N	1398	0.519E+00	-0.691E+00	-0.622E-01
954	ARG	449	NE	20086	ASP	191	OD1	1404	0.765E+00	-0.405E+00	-0.546E-02
955	ARG	449	NE	20086	ASP	191	OD2	1405	0.900E+00	-0.307E+00	-0.207E-02
956	ARG	449	NE	20086	GLY	192	N	1406	0.523E+00	-0.457E+00	-0.601E-01
957	ARG	449	NE	20086	PHE	193	O	1413	0.597E+00	-0.460E+00	-0.238E-01
958	ARG	449	NE	20086	ILE	226	O	1683	0.675E+00	-0.437E+00	-0.115E-01
959	ARG	449	NE	20086	ASN	227	OD1	1694	0.620E+00	-0.442E+00	-0.191E-01
960	ARG	449	NE	20086	ASN	227	ND2	1695	0.790E+00	-0.410E+00	-0.534E-02
961	ARG	449	NE	20086	ILE	228	O	1699	0.707E+00	-0.398E+00	-0.872E-02
962	ARG	449	CZ	20087	ASP	57	OD1	331	0.965E+00	-0.335E+00	-0.112E-02
963	ARG	449	CZ	20087	LYS	84	O	549	0.742E+00	-0.402E+00	-0.536E-02
964	ARG	449	CZ	20087	ASP	85	N	555	0.785E+00	-0.360E+00	-0.451E-02
965	ARG	449	CZ	20087	ASP	85	O	558	0.595E+00	-0.558E+00	-0.199E-01
966	ARG	449	CZ	20087	ASP	85	OD1	561	0.731E+00	-0.537E+00	-0.590E-02
967	ARG	449	CZ	20087	ASP	85	OD2	562	0.926E+00	-0.357E+00	-0.143E-02
968	ARG	449	CZ	20087	LYS	188	O	1375	0.691E+00	-0.461E+00	-0.823E-02
969	ARG	449	CZ	20087	ASN	189	N	1381	0.522E+00	-0.640E+00	-0.488E-01
970	ARG	449	CZ	20087	ASN	189	CA	1382	0.424E+00	0.111E+00	-0.123E+00
971	ARG	449	CZ	20087	ASN	189	C	1383	0.361E+00	0.234E+01	-0.117E+00
972	ARG	449	CZ	20087	ASN	189	O	1384	0.327E+00	-0.260E+01	-0.112E+00
973	ARG	449	CZ	20087	ASN	189	CB	1385	0.357E+00	-0.365E+00	-0.121E+00
974	ARG	449	CZ	20087	ASN	189	OD1	1387	0.579E+00	-0.622E+00	-0.233E-01
975	ARG	449	CZ	20087	ASN	189	ND2	1388	0.518E+00	-0.119E+01	-0.510E-01
976	ARG	449	CZ	20087	LYS	190	N	1389	0.409E+00	-0.118E+01	-0.161E+00
977	ARG	449	CZ	20087	LYS	190	CA	1390	0.435E+00	-0.906E-01	-0.111E+00
978	ARG	449	CZ	20087	LYS	190	O	1392	0.610E+00	-0.595E+00	-0.172E-01
979	ARG	449	CZ	20087	ASP	191	N	1398	0.489E+00	-0.970E+00	-0.700E-01
980	ARG	449	CZ	20087	ASP	191	O	1401	0.762E+00	-0.340E+00	-0.457E-02
981	ARG	449	CZ	20087	ASP	191	OD1	1404	0.718E+00	-0.556E+00	-0.656E-02
982	ARG	449	CZ	20087	ASP	191	OD2	1405	0.846E+00	-0.414E+00	-0.246E-02
983	ARG	449	CZ	20087	GLY	192	N	1406	0.562E+00	-0.471E+00	-0.321E-01
984	ARG	449	CZ	20087	GLY	192	O	1409	0.808E+00	-0.312E+00	-0.323E-02
985	ARG	449	CZ	20087	PHE	193	O	1413	0.620E+00	-0.516E+00	-0.155E-01
986	ARG	449	CZ	20087	ILE	226	O	1683	0.796E+00	-0.390E+00	-0.353E-02
987	ARG	449	CZ	20087	ASN	227	OD1	1694	0.738E+00	-0.381E+00	-0.557E-02

988	ARG	449	CZ	20087	ASN	227	ND2	1695	0.908E+00	-0.395E+00	-0.189E-02
989	ARG	449	CZ	20087	ILE	228	O	1699	0.788E+00	-0.397E+00	-0.375E-02
990	ARG	449	NH1	20088	ASP	57	CG	330	0.104E+01	-0.372E+00	-0.842E-03
991	ARG	449	NH1	20088	LYS	84	C	548	0.927E+00	-0.429E+00	-0.166E-02
992	ARG	449	NH1	20088	ASP	85	C	557	0.769E+00	-0.359E+00	-0.509E-02
993	ARG	449	NH1	20088	ASP	85	CG	560	0.899E+00	-0.463E+00	-0.201E-02
994	ARG	449	NH1	20088	LYS	188	C	1374	0.671E+00	-0.763E+00	-0.115E-01
995	ARG	449	NH1	20088	ASN	189	CA	1382	0.461E+00	-0.109E+00	-0.107E+00
996	ARG	449	NH1	20088	ASN	189	C	1383	0.357E+00	-0.290E+01	-0.179E+00
997	ARG	449	NH1	20088	ASN	189	O	1384	0.320E+00	0.335E+01	-0.166E+00
998	ARG	449	NH1	20088	ASN	189	CB	1385	0.433E+00	0.266E+00	-0.144E+00
999	ARG	449	NH1	20088	ASN	189	CG	1386	0.573E+00	-0.854E+00	-0.287E-01
1000	ARG	449	NH1	20088	LYS	190	N	1389	0.372E+00	0.184E+01	-0.273E+00
1001	ARG	449	NH1	20088	LYS	190	CA	1390	0.354E+00	0.189E+00	-0.189E+00
1002	ARG	449	NH1	20088	LYS	190	C	1391	0.415E+00	-0.230E+01	-0.153E+00
1003	ARG	449	NH1	20088	ASP	191	N	1398	0.382E+00	0.219E+01	-0.260E+00
1004	ARG	449	NH1	20088	ASP	191	C	1400	0.561E+00	-0.680E+00	-0.326E-01
1005	ARG	449	NH1	20088	ASP	191	CG	1403	0.634E+00	-0.880E+00	-0.161E-01
1006	ARG	449	NH1	20088	GLY	192	C	1408	0.684E+00	-0.587E+00	-0.102E-01
1007	ARG	449	NH1	20088	PHE	193	C	1412	0.723E+00	-0.498E+00	-0.733E-02
1008	ARG	449	NH1	20088	LEU	194	C	1423	0.932E+00	-0.336E+00	-0.161E-02
1009	ARG	449	NH1	20088	ILE	226	C	1682	0.968E+00	-0.315E+00	-0.129E-02
1010	ARG	449	NH1	20088	ASN	227	C	1690	0.995E+00	-0.328E+00	-0.109E-02
1011	ARG	449	NH1	20088	ASN	227	CG	1693	0.939E+00	-0.339E+00	-0.155E-02
1012	ARG	449	NH2	20089	ASP	57	CG	330	0.103E+01	-0.378E+00	-0.900E-03
1013	ARG	449	NH2	20089	PHE	83	C	537	0.959E+00	-0.308E+00	-0.136E-02
1014	ARG	449	NH2	20089	LYS	84	C	548	0.704E+00	-0.693E+00	-0.861E-02
1015	ARG	449	NH2	20089	ASP	85	C	557	0.573E+00	-0.648E+00	-0.288E-01
1016	ARG	449	NH2	20089	ASP	85	CG	560	0.676E+00	-0.771E+00	-0.109E-01
1017	ARG	449	NH2	20089	GLY	86	C	565	0.893E+00	-0.365E+00	-0.208E-02
1018	ARG	449	NH2	20089	PHE	187	C	1363	0.910E+00	-0.334E+00	-0.186E-02
1019	ARG	449	NH2	20089	LYS	188	C	1374	0.622E+00	-0.889E+00	-0.179E-01
1020	ARG	449	NH2	20089	ASN	189	CA	1382	0.431E+00	-0.129E+00	-0.147E+00
1021	ARG	449	NH2	20089	ASN	189	C	1383	0.382E+00	-0.243E+01	-0.193E+00
1022	ARG	449	NH2	20089	ASN	189	O	1384	0.317E+00	0.343E+01	-0.131E+00
1023	ARG	449	NH2	20089	ASN	189	CB	1385	0.360E+00	0.433E+00	-0.209E+00
1024	ARG	449	NH2	20089	ASN	189	CG	1386	0.481E+00	-0.128E+01	-0.767E-01
1025	ARG	449	NH2	20089	LYS	190	N	1389	0.465E+00	0.104E+01	-0.114E+00
1026	ARG	449	NH2	20089	LYS	190	C	1391	0.597E+00	-0.969E+00	-0.227E-01
1027	ARG	449	NH2	20089	ASP	191	C	1400	0.764E+00	-0.364E+00	-0.531E-02
1028	ARG	449	NH2	20089	ASP	191	CG	1403	0.856E+00	-0.502E+00	-0.269E-02
1029	ARG	449	NH2	20089	GLY	192	C	1408	0.824E+00	-0.417E+00	-0.338E-02
1030	ARG	449	NH2	20089	PHE	193	C	1412	0.809E+00	-0.406E+00	-0.377E-02
1031	ARG	449	NH2	20089	LEU	194	C	1423	0.968E+00	-0.317E+00	-0.129E-02
1032	ARG	449	NH2	20089	ILE	226	C	1682	0.973E+00	-0.312E+00	-0.125E-02
1033	ARG	449	NH2	20089	ASN	227	C	1690	0.927E+00	-0.365E+00	-0.167E-02
1034	ARG	449	NH2	20089	ASN	227	CG	1693	0.868E+00	-0.384E+00	-0.247E-02
1035	ARG	449	NH2	20089	ILE	228	C	1698	0.871E+00	-0.372E+00	-0.242E-02
1036	ARG	449	NH2	20089	LYS	258	C	1908	0.104E+01	-0.362E+00	-0.843E-03
1037	PRO	450	N	20090	GLY	192	N	1406	0.459E+00	0.118E+00	-0.121E+00
1038	PRO	450	C	20092	ASP	191	O	1401	0.421E+00	-0.744E+00	-0.132E+00
1039	PRO	450	C	20092	ASP	191	OD1	1404	0.694E+00	-0.349E+00	-0.797E-02
1040	PRO	450	C	20092	GLY	192	N	1406	0.462E+00	-0.441E+00	-0.943E-01
1041	PRO	450	C	20092	GLY	192	O	1409	0.614E+00	-0.312E+00	-0.165E-01
1042	PRO	450	O	20093	LYS	190	C	1391	0.648E+00	-0.519E+00	-0.120E-01
1043	PRO	450	O	20093	ASP	191	C	1400	0.351E+00	-0.139E+01	-0.216E+00
1044	PRO	450	O	20093	ASP	191	O	1401	0.323E+00	0.198E+01	-0.329E+00
1045	PRO	450	O	20093	ASP	191	CG	1403	0.655E+00	-0.522E+00	-0.113E-01
1046	PRO	450	O	20093	GLY	192	N	1406	0.349E+00	0.119E+01	-0.307E+00
1047	PRO	450	O	20093	GLY	192	CA	1407	0.330E+00	0.479E+00	-0.157E+00
1048	PRO	450	O	20093	GLY	192	C	1408	0.452E+00	-0.938E+00	-0.925E-01
1049	PRO	450	O	20093	LEU	224	C	1670	0.760E+00	-0.301E+00	-0.466E-02
1050	PRO	450	O	20093	GLY	225	C	1678	0.635E+00	-0.434E+00	-0.136E-01
1051	PRO	450	CG	20095	ASP	191	CA	1399	0.386E+00	0.509E-03	-0.177E+00
1052	PRO	450	CG	20095	ASP	191	C	1400	0.398E+00	0.297E-01	-0.150E+00
1053	PRO	450	CG	20095	ASP	191	O	1401	0.411E+00	-0.308E-01	-0.166E+00

1054	PRO	450	CG	20095	ASP	191	CB	1402	0.440E+00	-0.248E-02	-0.119E+00
1055	PRO	450	CG	20095	ASP	191	OD1	1404	0.417E+00	-0.433E-01	-0.156E+00
1056	PRO	450	CG	20095	GLY	192	N	1406	0.457E+00	-0.176E-01	-0.112E+00
1057	PRO	450	CD	20096	ASP	191	CA	1399	0.424E+00	-0.367E-03	-0.139E+00
1058	PRO	450	CD	20096	ASP	191	C	1400	0.434E+00	-0.219E-01	-0.112E+00
1059	PRO	450	CD	20096	GLY	192	N	1406	0.453E+00	0.166E-01	-0.117E+00
1060	PHE	451	N	20097	ASP	191	C	1400	0.566E+00	-0.360E+00	-0.309E-01
1061	PHE	451	N	20097	GLY	192	C	1408	0.653E+00	-0.349E+00	-0.135E-01
1062	PHE	451	N	20097	GLY	225	C	1678	0.699E+00	-0.305E+00	-0.902E-02
1063	PHE	451	C	20099	GLN	112	OE1	771	0.803E+00	-0.377E+00	-0.336E-02
1064	PHE	451	C	20099	GLN	112	NE2	772	0.948E+00	-0.403E+00	-0.145E-02
1065	PHE	451	C	20099	THR	160	OG1	1142	0.868E+00	-0.317E+00	-0.233E-02
1066	PHE	451	C	20099	ASP	191	O	1401	0.585E+00	-0.559E+00	-0.219E-01
1067	PHE	451	C	20099	ASP	191	OD1	1404	0.965E+00	-0.325E+00	-0.112E-02
1068	PHE	451	C	20099	GLY	192	N	1406	0.596E+00	-0.401E+00	-0.228E-01
1069	PHE	451	C	20099	GLY	192	O	1409	0.566E+00	-0.610E+00	-0.264E-01
1070	PHE	451	C	20099	LEU	224	O	1671	0.629E+00	-0.535E+00	-0.144E-01
1071	PHE	451	C	20099	GLY	225	N	1676	0.562E+00	-0.457E+00	-0.323E-01
1072	PHE	451	C	20099	GLY	225	CA	1677	0.433E+00	-0.292E+00	-0.113E+00
1073	PHE	451	C	20099	GLY	225	O	1679	0.418E+00	-0.127E+01	-0.137E+00
1074	PHE	451	C	20099	ILE	226	N	1680	0.589E+00	-0.496E+00	-0.245E-01
1075	PHE	451	C	20099	ILE	226	O	1683	0.705E+00	-0.473E+00	-0.729E-02
1076	PHE	451	O	20100	GLN	112	CD	770	0.843E+00	-0.341E+00	-0.250E-02
1077	PHE	451	O	20100	LYS	190	C	1391	0.897E+00	-0.334E+00	-0.173E-02
1078	PHE	451	O	20100	ASP	191	C	1400	0.628E+00	-0.394E+00	-0.144E-01
1079	PHE	451	O	20100	GLY	192	C	1408	0.529E+00	-0.751E+00	-0.391E-01
1080	PHE	451	O	20100	LEU	224	C	1670	0.515E+00	-0.788E+00	-0.455E-01
1081	PHE	451	O	20100	GLY	225	N	1676	0.443E+00	0.743E+00	-0.129E+00
1082	PHE	451	O	20100	GLY	225	C	1678	0.368E+00	-0.187E+01	-0.212E+00
1083	PHE	451	O	20100	GLY	225	O	1679	0.334E+00	0.212E+01	-0.340E+00
1084	PHE	451	O	20100	ILE	226	C	1682	0.641E+00	-0.485E+00	-0.128E-01
1085	GLU	452	N	20108	GLY	192	C	1408	0.661E+00	-0.388E+00	-0.125E-01
1086	GLU	452	N	20108	LEU	224	C	1670	0.719E+00	-0.324E+00	-0.758E-02
1087	GLU	452	N	20108	GLY	225	C	1678	0.492E+00	-0.743E+00	-0.675E-01
1088	GLU	452	N	20108	GLY	225	O	1679	0.421E+00	0.957E+00	-0.165E+00
1089	GLU	452	N	20108	ILE	226	C	1682	0.678E+00	-0.361E+00	-0.108E-01
1090	GLU	452	CA	20109	GLY	225	C	1678	0.420E+00	0.832E-01	-0.127E+00
1091	GLU	452	CA	20109	GLY	225	O	1679	0.331E+00	-0.138E+00	-0.163E+00
1092	GLU	452	C	20110	GLN	112	OE1	771	0.710E+00	-0.405E+00	-0.700E-02
1093	GLU	452	C	20110	GLN	112	NE2	772	0.828E+00	-0.431E+00	-0.328E-02
1094	GLU	452	C	20110	LEU	224	O	1671	0.783E+00	-0.300E+00	-0.389E-02
1095	GLU	452	C	20110	GLY	225	O	1679	0.421E+00	-0.107E+01	-0.132E+00
1096	GLU	452	C	20110	ILE	226	N	1680	0.619E+00	-0.383E+00	-0.184E-01
1097	GLU	452	C	20110	ILE	226	O	1683	0.699E+00	-0.412E+00	-0.770E-02
1098	GLU	452	C	20110	ASN	227	N	1688	0.663E+00	-0.318E+00	-0.123E-01
1099	GLU	452	C	20110	ASN	227	ND2	1695	0.852E+00	-0.363E+00	-0.276E-02
1100	GLU	452	O	20111	GLN	112	CD	770	0.889E+00	-0.366E+00	-0.183E-02
1101	GLU	452	O	20111	GLY	192	C	1408	0.918E+00	-0.302E+00	-0.151E-02
1102	GLU	452	O	20111	GLY	225	C	1678	0.644E+00	-0.573E+00	-0.124E-01
1103	GLU	452	O	20111	ILE	226	C	1682	0.766E+00	-0.402E+00	-0.444E-02
1104	GLU	452	O	20111	ASN	227	CG	1693	0.897E+00	-0.315E+00	-0.172E-02
1105	GLU	452	CB	20112	GLY	225	C	1678	0.424E+00	0.190E+00	-0.123E+00
1106	GLU	452	CB	20112	GLY	225	O	1679	0.359E+00	-0.258E+00	-0.246E+00
1107	GLU	452	CG	20113	GLY	192	CA	1407	0.427E+00	0.188E-01	-0.134E+00
1108	GLU	452	CG	20113	GLY	225	CA	1677	0.399E+00	0.224E-01	-0.167E+00
1109	GLU	452	CG	20113	GLY	225	C	1678	0.356E+00	-0.137E+00	-0.115E+00
1110	GLU	452	CG	20113	GLY	225	O	1679	0.336E+00	0.140E+00	-0.201E+00
1111	GLU	452	CG	20113	ILE	226	N	1680	0.408E+00	0.741E-01	-0.184E+00
1112	GLU	452	CG	20113	ILE	226	CA	1681	0.440E+00	0.138E-01	-0.119E+00
1113	GLU	452	CG	20113	ILE	226	C	1682	0.412E+00	-0.910E-01	-0.136E+00
1114	GLU	452	CG	20113	ILE	226	O	1683	0.396E+00	0.110E+00	-0.193E+00
1115	GLU	452	CD	20114	SER	105	N	712	0.101E+01	-0.312E+00	-0.983E-03
1116	GLU	452	CD	20114	SER	105	OG	717	0.968E+00	-0.395E+00	-0.121E-02
1117	GLU	452	CD	20114	SER	111	OG	763	0.100E+01	-0.375E+00	-0.980E-03
1118	GLU	452	CD	20114	GLN	112	O	767	0.936E+00	-0.367E+00	-0.134E-02
1119	GLU	452	CD	20114	GLN	112	OE1	771	0.905E+00	-0.431E+00	-0.164E-02

GLU	452	CD	20114	THR	160	OG1	1142	0.105E+01	-0.331E+00	-0.755E-03
GLU	452	CD	20114	LYS	188	O	1375	0.995E+00	-0.334E+00	-0.930E-03
GLU	452	CD	20114	ASN	189	O	1384	0.870E+00	-0.383E+00	-0.208E-02
GLU	452	CD	20114	ASN	189	OD1	1387	0.700E+00	-0.569E+00	-0.763E-02
GLU	452	CD	20114	ASN	189	ND2	1388	0.736E+00	-0.766E+00	-0.660E-02
GLU	452	CD	20114	LYS	190	N	1389	0.772E+00	-0.391E+00	-0.497E-02
GLU	452	CD	20114	LYS	190	O	1392	0.803E+00	-0.472E+00	-0.336E-02
GLU	452	CD	20114	ASP	191	N	1398	0.741E+00	-0.540E+00	-0.635E-02
GLU	452	CD	20114	ASP	191	O	1401	0.680E+00	-0.571E+00	-0.900E-02
GLU	452	CD	20114	ASP	191	OD1	1404	0.961E+00	-0.455E+00	-0.114E-02
GLU	452	CD	20114	GLY	192	N	1406	0.515E+00	-0.777E+00	-0.527E-01
GLU	452	CD	20114	GLY	192	CA	1407	0.447E+00	-0.378E+00	-0.985E-01
GLU	452	CD	20114	GLY	192	O	1409	0.548E+00	-0.918E+00	-0.321E-01
GLU	452	CD	20114	PHE	193	N	1410	0.650E+00	-0.464E+00	-0.139E-01
GLU	452	CD	20114	PHE	193	O	1413	0.703E+00	-0.542E+00	-0.741E-02
GLU	452	CD	20114	LEU	222	O	1656	0.923E+00	-0.371E+00	-0.145E-02
GLU	452	CD	20114	LEU	224	O	1671	0.765E+00	-0.509E+00	-0.447E-02
GLU	452	CD	20114	GLY	225	N	1676	0.592E+00	-0.569E+00	-0.239E-01
GLU	452	CD	20114	GLY	225	O	1679	0.443E+00	-0.153E+01	-0.103E+00
GLU	452	CD	20114	ILE	226	N	1680	0.451E+00	-0.129E+01	-0.106E+00
GLU	452	CD	20114	ILE	226	C	1682	0.379E+00	0.256E+01	-0.139E+00
GLU	452	CD	20114	ILE	226	O	1683	0.340E+00	-0.373E+01	-0.193E+00
GLU	452	CD	20114	ASN	227	N	1688	0.415E+00	-0.152E+01	-0.153E+00
GLU	452	CD	20114	ASN	227	CA	1689	0.426E+00	0.149E+00	-0.121E+00
GLU	452	CD	20114	ASN	227	O	1691	0.663E+00	-0.629E+00	-0.105E-01
GLU	452	CD	20114	ASN	227	OD1	1694	0.522E+00	-0.106E+01	-0.422E-01
GLU	452	CD	20114	ASN	227	ND2	1695	0.610E+00	-0.111E+01	-0.200E-01
GLU	452	CD	20114	ILE	228	N	1696	0.573E+00	-0.736E+00	-0.288E-01
GLU	452	CD	20114	ILE	228	O	1699	0.781E+00	-0.545E+00	-0.396E-02
GLU	452	OE1	20115	GLY	104	C	710	0.100E+01	-0.368E+00	-0.901E-03
GLU	452	OE1	20115	ASN	189	C	1383	0.859E+00	-0.496E+00	-0.225E-02
GLU	452	OE1	20115	ASN	189	CG	1386	0.739E+00	-0.612E+00	-0.551E-02
GLU	452	OE1	20115	LYS	190	C	1391	0.855E+00	-0.588E+00	-0.231E-02
GLU	452	OE1	20115	ASP	191	C	1400	0.718E+00	-0.490E+00	-0.654E-02
GLU	452	OE1	20115	ASP	191	CG	1403	0.104E+01	-0.444E+00	-0.699E-03
GLU	452	OE1	20115	GLY	192	C	1408	0.659E+00	-0.760E+00	-0.108E-01
GLU	452	OE1	20115	PHE	193	C	1412	0.866E+00	-0.434E+00	-0.213E-02
GLU	452	OE1	20115	LEU	224	C	1670	0.833E+00	-0.486E+00	-0.270E-02
GLU	452	OE1	20115	GLY	225	C	1678	0.537E+00	-0.118E+01	-0.361E-01
GLU	452	OE1	20115	ILE	226	C	1682	0.426E+00	-0.203E+01	-0.126E+00
GLU	452	OE1	20115	ILE	226	O	1683	0.405E+00	0.251E+01	-0.175E+00
GLU	452	OE1	20115	ASN	227	N	1688	0.423E+00	0.156E+01	-0.162E+00
GLU	452	OE1	20115	ASN	227	CA	1689	0.407E+00	-0.180E+00	-0.173E+00
GLU	452	OE1	20115	ASN	227	C	1690	0.547E+00	-0.120E+01	-0.324E-01
GLU	452	OE1	20115	ASN	227	CB	1692	0.418E+00	0.351E+00	-0.154E+00
GLU	452	OE1	20115	ASN	227	CG	1693	0.446E+00	-0.185E+01	-0.999E-01
GLU	452	OE1	20115	ILE	228	C	1698	0.776E+00	-0.546E+00	-0.412E-02
GLU	452	OE2	20116	GLY	104	C	710	0.967E+00	-0.387E+00	-0.110E-02
GLU	452	OE2	20116	LYS	188	C	1374	0.883E+00	-0.557E+00	-0.190E-02
GLU	452	OE2	20116	ASN	189	C	1383	0.690E+00	-0.737E+00	-0.827E-02
GLU	452	OE2	20116	ASN	189	CG	1386	0.579E+00	-0.100E+01	-0.232E-01
GLU	452	OE2	20116	LYS	190	C	1391	0.694E+00	-0.857E+00	-0.803E-02
GLU	452	OE2	20116	ASP	191	C	1400	0.586E+00	-0.742E+00	-0.218E-01
GLU	452	OE2	20116	ASP	191	CG	1403	0.934E+00	-0.524E+00	-0.136E-02
GLU	452	OE2	20116	GLY	192	N	1406	0.458E+00	0.111E+01	-0.107E+00
GLU	452	OE2	20116	GLY	192	CA	1407	0.391E+00	0.573E+00	-0.202E+00
GLU	452	OE2	20116	GLY	192	C	1408	0.469E+00	-0.163E+01	-0.767E-01
GLU	452	OE2	20116	PHE	193	C	1412	0.649E+00	-0.739E+00	-0.119E-01
GLU	452	OE2	20116	LEU	194	C	1423	0.944E+00	-0.396E+00	-0.127E-02
GLU	452	OE2	20116	LEU	222	C	1655	0.958E+00	-0.387E+00	-0.117E-02
GLU	452	OE2	20116	LEU	224	C	1670	0.690E+00	-0.684E+00	-0.827E-02
GLU	452	OE2	20116	GLY	225	C	1678	0.453E+00	-0.177E+01	-0.918E-01
GLU	452	OE2	20116	ILE	226	N	1680	0.441E+00	0.147E+01	-0.131E+00
GLU	452	OE2	20116	ILE	226	C	1682	0.369E+00	-0.296E+01	-0.211E+00
GLU	452	OE2	20116	ASN	227	N	1688	0.425E+00	0.154E+01	-0.158E+00
GLU	452	OE2	20116	ASN	227	CA	1689	0.424E+00	-0.162E+00	-0.145E+00

1186	GLU	452	OE2	20116	ASN	227	C	1690	0.530E+00	-0.129E+01	-0.389E-01
1187	GLU	452	OE2	20116	ASN	227	CG	1693	0.542E+00	-0.116E+01	-0.342E-01
1188	GLU	452	OE2	20116	ILE	228	C	1698	0.721E+00	-0.625E+00	-0.639E-02
1189	ARG	453	N	20117	GLY	225	C	1678	0.520E+00	-0.464E+00	-0.498E-01
1190	ARG	453	N	20117	GLY	225	O	1679	0.402E+00	0.768E+00	-0.205E+00
1191	ARG	453	N	20117	ILE	226	C	1682	0.614E+00	-0.315E+00	-0.193E-01
1192	ARG	453	C	20119	SER	105	OG	717	0.879E+00	-0.439E+00	-0.216E-02
1193	ARG	453	C	20119	LYS	110	O	752	0.981E+00	-0.325E+00	-0.101E-02
1194	ARG	453	C	20119	SER	111	N	758	0.977E+00	-0.314E+00	-0.122E-02
1195	ARG	453	C	20119	SER	111	O	761	0.990E+00	-0.331E+00	-0.957E-03
1196	ARG	453	C	20119	SER	111	OG	763	0.633E+00	-0.805E+00	-0.152E-01
1197	ARG	453	C	20119	GLN	112	N	764	0.777E+00	-0.328E+00	-0.479E-02
1198	ARG	453	C	20119	GLN	112	O	767	0.826E+00	-0.430E+00	-0.283E-02
1199	ARG	453	C	20119	GLN	112	OE1	771	0.640E+00	-0.772E+00	-0.129E-01
1200	ARG	453	C	20119	GLN	112	NE2	772	0.712E+00	-0.880E+00	-0.809E-02
1201	ARG	453	C	20119	ASN	158	ND2	1130	0.919E+00	-0.500E+00	-0.176E-02
1202	ARG	453	C	20119	THR	160	OG1	1142	0.987E+00	-0.344E+00	-0.108E-02
1203	ARG	453	C	20119	LEU	224	O	1671	0.937E+00	-0.345E+00	-0.133E-02
1204	ARG	453	C	20119	GLY	225	O	1679	0.566E+00	-0.813E+00	-0.265E-01
1205	ARG	453	C	20119	ILE	226	N	1680	0.721E+00	-0.438E+00	-0.745E-02
1206	ARG	453	C	20119	ILE	226	O	1683	0.783E+00	-0.518E+00	-0.389E-02
1207	ARG	453	C	20119	ASN	227	N	1688	0.643E+00	-0.523E+00	-0.147E-01
1208	ARG	453	C	20119	ASN	227	O	1691	0.861E+00	-0.371E+00	-0.220E-02
1209	ARG	453	C	20119	ASN	227	OD1	1694	0.875E+00	-0.364E+00	-0.201E-02
1210	ARG	453	C	20119	ASN	227	ND2	1695	0.771E+00	-0.671E+00	-0.500E-02
1211	ARG	453	O	20120	LYS	110	C	751	0.978E+00	-0.333E+00	-0.103E-02
1212	ARG	453	O	20120	SER	111	C	760	0.794E+00	-0.312E+00	-0.360E-02
1213	ARG	453	O	20120	GLN	112	CD	770	0.646E+00	-0.639E+00	-0.123E-01
1214	ARG	453	O	20120	GLY	225	C	1678	0.609E+00	-0.628E+00	-0.173E-01
1215	ARG	453	O	20120	ILE	226	C	1682	0.580E+00	-0.682E+00	-0.230E-01
1216	ARG	453	O	20120	ASN	227	C	1690	0.731E+00	-0.463E+00	-0.587E-02
1217	ARG	453	O	20120	ASN	227	CG	1693	0.679E+00	-0.505E+00	-0.912E-02
1218	ARG	453	CD	20123	GLN	112	NE2	772	0.468E+00	-0.380E+00	-0.995E-01
1219	ARG	453	NE	20124	SER	111	OG	763	0.785E+00	-0.339E+00	-0.518E-02
1220	ARG	453	NE	20124	GLN	112	CD	770	0.414E+00	0.145E+01	-0.155E+00
1221	ARG	453	NE	20124	GLN	112	OE1	771	0.422E+00	-0.129E+01	-0.164E+00
1222	ARG	453	NE	20124	GLN	112	NE2	772	0.340E+00	-0.324E+01	-0.196E+00
1223	ARG	453	NE	20124	ASN	129	ND2	898	0.902E+00	-0.328E+00	-0.241E-02
1224	ARG	453	NE	20124	ASN	158	O	1126	0.701E+00	-0.343E+00	-0.920E-02
1225	ARG	453	NE	20124	ASN	158	OD1	1129	0.548E+00	-0.577E+00	-0.393E-01
1226	ARG	453	NE	20124	ASN	158	ND2	1130	0.501E+00	-0.105E+01	-0.761E-01
1227	ARG	453	NE	20124	THR	160	OG1	1142	0.695E+00	-0.401E+00	-0.107E-01
1228	ARG	453	NE	20124	GLY	225	O	1679	0.672E+00	-0.362E+00	-0.119E-01
1229	ARG	453	CZ	20125	SER	111	OG	763	0.879E+00	-0.341E+00	-0.216E-02
1230	ARG	453	CZ	20125	GLN	112	OE1	771	0.497E+00	-0.105E+01	-0.557E-01
1231	ARG	453	CZ	20125	GLN	112	NE2	772	0.365E+00	-0.325E+01	-0.192E+00
1232	ARG	453	CZ	20125	ASN	129	OD1	897	0.814E+00	-0.319E+00	-0.309E-02
1233	ARG	453	CZ	20125	ASN	129	ND2	898	0.922E+00	-0.385E+00	-0.172E-02
1234	ARG	453	CZ	20125	ASN	158	O	1126	0.675E+00	-0.448E+00	-0.939E-02
1235	ARG	453	CZ	20125	ASN	158	OD1	1129	0.474E+00	-0.988E+00	-0.724E-01
1236	ARG	453	CZ	20125	ASN	158	ND2	1130	0.453E+00	-0.163E+01	-0.103E+00
1237	ARG	453	CZ	20125	CYS	159	O	1134	0.852E+00	-0.328E+00	-0.236E-02
1238	ARG	453	CZ	20125	THR	160	OG1	1142	0.723E+00	-0.452E+00	-0.694E-02
1239	ARG	453	CZ	20125	GLY	225	O	1679	0.786E+00	-0.327E+00	-0.381E-02
1240	ARG	453	NH1	20126	GLN	112	CD	770	0.601E+00	-0.881E+00	-0.218E-01
1241	ARG	453	NH1	20126	ASN	129	CG	896	0.101E+01	-0.302E+00	-0.975E-03
1242	ARG	453	NH1	20126	ASN	158	C	1125	0.840E+00	-0.428E+00	-0.300E-02
1243	ARG	453	NH1	20126	ASN	158	CG	1128	0.600E+00	-0.772E+00	-0.220E-01
1244	ARG	453	NH1	20126	CYS	159	C	1133	0.921E+00	-0.385E+00	-0.174E-02
1245	ARG	453	NH1	20126	THR	160	C	1139	0.962E+00	-0.313E+00	-0.134E-02
1246	ARG	453	NH1	20126	GLY	225	C	1678	0.979E+00	-0.316E+00	-0.120E-02
1247	ARG	453	NH2	20127	SER	111	C	760	0.889E+00	-0.305E+00	-0.214E-02
1248	ARG	453	NH2	20127	GLN	112	CD	770	0.425E+00	-0.199E+01	-0.139E+00
1249	ARG	453	NH2	20127	ALA	127	C	882	0.893E+00	-0.358E+00	-0.209E-02
1250	ARG	453	NH2	20127	CYS	128	C	887	0.901E+00	-0.398E+00	-0.197E-02
1251	ARG	453	NH2	20127	ASN	129	CG	896	0.821E+00	-0.422E+00	-0.345E-02

1252	ARG	453	NH2	20127	PHE	157	C	1114	0.842E+00	-0.379E+00	-0.297E-02
1253	ARG	453	NH2	20127	ASN	158	C	1125	0.615E+00	-0.776E+00	-0.192E-01
1254	ARG	453	NH2	20127	ASN	158	CG	1128	0.388E+00	-0.221E+01	-0.188E+00
1255	ARG	453	NH2	20127	ASN	158	OD1	1129	0.342E+00	0.279E+01	-0.303E+00
1256	ARG	453	NH2	20127	ASN	158	ND2	1130	0.350E+00	0.390E+01	-0.253E+00
1257	ARG	453	NH2	20127	CYS	159	C	1133	0.715E+00	-0.597E+00	-0.784E-02
1258	ARG	453	NH2	20127	THR	160	C	1139	0.797E+00	-0.426E+00	-0.411E-02
1259	ARG	453	NH2	20127	THR	160	CB	1141	0.583E+00	-0.335E+00	-0.293E-01
1260	ARG	453	NH2	20127	THR	160	CG2	1143	0.436E+00	0.491E+00	-0.140E+00
1261	ARG	453	NH2	20127	LEU	224	C	1670	0.100E+01	-0.301E+00	-0.105E-02
1262	ARG	453	NH2	20127	GLY	225	C	1678	0.917E+00	-0.350E+00	-0.178E-02
1263	ASP	454	N	20128	LYS	110	C	751	0.101E+01	-0.308E+00	-0.102E-02
1264	ASP	454	N	20128	GLN	112	CD	770	0.740E+00	-0.473E+00	-0.643E-02
1265	ASP	454	N	20128	GLY	225	C	1678	0.811E+00	-0.349E+00	-0.372E-02
1266	ASP	454	N	20128	ILE	226	C	1682	0.805E+00	-0.346E+00	-0.387E-02
1267	ASP	454	N	20128	ASN	227	CG	1693	0.842E+00	-0.328E+00	-0.296E-02
1268	ASP	454	C	20130	SER	111	OG	763	0.599E+00	-0.550E+00	-0.211E-01
1269	ASP	454	C	20130	GLN	112	OE1	771	0.789E+00	-0.314E+00	-0.372E-02
1270	ASP	454	C	20130	GLN	112	NE2	772	0.773E+00	-0.458E+00	-0.493E-02
1271	ASP	454	C	20130	ASN	158	ND2	1130	0.866E+00	-0.333E+00	-0.250E-02
1272	ASP	454	O	20131	LYS	110	C	751	0.947E+00	-0.303E+00	-0.125E-02
1273	ASP	454	O	20131	GLN	112	CD	770	0.884E+00	-0.311E+00	-0.188E-02
1274	ASP	454	CG	20133	SER	105	N	712	0.973E+00	-0.323E+00	-0.125E-02
1275	ASP	454	CG	20133	SER	105	O	715	0.994E+00	-0.336E+00	-0.937E-03
1276	ASP	454	CG	20133	SER	105	OG	717	0.701E+00	-0.671E+00	-0.834E-02
1277	ASP	454	CG	20133	THR	106	OG1	723	0.954E+00	-0.370E+00	-0.132E-02
1278	ASP	454	CG	20133	LYS	110	O	752	0.828E+00	-0.435E+00	-0.279E-02
1279	ASP	454	CG	20133	SER	111	N	758	0.852E+00	-0.399E+00	-0.276E-02
1280	ASP	454	CG	20133	SER	111	O	761	0.977E+00	-0.345E+00	-0.104E-02
1281	ASP	454	CG	20133	SER	111	OG	763	0.580E+00	-0.992E+00	-0.255E-01
1282	ASP	454	CG	20133	GLN	112	N	764	0.826E+00	-0.301E+00	-0.333E-02
1283	ASP	454	CG	20133	GLN	112	O	767	0.910E+00	-0.374E+00	-0.159E-02
1284	ASP	454	CG	20133	GLN	112	OE1	771	0.941E+00	-0.395E+00	-0.130E-02
1285	ASP	454	CG	20133	GLN	112	NE2	772	0.982E+00	-0.519E+00	-0.118E-02
1286	ASP	454	CG	20133	GLY	225	O	1679	0.937E+00	-0.322E+00	-0.133E-02
1287	ASP	454	CG	20133	ILE	226	O	1683	0.997E+00	-0.357E+00	-0.919E-03
1288	ASP	454	CG	20133	ASN	227	N	1688	0.790E+00	-0.361E+00	-0.435E-02
1289	ASP	454	CG	20133	ASN	227	O	1691	0.857E+00	-0.383E+00	-0.228E-02
1290	ASP	454	CG	20133	ASN	227	OD1	1694	0.884E+00	-0.366E+00	-0.189E-02
1291	ASP	454	CG	20133	ASN	227	ND2	1695	0.699E+00	-0.824E+00	-0.901E-02
1292	ASP	454	OD1	20134	GLY	104	C	710	0.103E+01	-0.313E+00	-0.762E-03
1293	ASP	454	OD1	20134	SER	105	C	714	0.903E+00	-0.317E+00	-0.167E-02
1294	ASP	454	OD1	20134	LYS	110	C	751	0.759E+00	-0.641E+00	-0.470E-02
1295	ASP	454	OD1	20134	SER	111	C	760	0.747E+00	-0.440E+00	-0.517E-02
1296	ASP	454	OD1	20134	GLN	112	CD	770	0.876E+00	-0.460E+00	-0.199E-02
1297	ASP	454	OD1	20134	GLY	225	C	1678	0.102E+01	-0.316E+00	-0.788E-03
1298	ASP	454	OD1	20134	ILE	226	C	1682	0.891E+00	-0.382E+00	-0.180E-02
1299	ASP	454	OD1	20134	ASN	227	C	1690	0.893E+00	-0.412E+00	-0.178E-02
1300	ASP	454	OD1	20134	ASN	227	CG	1693	0.800E+00	-0.470E+00	-0.344E-02
1301	ASP	454	OD2	20135	LYS	110	C	751	0.956E+00	-0.436E+00	-0.118E-02
1302	ASP	454	OD2	20135	ILE	226	C	1682	0.941E+00	-0.350E+00	-0.130E-02
1303	ASP	454	OD2	20135	ASN	227	C	1690	0.906E+00	-0.403E+00	-0.163E-02
1304	ASP	454	OD2	20135	ASN	227	CG	1693	0.739E+00	-0.542E+00	-0.549E-02
1305	ILE	455	N	20136	LYS	110	C	751	0.765E+00	-0.390E+00	-0.526E-02
1306	ILE	455	N	20136	GLN	112	CD	770	0.680E+00	-0.449E+00	-0.105E-01
1307	ILE	455	C	20138	LYS	110	O	752	0.550E+00	-0.749E+00	-0.314E-01
1308	ILE	455	C	20138	SER	111	N	758	0.693E+00	-0.443E+00	-0.948E-02
1309	ILE	455	C	20138	SER	111	O	761	0.841E+00	-0.334E+00	-0.255E-02
1310	ILE	455	C	20138	SER	111	OG	763	0.591E+00	-0.725E+00	-0.227E-01
1311	ILE	455	C	20138	GLN	112	OE1	771	0.865E+00	-0.345E+00	-0.215E-02
1312	ILE	455	C	20138	GLN	112	NE2	772	0.784E+00	-0.573E+00	-0.453E-02
1313	ILE	455	C	20138	ASN	129	OD1	897	0.840E+00	-0.304E+00	-0.256E-02
1314	ILE	455	C	20138	ASN	129	ND2	898	0.790E+00	-0.502E+00	-0.435E-02
1315	ILE	455	C	20138	ASN	158	ND2	1130	0.764E+00	-0.532E+00	-0.530E-02
1316	ILE	455	O	20139	ASN	109	C	743	0.925E+00	-0.331E+00	-0.144E-02
1317	ILE	455	O	20139	LYS	110	C	751	0.660E+00	-0.713E+00	-0.108E-01

1318	ILE	455	O	20139	SER	111	C	760	0.779E+00	-0.346E+00	-0.403E-02
1319	ILE	455	O	20139	GLN	112	CD	770	0.863E+00	-0.401E+00	-0.218E-02
1320	ILE	455	O	20139	ASN	129	CG	896	0.860E+00	-0.353E+00	-0.223E-02
1321	ILE	455	O	20139	ASN	158	CG	1128	0.895E+00	-0.330E+00	-0.176E-02
1322	ILE	455	CD1	20143	GLN	112	CG	769	0.446E+00	0.779E-02	-0.113E+00
1323	ILE	455	CD1	20143	GLN	112	NE2	772	0.414E+00	0.415E+00	-0.174E+00
1324	SER	456	N	20144	LYS	110	C	751	0.618E+00	-0.712E+00	-0.187E-01
1325	SER	456	N	20144	SER	111	C	760	0.701E+00	-0.367E+00	-0.881E-02
1326	SER	456	N	20144	GLN	112	CD	770	0.836E+00	-0.369E+00	-0.309E-02
1327	SER	456	C	20146	LYS	110	O	752	0.592E+00	-0.541E+00	-0.205E-01
1328	SER	456	C	20146	SER	111	N	758	0.742E+00	-0.330E+00	-0.631E-02
1329	SER	456	C	20146	SER	111	OG	763	0.705E+00	-0.430E+00	-0.807E-02
1330	SER	456	C	20146	ASN	129	ND2	898	0.941E+00	-0.318E+00	-0.152E-02
1331	SER	456	O	20147	LYS	110	C	751	0.787E+00	-0.478E+00	-0.379E-02
1332	SER	456	CB	20148	SER	111	CB	762	0.451E+00	0.806E-01	-0.106E+00
1333	SER	456	OG	20149	THR	106	C	720	0.915E+00	-0.315E+00	-0.169E-02
1334	SER	456	OG	20149	ASN	108	C	735	0.979E+00	-0.313E+00	-0.113E-02
1335	SER	456	OG	20149	ASN	108	CG	738	0.910E+00	-0.332E+00	-0.175E-02
1336	SER	456	OG	20149	ASN	109	C	743	0.819E+00	-0.417E+00	-0.329E-02
1337	SER	456	OG	20149	LYS	110	C	751	0.518E+00	-0.124E+01	-0.484E-01
1338	SER	456	OG	20149	SER	111	C	760	0.714E+00	-0.419E+00	-0.745E-02
1339	SER	456	OG	20149	ASN	129	CG	896	0.962E+00	-0.304E+00	-0.126E-02
1340	ASN	457	N	20150	LYS	110	C	751	0.709E+00	-0.429E+00	-0.825E-02
1341	ASN	457	C	20152	LYS	110	O	752	0.773E+00	-0.407E+00	-0.420E-02
1342	ASN	457	C	20152	SER	111	OG	763	0.991E+00	-0.308E+00	-0.105E-02
1343	ASN	457	O	20153	LYS	110	C	751	0.903E+00	-0.342E+00	-0.166E-02
1344	VAL	458	N	20158	LYS	110	C	751	0.860E+00	-0.318E+00	-0.262E-02
1345	GLY	464	C	20206	SER	105	O	715	0.852E+00	-0.334E+00	-0.235E-02
1346	GLY	464	C	20206	SER	105	OG	717	0.886E+00	-0.345E+00	-0.206E-02
1347	GLY	464	C	20206	ASN	227	ND2	1695	0.869E+00	-0.435E+00	-0.246E-02
1348	GLY	464	C	20206	THR	229	OG1	1709	0.808E+00	-0.378E+00	-0.356E-02
1349	GLY	464	C	20206	ASN	230	ND2	1718	0.102E+01	-0.338E+00	-0.928E-03
1350	LYS	465	C	20210	SER	105	O	715	0.860E+00	-0.410E+00	-0.223E-02
1351	LYS	465	C	20210	SER	105	OG	717	0.952E+00	-0.384E+00	-0.134E-02
1352	LYS	465	C	20210	THR	229	OG1	1709	0.980E+00	-0.346E+00	-0.112E-02
1353	LYS	465	C	20210	ASN	230	OD1	1717	0.982E+00	-0.302E+00	-0.100E-02
1354	SER	500	OG	20496	ASP	191	CG	1403	0.950E+00	-0.396E+00	-0.136E-02
1355	PHE	501	C	20499	ASP	191	O	1401	0.724E+00	-0.362E+00	-0.619E-02
1356	PHE	501	C	20499	ASP	191	OD1	1404	0.781E+00	-0.460E+00	-0.395E-02
1357	PHE	501	C	20499	ASP	191	OD2	1405	0.752E+00	-0.492E+00	-0.495E-02
1358	PHE	501	O	20500	ASP	191	CG	1403	0.685E+00	-0.556E+00	-0.868E-02
1359	GLU	502	N	20508	ASP	191	CG	1403	0.714E+00	-0.427E+00	-0.790E-02
1360	GLU	502	C	20510	LYS	190	O	1392	0.724E+00	-0.350E+00	-0.623E-02
1361	GLU	502	C	20510	ASP	191	N	1398	0.734E+00	-0.338E+00	-0.674E-02
1362	GLU	502	C	20510	ASP	191	O	1401	0.469E+00	-0.802E+00	-0.770E-01
1363	GLU	502	C	20510	ASP	191	OD1	1404	0.641E+00	-0.576E+00	-0.128E-01
1364	GLU	502	C	20510	ASP	191	OD2	1405	0.583E+00	-0.706E+00	-0.224E-01
1365	GLU	502	C	20510	PHE	193	CE1	1418	0.436E+00	-0.301E+00	-0.976E-01
1366	GLU	502	C	20510	PHE	193	CZ	1420	0.412E+00	-0.222E+00	-0.121E+00
1367	GLU	502	O	20511	LYS	190	C	1391	0.785E+00	-0.490E+00	-0.384E-02
1368	GLU	502	O	20511	ASP	191	C	1400	0.585E+00	-0.536E+00	-0.220E-01
1369	GLU	502	O	20511	ASP	191	CG	1403	0.630E+00	-0.769E+00	-0.142E-01
1370	GLU	502	O	20511	GLY	192	C	1408	0.694E+00	-0.495E+00	-0.803E-02
1371	GLU	502	O	20511	PHE	193	C	1412	0.883E+00	-0.303E+00	-0.190E-02
1372	GLU	502	O	20511	PHE	193	CE1	1418	0.342E+00	0.720E+00	-0.200E+00
1373	GLU	502	O	20511	PHE	193	CE2	1419	0.439E+00	0.373E+00	-0.108E+00
1374	GLU	502	O	20511	LYS	221	C	1646	0.903E+00	-0.386E+00	-0.166E-02
1375	GLU	502	O	20511	LEU	222	C	1655	0.821E+00	-0.358E+00	-0.293E-02
1376	GLU	502	CG	20513	ASP	191	CB	1402	0.371E+00	0.101E-01	-0.172E+00
1377	GLU	502	CG	20513	ASP	191	CG	1403	0.366E+00	-0.163E+00	-0.145E+00
1378	GLU	502	CG	20513	ASP	191	OD1	1404	0.452E+00	0.924E-01	-0.105E+00
1379	GLU	502	CG	20513	ASP	191	OD2	1405	0.331E+00	0.210E+00	-0.164E+00
1380	GLU	502	CD	20514	GLU	45	OE2	229	0.102E+01	-0.474E+00	-0.820E-03
1381	GLU	502	CD	20514	LYS	190	O	1392	0.642E+00	-0.721E+00	-0.127E-01
1382	GLU	502	CD	20514	LYS	190	NZ	1397	0.614E+00	-0.352E+00	-0.194E-01
1383	GLU	502	CD	20514	ASP	191	N	1398	0.676E+00	-0.644E+00	-0.109E-01

GLU	502	CD	20514	ASP	191	O	1401	0.587E+00	-0.775E+00	-0.215E-01
GLU	502	CD	20514	ASP	191	CB	1402	0.444E+00	-0.142E+00	-0.101E+00
GLU	502	CD	20514	ASP	191	OD1	1404	0.545E+00	-0.133E+01	-0.331E-01
GLU	502	CD	20514	ASP	191	OD2	1405	0.372E+00	-0.344E+01	-0.208E+00
GLU	502	CD	20514	GLY	192	N	1406	0.769E+00	-0.338E+00	-0.509E-02
GLU	502	CD	20514	GLY	192	O	1409	0.913E+00	-0.344E+00	-0.155E-02
GLU	502	CD	20514	PHE	193	N	1410	0.769E+00	-0.336E+00	-0.511E-02
GLU	502	CD	20514	PHE	193	O	1413	0.962E+00	-0.316E+00	-0.114E-02
GLU	502	CD	20514	PHE	193	CE1	1418	0.527E+00	-0.310E+00	-0.375E-01
GLU	502	CD	20514	PHE	193	CE2	1419	0.412E+00	-0.566E+00	-0.121E+00
GLU	502	CD	20514	PHE	193	CZ	1420	0.426E+00	-0.330E+00	-0.107E+00
GLU	502	CD	20514	TYR	195	OH	1440	0.547E+00	-0.762E+00	-0.356E-01
GLU	502	CD	20514	LYS	221	O	1647	0.909E+00	-0.383E+00	-0.160E-02
GLU	502	CD	20514	LYS	221	NZ	1652	0.513E+00	-0.525E+00	-0.541E-01
GLU	502	CD	20514	LEU	222	O	1656	0.986E+00	-0.335E+00	-0.981E-03
GLU	502	OE1	20515	GLU	45	CD	227	0.101E+01	-0.479E+00	-0.860E-03
GLU	502	OE1	20515	LYS	190	C	1391	0.812E+00	-0.641E+00	-0.313E-02
GLU	502	OE1	20515	ASP	191	C	1400	0.765E+00	-0.436E+00	-0.448E-02
GLU	502	OE1	20515	ASP	191	CG	1403	0.550E+00	-0.143E+01	-0.314E-01
GLU	502	OE1	20515	GLY	192	C	1408	0.953E+00	-0.396E+00	-0.120E-02
GLU	502	OE1	20515	PHE	193	C	1412	0.104E+01	-0.329E+00	-0.722E-03
GLU	502	OE1	20515	LYS	221	C	1646	0.972E+00	-0.479E+00	-0.107E-02
GLU	502	OE1	20515	LEU	222	C	1655	0.105E+01	-0.339E+00	-0.680E-03
GLU	502	OE2	20516	GLU	45	CD	227	0.104E+01	-0.459E+00	-0.718E-03
GLU	502	OE2	20516	ASN	189	C	1383	0.952E+00	-0.421E+00	-0.121E-02
GLU	502	OE2	20516	LYS	190	C	1391	0.614E+00	-0.110E+01	-0.165E-01
GLU	502	OE2	20516	ASP	191	C	1400	0.602E+00	-0.701E+00	-0.186E-01
GLU	502	OE2	20516	ASP	191	CB	1402	0.412E+00	0.186E+00	-0.164E+00
GLU	502	OE2	20516	ASP	191	CG	1403	0.427E+00	-0.264E+01	-0.124E+00
GLU	502	OE2	20516	ASP	191	OD2	1405	0.356E+00	0.417E+01	-0.302E+00
GLU	502	OE2	20516	GLY	192	C	1408	0.768E+00	-0.568E+00	-0.439E-02
GLU	502	OE2	20516	PHE	193	C	1412	0.825E+00	-0.471E+00	-0.285E-02
GLU	502	OE2	20516	PHE	193	CD2	1417	0.392E+00	0.365E+00	-0.177E+00
GLU	502	OE2	20516	PHE	193	CZ	1420	0.363E+00	0.541E+00	-0.216E+00
GLU	502	OE2	20516	LEU	194	C	1423	0.982E+00	-0.373E+00	-0.100E-02
GLU	502	OE2	20516	TYR	195	CZ	1439	0.553E+00	-0.392E+00	-0.304E-01
GLU	502	OE2	20516	TYR	195	OH	1440	0.435E+00	0.142E+01	-0.134E+00
GLU	502	OE2	20516	PHE	220	C	1635	0.103E+01	-0.334E+00	-0.769E-03
GLU	502	OE2	20516	LYS	221	C	1646	0.804E+00	-0.652E+00	-0.332E-02
GLU	502	OE2	20516	LYS	221	NZ	1652	0.419E+00	0.926E+00	-0.169E+00
GLU	502	OE2	20516	LEU	222	C	1655	0.876E+00	-0.446E+00	-0.200E-02

Table S3 6M17BchaintoEchain-finedata.txt

ILE	21	C	3	ASN	487	ND2	2345	0.865E+00	-0.429E+00	-0.252E-02
ILE	21	O	4	ALA	475	C	2195	0.866E+00	-0.340E+00	-0.213E-02
ILE	21	O	4	GLY	476	C	2205	0.921E+00	-0.314E+00	-0.148E-02
ILE	21	O	4	ASN	487	CG	2343	0.832E+00	-0.373E+00	-0.272E-02
ILE	21	O	4	ASN	4872HD2		2351	0.703E+00	-0.308E+00	-0.207E-03
ILE	21	HT2	10	ASN	487	ND2	2345	0.673E+00	-0.392E+00	-0.366E-03
GLU	23	C	39	ASN	487	ND2	2345	0.829E+00	-0.381E+00	-0.325E-02
GLU	23	O	40	ALA	475	C	2195	0.765E+00	-0.403E+00	-0.447E-02
GLU	23	O	40	ASN	487	C	2340	0.956E+00	-0.301E+00	-0.118E-02
GLU	23	O	40	ASN	487	CG	2343	0.887E+00	-0.321E+00	-0.185E-02
GLU	23	CD	43	GLN	474	O	2179	0.100E+01	-0.331E+00	-0.891E-03
GLU	23	CD	43	ALA	475	O	2196	0.862E+00	-0.412E+00	-0.220E-02
GLU	23	CD	43	GLY	476	N	2203	0.682E+00	-0.424E+00	-0.104E-01
GLU	23	CD	43	GLY	476	O	2206	0.931E+00	-0.334E+00	-0.138E-02
GLU	23	CD	43	SER	477	N	2210	0.852E+00	-0.409E+00	-0.276E-02
GLU	23	CD	43	ASN	487	ND2	2345	0.929E+00	-0.514E+00	-0.165E-02
GLU	23	OE1	44	ALA	475	C	2195	0.723E+00	-0.623E+00	-0.630E-02
GLU	23	OE1	44	GLY	476	C	2205	0.751E+00	-0.591E+00	-0.500E-02
GLU	23	OE1	44	GLY	476	HN	2207	0.551E+00	-0.487E+00	-0.886E-03
GLU	23	OE1	44	SER	477	C	2212	0.101E+01	-0.303E+00	-0.866E-03
GLU	23	OE1	44	SER	477	HN	2216	0.683E+00	-0.421E+00	-0.245E-03
GLU	23	OE1	44	ASN	487	CG	2343	0.101E+01	-0.365E+00	-0.857E-03

1450	GLU	23 OE1	44 ASN	4872HD2	2351	0.832E+00	-0.301E+00	-0.752E-04
1451	GLU	23 OE2	45 ALA	475 C	2195	0.886E+00	-0.435E+00	-0.186E-02
1452	GLU	23 OE2	45 GLY	476 C	2205	0.930E+00	-0.411E+00	-0.139E-02
1453	GLN	24 N	52 ALA	475 C	2195	0.695E+00	-0.315E+00	-0.928E-02
1454	GLN	24 C	54 ASN	487 OD1	2344	0.646E+00	-0.365E+00	-0.122E-01
1455	GLN	24 C	54 ASN	487 ND2	2345	0.690E+00	-0.476E+00	-0.974E-02
1456	GLN	24 C	54 TYR	489 OH	2373	0.618E+00	-0.320E+00	-0.175E-01
1457	GLN	24 O	55 ALA	475 C	2195	0.746E+00	-0.403E+00	-0.521E-02
1458	GLN	24 O	55 ASN	487 C	2340	0.738E+00	-0.445E+00	-0.557E-02
1459	GLN	24 O	55 ASN	487 CG	2343	0.659E+00	-0.524E+00	-0.108E-01
1460	GLN	24 O	55 ASN	4872HD2	2351	0.648E+00	-0.329E+00	-0.335E-03
1461	GLN	24 O	55 TYR	489 HH	2382	0.497E+00	-0.552E+00	0.000E+00
1462	GLN	24 CG	57 ALA	475 C	2195	0.434E+00	-0.469E-01	-0.112E+00
1463	GLN	24 CG	57 ALA	475 CB	2197	0.432E+00	0.191E-01	-0.128E+00
1464	GLN	24 CG	57 GLY	476 N	2203	0.410E+00	0.356E-01	-0.180E+00
1465	GLN	24 CG	57 GLY	476 CA	2204	0.418E+00	0.117E-01	-0.147E+00
1466	GLN	24 CG	57 ASN	487 ND2	2345	0.431E+00	0.657E-01	-0.148E+00
1467	GLN	24 CD	58 GLN	474 O	2179	0.806E+00	-0.411E+00	-0.329E-02
1468	GLN	24 CD	58 GLN	474 OE1	2183	0.818E+00	-0.445E+00	-0.300E-02
1469	GLN	24 CD	58 GLN	474 NE2	2184	0.104E+01	-0.431E+00	-0.862E-03
1470	GLN	24 CD	58 ALA	475 N	2193	0.637E+00	-0.461E+00	-0.156E-01
1471	GLN	24 CD	58 ALA	475 C	2195	0.433E+00	0.158E+01	-0.100E+00
1472	GLN	24 CD	58 ALA	475 O	2196	0.432E+00	-0.155E+01	-0.117E+00
1473	GLN	24 CD	58 ALA	475 CB	2197	0.508E+00	-0.430E+00	-0.513E-01
1474	GLN	24 CD	58 GLY	476 N	2203	0.397E+00	-0.130E+01	-0.178E+00
1475	GLN	24 CD	58 GLY	476 CA	2204	0.353E+00	-0.611E+00	-0.102E+00
1476	GLN	24 CD	58 GLY	476 O	2206	0.536E+00	-0.841E+00	-0.362E-01
1477	GLN	24 CD	58 SER	477 N	2210	0.564E+00	-0.797E+00	-0.315E-01
1478	GLN	24 CD	58 SER	477 O	2213	0.689E+00	-0.565E+00	-0.837E-02
1479	GLN	24 CD	58 SER	477 OG	2215	0.894E+00	-0.391E+00	-0.195E-02
1480	GLN	24 CD	58 THR	478 OG1	2226	0.929E+00	-0.346E+00	-0.155E-02
1481	GLN	24 CD	58 PHE	486 O	2321	0.843E+00	-0.341E+00	-0.250E-02
1482	GLN	24 CD	58 ASN	487 N	2338	0.766E+00	-0.342E+00	-0.522E-02
1483	GLN	24 CD	58 ASN	487 O	2341	0.675E+00	-0.530E+00	-0.946E-02
1484	GLN	24 CD	58 ASN	487 OD1	2344	0.453E+00	-0.130E+01	-0.919E-01
1485	GLN	24 CD	58 ASN	487 ND2	2345	0.365E+00	-0.337E+01	-0.193E+00
1486	GLN	24 OE1	59 GLN	474 CD	2182	0.104E+01	-0.303E+00	-0.696E-03
1487	GLN	24 OE1	59 ALA	475 C	2195	0.549E+00	-0.839E+00	-0.317E-01
1488	GLN	24 OE1	59 GLY	476 CA	2204	0.441E+00	0.320E+00	-0.119E+00
1489	GLN	24 OE1	59 GLY	476 C	2205	0.559E+00	-0.822E+00	-0.285E-01
1490	GLN	24 OE1	59 GLY	476 HN	2207	0.552E+00	-0.370E+00	-0.881E-03
1491	GLN	24 OE1	59 GLY	476 HA2	2209	0.361E+00	-0.374E+00	-0.556E-01
1492	GLN	24 OE1	59 SER	477 C	2212	0.781E+00	-0.349E+00	-0.396E-02
1493	GLN	24 OE1	59 SER	477 HN	2216	0.602E+00	-0.415E+00	-0.523E-03
1494	GLN	24 OE1	59 PHE	486 C	2320	0.897E+00	-0.313E+00	-0.173E-02
1495	GLN	24 OE1	59 ASN	487 C	2340	0.808E+00	-0.419E+00	-0.323E-02
1496	GLN	24 OE1	59 ASN	487 CG	2343	0.505E+00	-0.104E+01	-0.508E-01
1497	GLN	24 OE1	59 ASN	487 ND2	2345	0.434E+00	0.202E+01	-0.142E+00
1498	GLN	24 OE1	59 ASN	4871HD2	2350	0.497E+00	-0.659E+00	-0.165E-02
1499	GLN	24 OE1	59 ASN	4872HD2	2351	0.337E+00	-0.180E+01	-0.163E-01
1500	GLN	24 NE2	60 TYR	473 C	2157	0.948E+00	-0.458E+00	-0.146E-02
1501	GLN	24 NE2	60 GLN	474 C	2178	0.667E+00	-0.573E+00	-0.118E-01
1502	GLN	24 NE2	60 GLN	474 CD	2182	0.821E+00	-0.622E+00	-0.346E-02
1503	GLN	24 NE2	60 GLN	4742HE2	2192	0.903E+00	-0.324E+00	-0.629E-04
1504	GLN	24 NE2	60 ALA	475 CA	2194	0.461E+00	0.878E-01	-0.107E+00
1505	GLN	24 NE2	60 ALA	475 C	2195	0.335E+00	-0.413E+01	-0.283E-01
1506	GLN	24 NE2	60 ALA	475 O	2196	0.323E+00	0.446E+01	-0.199E+00
1507	GLN	24 NE2	60 ALA	475 HN	2198	0.544E+00	-0.621E+00	-0.130E-02
1508	GLN	24 NE2	60 GLY	476 C	2205	0.357E+00	-0.353E+01	-0.177E+00
1509	GLN	24 NE2	60 GLY	476 O	2206	0.404E+00	0.222E+01	-0.200E+00
1510	GLN	24 NE2	60 GLY	476 HN	2207	0.365E+00	-0.145E+01	-0.138E-01
1511	GLN	24 NE2	60 SER	477 N	2210	0.448E+00	0.182E+01	-0.137E+00
1512	GLN	24 NE2	60 SER	477 C	2212	0.604E+00	-0.813E+00	-0.213E-01
1513	GLN	24 NE2	60 SER	477 HN	2216	0.448E+00	-0.116E+01	-0.415E-02
1514	GLN	24 NE2	60 SER	477 HG	2220	0.768E+00	-0.467E+00	-0.166E-03
1515	GLN	24 NE2	60 THR	478 C	2223	0.869E+00	-0.473E+00	-0.245E-02

1516	GLN	24 NE2	60 THR	478 HG1	2231	0.848E+00	-0.357E+00	-0.915E-04
1517	GLN	24 NE2	60 PHE	486 C	2320	0.797E+00	-0.537E+00	-0.412E-02
1518	GLN	24 NE2	60 ASN	487 C	2340	0.666E+00	-0.848E+00	-0.120E-01
1519	GLN	24 NE2	60 ASN	487 CG	2343	0.398E+00	-0.266E+01	-0.176E+00
1520	GLN	24 NE2	60 ASN	487 OD1	2344	0.432E+00	0.194E+01	-0.146E+00
1521	GLN	24 NE2	60 ASN	4871HD2	2350	0.312E+00	-0.312E+01	-0.326E-01
1522	GLN	24 NE2	60 CYS	488 C	2354	0.102E+01	-0.425E+00	-0.946E-03
1523	GLN	24 HN	61 ASN	487 ND2	2345	0.728E+00	-0.308E+00	-0.229E-03
1524	GLN	241HE2	67 GLN	474 O	2179	0.617E+00	-0.420E+00	-0.453E-03
1525	GLN	241HE2	67 GLN	474 OE1	2183	0.618E+00	-0.465E+00	-0.449E-03
1526	GLN	241HE2	67 GLN	474 NE2	2184	0.836E+00	-0.368E+00	-0.100E-03
1527	GLN	241HE2	67 ALA	475 N	2193	0.454E+00	-0.606E+00	-0.382E-02
1528	GLN	241HE2	67 ALA	475 O	2196	0.240E+00	-0.481E+01	-0.843E-01
1529	GLN	241HE2	67 ALA	475 CB	2197	0.377E+00	-0.559E+00	-0.112E-01
1530	GLN	241HE2	67 GLY	476 O	2206	0.357E+00	-0.143E+01	-0.117E-01
1531	GLN	241HE2	67 SER	477 N	2210	0.426E+00	-0.952E+00	-0.558E-02
1532	GLN	241HE2	67 SER	477 O	2213	0.568E+00	-0.516E+00	-0.744E-03
1533	GLN	241HE2	67 SER	477 OG	2215	0.778E+00	-0.302E+00	-0.131E-03
1534	GLN	241HE2	67 ASN	487 O	2341	0.546E+00	-0.508E+00	-0.939E-03
1535	GLN	241HE2	67 ASN	487 OD1	2344	0.449E+00	-0.814E+00	-0.302E-02
1536	GLN	241HE2	67 ASN	487 ND2	2345	0.273E+00	-0.468E+01	-0.622E-01
1537	GLN	242HE2	68 GLN	474 OE1	2183	0.711E+00	-0.351E+00	-0.194E-03
1538	GLN	242HE2	68 GLN	474 NE2	2184	0.914E+00	-0.318E+00	-0.582E-04
1539	GLN	242HE2	68 ALA	475 N	2193	0.597E+00	-0.322E+00	-0.750E-03
1540	GLN	242HE2	68 ALA	475 O	2196	0.349E+00	-0.166E+01	-0.134E-01
1541	GLN	242HE2	68 GLY	476 N	2203	0.341E+00	-0.119E+01	-0.204E-01
1542	GLN	242HE2	68 GLY	476 CA	2204	0.242E+00	-0.110E+01	-0.631E-01
1543	GLN	242HE2	68 GLY	476 O	2206	0.373E+00	-0.127E+01	-0.901E-02
1544	GLN	242HE2	68 SER	477 N	2210	0.405E+00	-0.108E+01	-0.750E-02
1545	GLN	242HE2	68 SER	477 O	2213	0.488E+00	-0.730E+00	-0.183E-02
1546	GLN	242HE2	68 SER	477 OG	2215	0.713E+00	-0.355E+00	-0.221E-03
1547	GLN	242HE2	68 THR	478 OG1	2226	0.746E+00	-0.307E+00	-0.170E-03
1548	GLN	242HE2	68 ASN	487 O	2341	0.658E+00	-0.341E+00	-0.308E-03
1549	GLN	242HE2	68 ASN	487 OD1	2344	0.437E+00	-0.870E+00	-0.354E-02
1550	GLN	242HE2	68 ASN	487 ND2	2345	0.287E+00	-0.405E+01	-0.499E-01
1551	ALA	25 C	71 ASN	487 ND2	2345	0.981E+00	-0.353E+00	-0.119E-02
1552	ALA	25 C	71 TYR	489 OH	2373	0.708E+00	-0.331E+00	-0.784E-02
1553	ALA	25 O	72 TYR	489 HH	2382	0.626E+00	-0.323E+00	0.000E+00
1554	LYS	26 C	81 LEU	455 O	1844	0.954E+00	-0.334E+00	-0.119E-02
1555	LYS	26 C	81 TYR	489 OH	2373	0.610E+00	-0.568E+00	-0.189E-01
1556	LYS	26 O	82 TYR	489 HH	2382	0.546E+00	-0.442E+00	0.000E+00
1557	THR	27 CA	102 TYR	489 OH	2373	0.392E+00	0.613E+00	-0.213E+00
1558	THR	27 CA	102 TYR	489 HH	2382	0.352E+00	-0.641E+00	0.000E+00
1559	THR	27 C	103 ASN	487 O	2341	0.764E+00	-0.351E+00	-0.453E-02
1560	THR	27 C	103 ASN	487 ND2	2345	0.916E+00	-0.385E+00	-0.179E-02
1561	THR	27 O	104 LEU	455 C	1843	0.848E+00	-0.315E+00	-0.242E-02
1562	THR	27 O	104 PHE	456 CE1	1868	0.421E+00	0.385E+00	-0.131E+00
1563	THR	27 O	104 PHE	456 CZ	1870	0.404E+00	0.273E+00	-0.158E+00
1564	THR	27 O	104 PHE	456 HE1	1877	0.396E+00	-0.358E+00	-0.386E-01
1565	THR	27 O	104 PHE	456 HZ	1879	0.363E+00	-0.416E+00	-0.584E-01
1566	THR	27 O	104 ASN	487 C	2340	0.774E+00	-0.398E+00	-0.418E-02
1567	THR	27 O	104 CYS	488 C	2354	0.835E+00	-0.363E+00	-0.265E-02
1568	THR	27 O	104 TYR	489 C	2364	0.897E+00	-0.312E+00	-0.173E-02
1569	THR	27 O	104 TYR	489 CE1	2370	0.424E+00	0.438E+00	-0.127E+00
1570	THR	27 O	104 TYR	489 CE2	2371	0.442E+00	0.395E+00	-0.104E+00
1571	THR	27 O	104 TYR	489 CZ	2372	0.358E+00	-0.774E+00	-0.218E+00
1572	THR	27 O	104 TYR	489 HE1	2380	0.411E+00	-0.358E+00	-0.316E-01
1573	THR	27 O	104 TYR	489 HH	2382	0.173E+00	-0.103E+02	0.000E+00
1574	THR	27 CB	105 TYR	489 OH	2373	0.350E+00	-0.726E+00	-0.232E+00
1575	THR	27 OG1	106 ARG	457 C	1882	0.105E+01	-0.316E+00	-0.750E-03
1576	THR	27 OG1	106 TYR	473 C	2157	0.885E+00	-0.348E+00	-0.208E-02
1577	THR	27 OG1	106 GLN	474 C	2178	0.761E+00	-0.304E+00	-0.512E-02
1578	THR	27 OG1	106 ALA	475 C	2195	0.660E+00	-0.543E+00	-0.119E-01
1579	THR	27 OG1	106 ALA	475 CB	2197	0.448E+00	0.526E+00	-0.119E+00
1580	THR	27 OG1	106 GLY	476 C	2205	0.928E+00	-0.301E+00	-0.155E-02
1581	THR	27 OG1	106 PHE	486 C	2320	0.892E+00	-0.303E+00	-0.198E-02

1582	THR	27	OG1	106	ASN	487	C	2340	0.662E+00	-0.585E+00	-0.117E-01
1583	THR	27	OG1	106	ASN	487	CG	2343	0.706E+00	-0.488E+00	-0.800E-02
1584	THR	27	OG1	106	CYS	488	C	2354	0.853E+00	-0.382E+00	-0.257E-02
1585	THR	27	OG1	106	TYR	489	CZ	2372	0.471E+00	-0.418E+00	-0.822E-01
1586	THR	27	OG1	106	TYR	489	OH	2373	0.339E+00	0.200E+01	-0.339E+00
1587	THR	27	OG1	106	TYR	489	HH	2382	0.355E+00	-0.138E+01	0.000E+00
1588	THR	27	HG1	111	ALA	475	O	2196	0.716E+00	-0.304E+00	-0.185E-03
1589	THR	27	HG1	111	ASN	487	O	2341	0.666E+00	-0.330E+00	-0.285E-03
1590	THR	27	HG1	111	ASN	487	ND2	2345	0.715E+00	-0.429E+00	-0.255E-03
1591	THR	27	HG1	111	TYR	489	OH	2373	0.427E+00	-0.732E+00	-0.475E-02
1592	PHE	28	N	115	TYR	489	OH	2373	0.374E+00	0.950E+00	-0.285E+00
1593	PHE	28	N	115	TYR	489	HH	2382	0.306E+00	-0.129E+01	0.000E+00
1594	PHE	28	CA	116	TYR	489	OH	2373	0.395E+00	0.661E-01	-0.206E+00
1595	PHE	28	C	117	TYR	489	OH	2373	0.493E+00	-0.696E+00	-0.641E-01
1596	PHE	28	O	118	TYR	489	HH	2382	0.420E+00	-0.750E+00	0.000E+00
1597	PHE	28	HN	126	TYR	489	OH	2373	0.443E+00	-0.385E+00	-0.382E-02
1598	PHE	28	HA	127	TYR	489	OH	2373	0.321E+00	-0.396E+00	-0.904E-01
1599	LEU	29	N	135	TYR	489	HH	2382	0.495E+00	-0.349E+00	0.000E+00
1600	LEU	29	C	137	TYR	489	OH	2373	0.714E+00	-0.327E+00	-0.747E-02
1601	ASP	30	N	154	TYR	489	HH	2382	0.548E+00	-0.434E+00	0.000E+00
1602	ASP	30	C	156	GLN	493	NE2	2444	0.794E+00	-0.437E+00	-0.421E-02
1603	ASP	30	O	157	LEU	455	C	1843	0.793E+00	-0.321E+00	-0.362E-02
1604	ASP	30	O	157	LEU	455	CD2	1848	0.410E+00	0.293E+00	-0.167E+00
1605	ASP	30	O	157	GLN	493	CD	2442	0.707E+00	-0.463E+00	-0.717E-02
1606	ASP	30	CB	158	PHE	456	CE1	1868	0.435E+00	0.309E-01	-0.111E+00
1607	ASP	30	CG	159	LYS	417	NZ	1250	0.578E+00	-0.390E+00	-0.275E-01
1608	ASP	30	CG	159	TYR	453	OH	1807	0.841E+00	-0.317E+00	-0.281E-02
1609	ASP	30	CG	159	LEU	455	O	1844	0.728E+00	-0.544E+00	-0.601E-02
1610	ASP	30	CG	159	TYR	489	OH	2373	0.780E+00	-0.361E+00	-0.441E-02
1611	ASP	30	OD1	160	LYS	417	HZ1	1261	0.648E+00	-0.354E+00	-0.337E-03
1612	ASP	30	OD1	160	LYS	417	HZ2	1262	0.540E+00	-0.524E+00	-0.100E-02
1613	ASP	30	OD1	160	LYS	417	HZ3	1263	0.703E+00	-0.301E+00	-0.207E-03
1614	ASP	30	OD1	160	LEU	455	C	1843	0.894E+00	-0.382E+00	-0.176E-02
1615	ASP	30	OD2	161	LYS	417	HZ1	1261	0.577E+00	-0.453E+00	-0.676E-03
1616	ASP	30	OD2	161	LYS	417	HZ2	1262	0.474E+00	-0.712E+00	-0.218E-02
1617	ASP	30	OD2	161	LYS	417	HZ3	1263	0.626E+00	-0.380E+00	-0.415E-03
1618	ASP	30	OD2	161	LEU	455	C	1843	0.822E+00	-0.439E+00	-0.291E-02
1619	ASP	30	OD2	161	PHE	456	HE1	1877	0.431E+00	-0.380E+00	-0.244E-01
1620	ASP	30	OD2	161	TYR	489	HH	2382	0.691E+00	-0.348E+00	0.000E+00
1621	LYS	31	N	166	PHE	456	CZ	1870	0.416E+00	0.201E+00	-0.152E+00
1622	LYS	31	N	166	PHE	456	HZ	1879	0.318E+00	-0.477E+00	-0.780E-01
1623	LYS	31	N	166	TYR	489	HH	2382	0.471E+00	-0.484E+00	0.000E+00
1624	LYS	31	N	166	GLN	493	CD	2442	0.741E+00	-0.368E+00	-0.633E-02
1625	LYS	31	C	168	LEU	455	O	1844	0.986E+00	-0.318E+00	-0.984E-03
1626	LYS	31	C	168	CYS	488	O	2355	0.103E+01	-0.314E+00	-0.768E-03
1627	LYS	31	C	168	TYR	489	OH	2373	0.704E+00	-0.426E+00	-0.814E-02
1628	LYS	31	C	168	PHE	490	O	2386	0.905E+00	-0.330E+00	-0.164E-02
1629	LYS	31	C	168	LEU	492	O	2420	0.100E+01	-0.311E+00	-0.899E-03
1630	LYS	31	C	168	GLN	493	OE1	2443	0.712E+00	-0.621E+00	-0.685E-02
1631	LYS	31	C	168	GLN	493	NE2	2444	0.666E+00	-0.995E+00	-0.119E-01
1632	LYS	31	C	168	SER	494	O	2456	0.103E+01	-0.308E+00	-0.737E-03
1633	LYS	31	O	169	GLN	493	CD	2442	0.619E+00	-0.680E+00	-0.158E-01
1634	LYS	31	O	169	GLN	4931	HE2	2451	0.578E+00	-0.481E+00	-0.667E-03
1635	LYS	31	O	169	GLN	4932	HE2	2452	0.591E+00	-0.459E+00	-0.586E-03
1636	LYS	31	CB	170	PHE	456	CZ	1870	0.429E+00	0.458E-01	-0.117E+00
1637	LYS	31	CB	170	TYR	489	CE1	2370	0.428E+00	0.839E-01	-0.119E+00
1638	LYS	31	CG	171	TYR	489	CD1	2368	0.441E+00	-0.240E-01	-0.105E+00
1639	LYS	31	CD	172	TYR	489	CB	2366	0.389E+00	0.136E-01	-0.175E+00
1640	LYS	31	CD	172	TYR	489	CG	2367	0.356E+00	-0.375E-01	-0.116E+00
1641	LYS	31	CD	172	TYR	489	CD2	2369	0.433E+00	0.363E-01	-0.113E+00
1642	LYS	31	CD	172	TYR	489	CE1	2370	0.375E+00	0.528E-01	-0.155E+00
1643	LYS	31	CE	173	TYR	489	CB	2366	0.399E+00	0.186E-01	-0.168E+00
1644	LYS	31	CE	173	TYR	489	CG	2367	0.422E+00	-0.349E-01	-0.125E+00
1645	LYS	31	CE	173	TYR	489	CD1	2368	0.407E+00	0.621E-01	-0.141E+00
1646	LYS	31	NZ	174	TYR	489	C	2364	0.576E+00	-0.328E+00	-0.281E-01
1647	LYS	31	NZ	174	TYR	489	CB	2366	0.435E+00	0.534E-01	-0.142E+00

1648	LYS	31 NZ	174	GLN	493 CD	2442	0.405E+00	-0.823E+00	-0.167E+00
1649	LYS	31 NZ	174	GLN	493 OE1	2443	0.341E+00	0.122E+01	-0.301E+00
1650	LYS	31 NZ	174	GLN	493 NE2	2444	0.420E+00	0.986E+00	-0.185E+00
1651	LYS	31 NZ	174	GLN	4932HE2	2452	0.379E+00	-0.595E+00	-0.110E-01
1652	LYS	31 HN	175	TYR	489 OH	2373	0.476E+00	-0.346E+00	-0.249E-02
1653	LYS	31 HZ1	185	GLU	484 OE2	2304	0.678E+00	-0.365E+00	-0.256E-03
1654	LYS	31 HZ1	185	PHE	490 O	2386	0.572E+00	-0.320E+00	-0.710E-03
1655	LYS	31 HZ1	185	LEU	492 O	2420	0.604E+00	-0.313E+00	-0.512E-03
1656	LYS	31 HZ1	185	GLN	493 OE1	2443	0.353E+00	-0.131E+01	-0.124E-01
1657	LYS	31 HZ1	185	GLN	493 NE2	2444	0.417E+00	-0.119E+01	-0.634E-02
1658	LYS	31 HZ2	186	GLU	484 OE2	2304	0.679E+00	-0.364E+00	-0.254E-03
1659	LYS	31 HZ2	186	TYR	489 O	2365	0.554E+00	-0.357E+00	-0.856E-03
1660	LYS	31 HZ2	186	PHE	490 N	2383	0.459E+00	-0.393E+00	-0.362E-02
1661	LYS	31 HZ2	186	PHE	490 O	2386	0.429E+00	-0.634E+00	-0.395E-02
1662	LYS	31 HZ2	186	LEU	492 O	2420	0.563E+00	-0.365E+00	-0.783E-03
1663	LYS	31 HZ2	186	GLN	493 OE1	2443	0.306E+00	-0.196E+01	-0.281E-01
1664	LYS	31 HZ2	186	GLN	493 NE2	2444	0.437E+00	-0.106E+01	-0.482E-02
1665	LYS	31 HZ3	187	PHE	490 O	2386	0.563E+00	-0.332E+00	-0.781E-03
1666	LYS	31 HZ3	187	LEU	492 O	2420	0.603E+00	-0.315E+00	-0.519E-03
1667	LYS	31 HZ3	187	GLN	493 OE1	2443	0.310E+00	-0.188E+01	-0.258E-01
1668	LYS	31 HZ3	187	GLN	493 NE2	2444	0.351E+00	-0.188E+01	-0.172E-01
1669	PHE	32 C	190	GLN	493 NE2	2444	0.854E+00	-0.477E+00	-0.272E-02
1670	ASN	33 C	210	ARG	403 NH1	1048	0.887E+00	-0.392E+00	-0.217E-02
1671	ASN	33 C	210	ARG	403 NH2	1049	0.825E+00	-0.442E+00	-0.335E-02
1672	ASN	33 C	210	TYR	453 OH	1807	0.748E+00	-0.323E+00	-0.567E-02
1673	ASN	33 C	210	GLN	493 OE1	2443	0.974E+00	-0.310E+00	-0.106E-02
1674	ASN	33 C	210	GLN	493 NE2	2444	0.836E+00	-0.557E+00	-0.310E-02
1675	ASN	33 C	210	SER	494 O	2456	0.939E+00	-0.303E+00	-0.131E-02
1676	ASN	33 O	211	ARG	403 CZ	1047	0.830E+00	-0.307E+00	-0.275E-02
1677	ASN	33 O	211	ARG	4032HH2	1062	0.648E+00	-0.337E+00	-0.336E-03
1678	ASN	33 CG	213	ARG	403 NH1	1048	0.100E+01	-0.306E+00	-0.103E-02
1679	HIS	34 N	222	GLN	493 CD	2442	0.754E+00	-0.432E+00	-0.571E-02
1680	HIS	34 N	222	GLN	4931HE2	2451	0.647E+00	-0.355E+00	-0.464E-03
1681	HIS	34 C	224	ARG	403 NH1	1048	0.976E+00	-0.362E+00	-0.122E-02
1682	HIS	34 C	224	ARG	403 NH2	1049	0.835E+00	-0.465E+00	-0.312E-02
1683	HIS	34 C	224	TYR	453 OH	1807	0.694E+00	-0.399E+00	-0.880E-02
1684	HIS	34 C	224	LEU	492 O	2420	0.962E+00	-0.301E+00	-0.113E-02
1685	HIS	34 C	224	GLN	493 O	2439	0.886E+00	-0.347E+00	-0.186E-02
1686	HIS	34 C	224	GLN	493 OE1	2443	0.744E+00	-0.522E+00	-0.527E-02
1687	HIS	34 C	224	GLN	493 NE2	2444	0.560E+00	-0.131E+01	-0.328E-01
1688	HIS	34 C	224	SER	494 N	2453	0.803E+00	-0.392E+00	-0.393E-02
1689	HIS	34 C	224	SER	494 O	2456	0.663E+00	-0.604E+00	-0.105E-01
1690	HIS	34 C	224	SER	494 OG	2458	0.812E+00	-0.454E+00	-0.345E-02
1691	HIS	34 C	224	TYR	495 N	2464	0.873E+00	-0.307E+00	-0.238E-02
1692	HIS	34 C	224	TYR	495 O	2467	0.735E+00	-0.448E+00	-0.569E-02
1693	HIS	34 O	225	GLN	493 CD	2442	0.643E+00	-0.591E+00	-0.126E-01
1694	HIS	34 O	225	GLN	4931HE2	2451	0.463E+00	-0.758E+00	-0.252E-02
1695	HIS	34 O	225	GLN	4932HE2	2452	0.568E+00	-0.469E+00	-0.737E-03
1696	HIS	34 O	225	SER	494 C	2455	0.689E+00	-0.372E+00	-0.838E-02
1697	HIS	34 O	225	SER	494 HG	2463	0.643E+00	-0.394E+00	-0.352E-03
1698	HIS	34 O	225	TYR	495 C	2466	0.717E+00	-0.443E+00	-0.658E-02
1699	HIS	34 CB	226	TYR	453 OH	1807	0.464E+00	0.224E+00	-0.100E+00
1700	HIS	34 CG	227	ARG	403 NH2	1049	0.617E+00	-0.346E+00	-0.187E-01
1701	HIS	34 CG	227	TYR	453 OH	1807	0.379E+00	-0.686E+00	-0.207E+00
1702	HIS	34 CG	227	GLN	493 NE2	2444	0.656E+00	-0.393E+00	-0.130E-01
1703	HIS	34 ND1	228	ARG	403 CZ	1047	0.588E+00	-0.483E+00	-0.249E-01
1704	HIS	34 ND1	228	ARG	4032HH1	1060	0.510E+00	-0.459E+00	-0.191E-02
1705	HIS	34 ND1	228	ARG	4031HH2	1061	0.530E+00	-0.421E+00	-0.153E-02
1706	HIS	34 ND1	228	ARG	4032HH2	1062	0.400E+00	-0.842E+00	-0.814E-02
1707	HIS	34 ND1	228	GLU	406 CD	1088	0.884E+00	-0.301E+00	-0.222E-02
1708	HIS	34 ND1	228	TYR	453 OH	1807	0.390E+00	0.965E+00	-0.248E+00
1709	HIS	34 ND1	228	TYR	453 HH	1816	0.328E+00	-0.121E+01	0.000E+00
1710	HIS	34 ND1	228	GLN	493 CD	2442	0.790E+00	-0.318E+00	-0.434E-02
1711	HIS	34 CD2	229	TYR	453 OH	1807	0.347E+00	0.929E+00	-0.199E+00
1712	HIS	34 CD2	229	TYR	453 HH	1816	0.343E+00	-0.755E+00	0.000E+00
1713	HIS	34 CD2	229	LEU	455 CD1	1847	0.409E+00	0.175E+00	-0.139E+00

HIS	34 CD2	229 LEU	455 CD2	1848	0.391E+00	0.197E+00	-0.154E+00
HIS	34 CE1	230 TYR	453 OH	1807	0.369E+00	-0.686E-01	-0.216E+00
HIS	34 NE2	231 TYR	453 OH	1807	0.341E+00	0.321E+00	-0.287E+00
HIS	34 NE2	231 LEU	455 CD1	1847	0.429E+00	0.512E-01	-0.150E+00
HIS	34 HN	232 GLN	493 NE2	2444	0.672E+00	-0.380E+00	-0.368E-03
HIS	34 HD1	236 ARG	403 NH2	1049	0.449E+00	-0.414E+00	-0.408E-02
HIS	34 HD2	237 TYR	453 OH	1807	0.377E+00	-0.400E+00	-0.500E-01
HIS	34 HE1	238 ARG	403 NH1	1048	0.427E+00	-0.374E+00	-0.258E-01
HIS	34 HE1	238 ARG	403 NH2	1049	0.406E+00	-0.426E+00	-0.337E-01
GLU	35 N	239 GLN	493 CD	2442	0.655E+00	-0.454E+00	-0.132E-01
GLU	35 N	239 GLN	4931HE2	2451	0.515E+00	-0.468E+00	-0.180E-02
GLU	35 N	239 GLN	4932HE2	2452	0.573E+00	-0.369E+00	-0.962E-03
GLU	35 C	241 GLN	493 NE2	2444	0.727E+00	-0.544E+00	-0.711E-02
GLU	35 O	242 GLN	493 CD	2442	0.895E+00	-0.361E+00	-0.175E-02
GLU	35 O	242 GLN	4931HE2	2451	0.729E+00	-0.315E+00	-0.166E-03
GLU	35 O	242 GLN	4932HE2	2452	0.748E+00	-0.301E+00	-0.142E-03
GLU	35 CG	244 GLN	493 NE2	2444	0.434E+00	0.124E+00	-0.143E+00
GLU	35 CD	245 PHE	490 O	2386	0.937E+00	-0.329E+00	-0.133E-02
GLU	35 CD	245 LEU	492 O	2420	0.765E+00	-0.509E+00	-0.447E-02
GLU	35 CD	245 GLN	493 N	2436	0.815E+00	-0.316E+00	-0.361E-02
GLU	35 CD	245 GLN	493 O	2439	0.876E+00	-0.408E+00	-0.199E-02
GLU	35 CD	245 GLN	493 OE1	2443	0.539E+00	-0.117E+01	-0.352E-01
GLU	35 CD	245 GLN	493 NE2	2444	0.377E+00	-0.402E+01	-0.196E+00
GLU	35 CD	245 SER	494 N	2453	0.719E+00	-0.554E+00	-0.759E-02
GLU	35 CD	245 SER	494 O	2456	0.719E+00	-0.596E+00	-0.650E-02
GLU	35 CD	245 SER	494 OG	2458	0.707E+00	-0.677E+00	-0.790E-02
GLU	35 CD	245 TYR	495 N	2464	0.929E+00	-0.321E+00	-0.165E-02
GLU	35 CD	245 TYR	495 O	2467	0.886E+00	-0.374E+00	-0.186E-02
GLU	35 OE1	246 TYR	449 HH	1741	0.717E+00	-0.366E+00	0.000E+00
GLU	35 OE1	246 LEU	492 C	2419	0.926E+00	-0.408E+00	-0.143E-02
GLU	35 OE1	246 GLN	493 C	2438	0.834E+00	-0.354E+00	-0.267E-02
GLU	35 OE1	246 GLN	493 CD	2442	0.600E+00	-0.106E+01	-0.190E-01
GLU	35 OE1	246 GLN	4931HE2	2451	0.475E+00	-0.111E+01	-0.216E-02
GLU	35 OE1	246 GLN	4932HE2	2452	0.410E+00	-0.160E+01	-0.519E-02
GLU	35 OE1	246 SER	494 C	2455	0.845E+00	-0.399E+00	-0.246E-02
GLU	35 OE1	246 SER	494 HN	2459	0.688E+00	-0.415E+00	-0.235E-03
GLU	35 OE1	246 SER	494 HG	2463	0.625E+00	-0.650E+00	-0.416E-03
GLU	35 OE1	246 TYR	495 C	2466	0.959E+00	-0.419E+00	-0.116E-02
GLU	35 OE2	247 TYR	449 HH	1741	0.785E+00	-0.310E+00	0.000E+00
GLU	35 OE2	247 TYR	489 C	2364	0.974E+00	-0.410E+00	-0.106E-02
GLU	35 OE2	247 PHE	490 C	2385	0.910E+00	-0.401E+00	-0.159E-02
GLU	35 OE2	247 LEU	492 C	2419	0.715E+00	-0.640E+00	-0.672E-02
GLU	35 OE2	247 GLN	493 C	2438	0.647E+00	-0.568E+00	-0.121E-01
GLU	35 OE2	247 GLN	493 CD	2442	0.381E+00	-0.318E+01	-0.195E+00
GLU	35 OE2	247 GLN	493 OE1	2443	0.433E+00	0.214E+01	-0.124E+00
GLU	35 OE2	247 GLN	493 HA	2446	0.513E+00	-0.343E+00	-0.798E-02
GLU	35 OE2	247 GLN	4931HE2	2451	0.268E+00	-0.519E+01	-0.551E-01
GLU	35 OE2	247 SER	494 C	2455	0.716E+00	-0.538E+00	-0.667E-02
GLU	35 OE2	247 SER	494 HN	2459	0.525E+00	-0.738E+00	-0.118E-02
GLU	35 OE2	247 SER	494 HG	2463	0.527E+00	-0.948E+00	-0.116E-02
GLU	35 OE2	247 TYR	495 C	2466	0.887E+00	-0.474E+00	-0.185E-02
GLU	35 HN	248 GLN	493 NE2	2444	0.595E+00	-0.533E+00	-0.768E-03
ALA	36 C	256 GLN	493 NE2	2444	0.101E+01	-0.383E+00	-0.101E-02
GLU	37 C	266 GLN	498 NE2	2520	0.850E+00	-0.412E+00	-0.280E-02
GLU	37 C	266 ASN	501 ND2	2564	0.923E+00	-0.319E+00	-0.171E-02
GLU	37 O	267 TYR	495 C	2466	0.920E+00	-0.322E+00	-0.148E-02
GLU	37 O	267 GLY	496 C	2487	0.919E+00	-0.301E+00	-0.149E-02
GLU	37 O	267 GLN	498 CD	2518	0.862E+00	-0.384E+00	-0.220E-02
GLU	37 O	267 GLN	4981HE2	2527	0.746E+00	-0.302E+00	-0.144E-03
GLU	37 O	267 ASN	501 CG	2562	0.899E+00	-0.314E+00	-0.171E-02
GLU	37 CD	270 ARG	403 NH1	1048	0.676E+00	-0.792E+00	-0.110E-01
GLU	37 CD	270 ARG	403 NH2	1049	0.645E+00	-0.870E+00	-0.144E-01
GLU	37 CD	270 ASP	405 OD1	1076	0.101E+01	-0.423E+00	-0.850E-03
GLU	37 CD	270 ASP	405 OD2	1077	0.954E+00	-0.460E+00	-0.119E-02
GLU	37 CD	270 SER	494 O	2456	0.105E+01	-0.320E+00	-0.690E-03
GLU	37 CD	270 TYR	495 O	2467	0.915E+00	-0.355E+00	-0.154E-02

1780	GLU	37 CD	270 ASN	501 ND2	2564	0.101E+01	-0.451E+00	-0.981E-03
1781	GLU	37 CD	270 TYR	505 CE2	2610	0.532E+00	-0.351E+00	-0.356E-01
1782	GLU	37 CD	270 TYR	505 OH	2612	0.452E+00	-0.120E+01	-0.101E+00
1783	GLU	37 OE1	271 ARG	403 NE	1046	0.789E+00	-0.433E+00	-0.456E-02
1784	GLU	37 OE1	271 ARG	403 CZ	1047	0.694E+00	-0.669E+00	-0.801E-02
1785	GLU	37 OE1	271 ARG	4031HH1	1059	0.734E+00	-0.415E+00	-0.159E-03
1786	GLU	37 OE1	271 ARG	4032HH1	1060	0.621E+00	-0.578E+00	-0.433E-03
1787	GLU	37 OE1	271 ARG	4031HH2	1061	0.692E+00	-0.464E+00	-0.227E-03
1788	GLU	37 OE1	271 ARG	4032HH2	1062	0.593E+00	-0.637E+00	-0.571E-03
1789	GLU	37 OE1	271 ASP	405 CG	1075	0.984E+00	-0.483E+00	-0.990E-03
1790	GLU	37 OE1	271 TYR	495 C	2466	0.103E+01	-0.376E+00	-0.742E-03
1791	GLU	37 OE1	271 ASN	501 CG	2562	0.972E+00	-0.386E+00	-0.107E-02
1792	GLU	37 OE1	271 GLY	504 C	2596	0.102E+01	-0.360E+00	-0.823E-03
1793	GLU	37 OE1	271 TYR	505 C	2603	0.103E+01	-0.378E+00	-0.760E-03
1794	GLU	37 OE1	271 TYR	505 CZ	2611	0.459E+00	-0.609E+00	-0.862E-01
1795	GLU	37 OE1	271 TYR	505 OH	2612	0.380E+00	0.202E+01	-0.255E+00
1796	GLU	37 OE1	271 TYR	505 HE2	2620	0.384E+00	-0.637E+00	-0.447E-01
1797	GLU	37 OE1	271 TYR	505 HH	2621	0.447E+00	-0.104E+01	0.000E+00
1798	GLU	37 OE2	272 ARG	403 NE	1046	0.754E+00	-0.469E+00	-0.594E-02
1799	GLU	37 OE2	272 ARG	403 CZ	1047	0.637E+00	-0.795E+00	-0.133E-01
1800	GLU	37 OE2	272 ARG	4031HH1	1059	0.663E+00	-0.506E+00	-0.294E-03
1801	GLU	37 OE2	272 ARG	4032HH1	1060	0.518E+00	-0.862E+00	-0.128E-02
1802	GLU	37 OE2	272 ARG	4031HH2	1061	0.648E+00	-0.530E+00	-0.337E-03
1803	GLU	37 OE2	272 ARG	4032HH2	1062	0.508E+00	-0.905E+00	-0.145E-02
1804	GLU	37 OE2	272 ASP	405 CG	1075	0.981E+00	-0.485E+00	-0.101E-02
1805	GLU	37 OE2	272 TYR	453 HH	1816	0.771E+00	-0.320E+00	0.000E+00
1806	GLU	37 OE2	272 TYR	495 C	2466	0.104E+01	-0.372E+00	-0.711E-03
1807	GLU	37 OE2	272 TYR	505 CZ	2611	0.544E+00	-0.407E+00	-0.334E-01
1808	GLU	37 OE2	272 TYR	505 OH	2612	0.440E+00	0.138E+01	-0.127E+00
1809	GLU	37 OE2	272 TYR	505 HE2	2620	0.475E+00	-0.373E+00	-0.141E-01
1810	GLU	37 OE2	272 TYR	505 HH	2621	0.464E+00	-0.951E+00	0.000E+00
1811	ASP	38 N	279 TYR	495 C	2466	0.804E+00	-0.379E+00	-0.389E-02
1812	ASP	38 N	279 GLY	496 C	2487	0.865E+00	-0.313E+00	-0.253E-02
1813	ASP	38 N	279 GLN	498 CD	2518	0.904E+00	-0.335E+00	-0.194E-02
1814	ASP	38 C	281 TYR	449 OH	1732	0.623E+00	-0.333E+00	-0.168E-01
1815	ASP	38 C	281 GLN	498 NE2	2520	0.673E+00	-0.595E+00	-0.112E-01
1816	ASP	38 O	282 TYR	449 HH	1741	0.564E+00	-0.365E+00	0.000E+00
1817	ASP	38 O	282 GLN	498 CD	2518	0.695E+00	-0.478E+00	-0.793E-02
1818	ASP	38 O	282 GLN	4981HE2	2527	0.551E+00	-0.475E+00	-0.885E-03
1819	ASP	38 O	282 GLN	4982HE2	2528	0.551E+00	-0.475E+00	-0.886E-03
1820	ASP	38 CG	284 ARG	403 NH2	1049	0.100E+01	-0.392E+00	-0.104E-02
1821	ASP	38 CG	284 GLY	446 O	1696	0.789E+00	-0.428E+00	-0.373E-02
1822	ASP	38 CG	284 GLY	447 O	1703	0.765E+00	-0.452E+00	-0.446E-02
1823	ASP	38 CG	284 ASN	448 N	1707	0.865E+00	-0.309E+00	-0.253E-02
1824	ASP	38 CG	284 ASN	448 O	1710	0.730E+00	-0.509E+00	-0.594E-02
1825	ASP	38 CG	284 TYR	449 N	1721	0.933E+00	-0.310E+00	-0.160E-02
1826	ASP	38 CG	284 TYR	449 CE1	1729	0.479E+00	-0.438E+00	-0.624E-01
1827	ASP	38 CG	284 TYR	449 OH	1732	0.368E+00	-0.199E+01	-0.217E+00
1828	ASP	38 CG	284 GLN	493 O	2439	0.103E+01	-0.311E+00	-0.769E-03
1829	ASP	38 CG	284 GLN	493 NE2	2444	0.909E+00	-0.585E+00	-0.188E-02
1830	ASP	38 CG	284 SER	494 N	2453	0.869E+00	-0.386E+00	-0.246E-02
1831	ASP	38 CG	284 SER	494 O	2456	0.677E+00	-0.651E+00	-0.927E-02
1832	ASP	38 CG	284 SER	494 OG	2458	0.644E+00	-0.794E+00	-0.138E-01
1833	ASP	38 CG	284 TYR	495 N	2464	0.765E+00	-0.434E+00	-0.525E-02
1834	ASP	38 CG	284 TYR	495 O	2467	0.496E+00	-0.117E+01	-0.566E-01
1835	ASP	38 CG	284 GLY	496 N	2485	0.605E+00	-0.528E+00	-0.210E-01
1836	ASP	38 CG	284 GLY	496 O	2488	0.592E+00	-0.752E+00	-0.204E-01
1837	ASP	38 CG	284 PHE	497 N	2492	0.699E+00	-0.391E+00	-0.899E-02
1838	ASP	38 CG	284 PHE	497 O	2495	0.853E+00	-0.373E+00	-0.233E-02
1839	ASP	38 CG	284 GLN	498 O	2515	0.104E+01	-0.307E+00	-0.723E-03
1840	ASP	38 CG	284 GLN	498 OE1	2519	0.759E+00	-0.566E+00	-0.468E-02
1841	ASP	38 CG	284 GLN	498 NE2	2520	0.542E+00	-0.159E+01	-0.394E-01
1842	ASP	38 CG	284 ASN	501 OD1	2563	0.886E+00	-0.364E+00	-0.186E-02
1843	ASP	38 CG	284 ASN	501 ND2	2564	0.822E+00	-0.612E+00	-0.342E-02
1844	ASP	38 OD1	285 GLY	446 C	1695	0.804E+00	-0.463E+00	-0.332E-02
1845	ASP	38 OD1	285 GLY	447 C	1702	0.755E+00	-0.519E+00	-0.485E-02

1846	ASP	38 OD1	285	ASN	448 C	1709	0.856E+00	-0.442E+00	-0.228E-02
1847	ASP	38 OD1	285	TYR	449 CZ	1731	0.449E+00	-0.568E+00	-0.959E-01
1848	ASP	38 OD1	285	TYR	449 OH	1732	0.324E+00	0.277E+01	-0.310E+00
1849	ASP	38 OD1	285	TYR	449 HE1	1739	0.444E+00	-0.389E+00	-0.206E-01
1850	ASP	38 OD1	285	TYR	449 HH	1741	0.282E+00	-0.321E+01	0.000E+00
1851	ASP	38 OD1	285	SER	494 C	2455	0.831E+00	-0.364E+00	-0.274E-02
1852	ASP	38 OD1	285	SER	494 HG	2463	0.709E+00	-0.448E+00	-0.197E-03
1853	ASP	38 OD1	285	TYR	495 C	2466	0.683E+00	-0.672E+00	-0.880E-02
1854	ASP	38 OD1	285	GLY	496 C	2487	0.658E+00	-0.675E+00	-0.110E-01
1855	ASP	38 OD1	285	PHE	497 C	2494	0.841E+00	-0.404E+00	-0.254E-02
1856	ASP	38 OD1	285	GLN	498 CD	2518	0.612E+00	-0.903E+00	-0.168E-01
1857	ASP	38 OD1	285	GLN	4981HE2	2527	0.419E+00	-0.134E+01	-0.457E-02
1858	ASP	38 OD1	285	GLN	4982HE2	2528	0.482E+00	-0.949E+00	-0.199E-02
1859	ASP	38 OD1	285	ASN	501 CG	2562	0.940E+00	-0.360E+00	-0.131E-02
1860	ASP	38 OD2	286	GLY	446 C	1695	0.846E+00	-0.424E+00	-0.245E-02
1861	ASP	38 OD2	286	GLY	447 C	1702	0.713E+00	-0.577E+00	-0.680E-02
1862	ASP	38 OD2	286	ASN	448 C	1709	0.755E+00	-0.550E+00	-0.483E-02
1863	ASP	38 OD2	286	ASN	448 CG	1712	0.100E+01	-0.328E+00	-0.900E-03
1864	ASP	38 OD2	286	TYR	449 C	1723	0.936E+00	-0.386E+00	-0.134E-02
1865	ASP	38 OD2	286	TYR	449 CE1	1729	0.406E+00	0.647E+00	-0.154E+00
1866	ASP	38 OD2	286	TYR	449 CZ	1731	0.426E+00	-0.650E+00	-0.125E+00
1867	ASP	38 OD2	286	TYR	449 OH	1732	0.349E+00	0.225E+01	-0.332E+00
1868	ASP	38 OD2	286	TYR	449 HE1	1739	0.326E+00	-0.881E+00	-0.860E-01
1869	ASP	38 OD2	286	TYR	449 HH	1741	0.317E+00	-0.229E+01	0.000E+00
1870	ASP	38 OD2	286	GLN	493 CD	2442	0.102E+01	-0.365E+00	-0.807E-03
1871	ASP	38 OD2	286	GLN	4931HE2	2451	0.821E+00	-0.314E+00	-0.814E-04
1872	ASP	38 OD2	286	SER	494 C	2455	0.652E+00	-0.573E+00	-0.116E-01
1873	ASP	38 OD2	286	SER	494 HG	2463	0.594E+00	-0.642E+00	-0.567E-03
1874	ASP	38 OD2	286	TYR	495 C	2466	0.472E+00	-0.152E+01	-0.737E-01
1875	ASP	38 OD2	286	TYR	495 O	2467	0.385E+00	0.217E+01	-0.225E+00
1876	ASP	38 OD2	286	GLY	496 CA	2486	0.406E+00	0.459E+00	-0.174E+00
1877	ASP	38 OD2	286	GLY	496 C	2487	0.477E+00	-0.138E+01	-0.696E-01
1878	ASP	38 OD2	286	GLY	496 HN	2489	0.574E+00	-0.394E+00	-0.694E-03
1879	ASP	38 OD2	286	GLY	496 HA2	2491	0.307E+00	-0.679E+00	-0.930E-01
1880	ASP	38 OD2	286	PHE	497 C	2494	0.720E+00	-0.534E+00	-0.641E-02
1881	ASP	38 OD2	286	PHE	497 HN	2503	0.611E+00	-0.318E+00	-0.480E-03
1882	ASP	38 OD2	286	GLN	498 CD	2518	0.623E+00	-0.870E+00	-0.152E-01
1883	ASP	38 OD2	286	GLN	4981HE2	2527	0.431E+00	-0.125E+01	-0.385E-02
1884	ASP	38 OD2	286	GLN	4982HE2	2528	0.558E+00	-0.673E+00	-0.821E-03
1885	ASP	38 OD2	286	ASN	501 CG	2562	0.833E+00	-0.438E+00	-0.269E-02
1886	ASP	38 OD2	286	ASN	5011HD2	2569	0.775E+00	-0.302E+00	-0.115E-03
1887	ASP	38 OD2	286	ASN	5012HD2	2570	0.653E+00	-0.419E+00	-0.320E-03
1888	LEU	39 C	293	GLN	498 NE2	2520	0.864E+00	-0.489E+00	-0.254E-02
1889	PHE	40 C	312	GLN	498 NE2	2520	0.856E+00	-0.475E+00	-0.268E-02
1890	TYR	41 N	330	GLN	498 CD	2518	0.815E+00	-0.347E+00	-0.359E-02
1891	TYR	41 C	332	GLY	446 O	1696	0.872E+00	-0.301E+00	-0.205E-02
1892	TYR	41 C	332	GLN	498 OE1	2519	0.675E+00	-0.592E+00	-0.945E-02
1893	TYR	41 C	332	GLN	498 NE2	2520	0.611E+00	-0.102E+01	-0.198E-01
1894	TYR	41 C	332	THR	500 OG1	2548	0.854E+00	-0.369E+00	-0.256E-02
1895	TYR	41 C	332	ASN	501 OD1	2563	0.854E+00	-0.323E+00	-0.232E-02
1896	TYR	41 C	332	ASN	501 ND2	2564	0.950E+00	-0.404E+00	-0.144E-02
1897	TYR	41 O	333	GLN	498 CD	2518	0.712E+00	-0.480E+00	-0.685E-02
1898	TYR	41 O	333	GLN	4982HE2	2528	0.615E+00	-0.394E+00	-0.460E-03
1899	TYR	41 CD2	337	GLN	498 CD	2518	0.403E+00	-0.609E+00	-0.129E+00
1900	TYR	41 CD2	337	GLN	498 OE1	2519	0.425E+00	0.500E+00	-0.126E+00
1901	TYR	41 CD2	337	GLN	498 NE2	2520	0.389E+00	0.883E+00	-0.187E+00
1902	TYR	41 CD2	337	GLN	4981HE2	2527	0.417E+00	-0.341E+00	-0.552E-02
1903	TYR	41 CD2	337	GLN	4982HE2	2528	0.392E+00	-0.400E+00	-0.790E-02
1904	TYR	41 CE2	339	GLN	498 CB	2516	0.438E+00	0.234E-01	-0.107E+00
1905	TYR	41 CE2	339	GLN	498 CG	2517	0.421E+00	0.162E-01	-0.126E+00
1906	TYR	41 CE2	339	GLN	498 CD	2518	0.377E+00	-0.722E+00	-0.139E+00
1907	TYR	41 CE2	339	GLN	498 OE1	2519	0.376E+00	0.684E+00	-0.202E+00
1908	TYR	41 CE2	339	GLN	498 NE2	2520	0.409E+00	0.771E+00	-0.161E+00
1909	TYR	41 CE2	339	GLN	4982HE2	2528	0.427E+00	-0.318E+00	-0.478E-02
1910	TYR	41 CE2	339	THR	500 OG1	2548	0.442E+00	0.430E+00	-0.113E+00
1911	TYR	41 CE2	339	THR	500 HG1	2553	0.432E+00	-0.308E+00	-0.450E-02

TYR	41 CE2	339 ASN	501 OD1	2563	0.411E+00	0.453E+00	-0.146E+00
TYR	41 CZ	340 GLN	498 OE1	2519	0.488E+00	-0.400E+00	-0.619E-01
TYR	41 CZ	340 GLN	498 NE2	2520	0.540E+00	-0.443E+00	-0.404E-01
TYR	41 CZ	340 THR	500 OG1	2548	0.397E+00	-0.643E+00	-0.181E+00
TYR	41 CZ	340 ASN	501 OD1	2563	0.380E+00	-0.629E+00	-0.196E+00
TYR	41 CZ	340 ASN	501 ND2	2564	0.543E+00	-0.388E+00	-0.393E-01
TYR	41 OH	341 GLN	498 C	2514	0.588E+00	-0.356E+00	-0.235E-01
TYR	41 OH	341 GLN	498 CD	2518	0.532E+00	-0.710E+00	-0.420E-01
TYR	41 OH	341 THR	500 CA	2544	0.449E+00	0.432E+00	-0.118E+00
TYR	41 OH	341 THR	500 C	2545	0.399E+00	-0.120E+01	-0.177E+00
TYR	41 OH	341 THR	500 O	2546	0.399E+00	0.119E+01	-0.207E+00
TYR	41 OH	341 THR	500 CB	2547	0.394E+00	-0.530E+00	-0.209E+00
TYR	41 OH	341 THR	500 HN	2550	0.440E+00	-0.427E+00	-0.397E-02
TYR	41 OH	341 THR	500 HG1	2553	0.305E+00	-0.181E+01	-0.325E-01
TYR	41 OH	341 ASN	501 N	2557	0.422E+00	0.800E+00	-0.177E+00
TYR	41 OH	341 ASN	501 CA	2558	0.445E+00	-0.733E-01	-0.124E+00
TYR	41 OH	341 ASN	501 C	2559	0.573E+00	-0.554E+00	-0.272E-01
TYR	41 OH	341 ASN	501 CG	2562	0.438E+00	-0.991E+00	-0.119E+00
TYR	41 OH	341 ASN	501 OD1	2563	0.333E+00	0.185E+01	-0.337E+00
TYR	41 OH	341 ASN	501 HN	2565	0.466E+00	-0.371E+00	-0.283E-02
TYR	41 OH	341 ASN	5012HD2	2570	0.534E+00	-0.374E+00	-0.126E-02
TYR	41 HN	342 GLN	498 NE2	2520	0.724E+00	-0.308E+00	-0.237E-03
TYR	41 HD2	347 GLN	498 OE1	2519	0.384E+00	-0.473E+00	-0.451E-01
TYR	41 HD2	347 GLN	498 NE2	2520	0.312E+00	-0.117E+01	-0.718E-01
TYR	41 HE2	349 GLN	498 OE1	2519	0.283E+00	-0.113E+01	-0.349E-01
TYR	41 HE2	349 GLN	498 NE2	2520	0.354E+00	-0.850E+00	-0.710E-01
TYR	41 HE2	349 THR	500 OG1	2548	0.399E+00	-0.420E+00	-0.405E-01
TYR	41 HE2	349 ASN	501 OD1	2563	0.398E+00	-0.371E+00	-0.372E-01
TYR	41 HH	350 GLN	498 O	2515	0.644E+00	-0.310E+00	0.000E+00
TYR	41 HH	350 GLN	498 OE1	2519	0.568E+00	-0.451E+00	0.000E+00
TYR	41 HH	350 GLN	498 NE2	2520	0.679E+00	-0.436E+00	0.000E+00
TYR	41 HH	350 THR	500 CA	2544	0.457E+00	-0.323E+00	0.000E+00
TYR	41 HH	350 THR	500 O	2546	0.389E+00	-0.998E+00	0.000E+00
TYR	41 HH	350 THR	500 OG1	2548	0.273E+00	-0.291E+01	0.000E+00
TYR	41 HH	350 ASN	501 N	2557	0.464E+00	-0.496E+00	0.000E+00
TYR	41 HH	350 ASN	501 OD1	2563	0.420E+00	-0.777E+00	0.000E+00
TYR	41 HH	350 ASN	501 ND2	2564	0.624E+00	-0.459E+00	0.000E+00
GLN	42 N	351 GLN	498 CD	2518	0.626E+00	-0.456E+00	-0.172E-01
GLN	42 N	351 GLN	4981HE2	2527	0.574E+00	-0.336E+00	-0.951E-03
GLN	42 N	351 GLN	4982HE2	2528	0.483E+00	-0.500E+00	-0.267E-02
GLN	42 C	353 GLN	498 OE1	2519	0.750E+00	-0.325E+00	-0.502E-02
GLN	42 C	353 GLN	498 NE2	2520	0.695E+00	-0.528E+00	-0.927E-02
GLN	42 O	354 GLN	498 CD	2518	0.808E+00	-0.408E+00	-0.322E-02
GLN	42 O	354 GLN	4982HE2	2528	0.653E+00	-0.373E+00	-0.321E-03
GLN	42 CD	357 GLY	446 O	1696	0.598E+00	-0.661E+00	-0.193E-01
GLN	42 CD	357 GLY	447 O	1703	0.805E+00	-0.370E+00	-0.330E-02
GLN	42 CD	357 ASN	448 O	1710	0.927E+00	-0.302E+00	-0.142E-02
GLN	42 CD	357 TYR	449 OH	1732	0.497E+00	-0.832E+00	-0.614E-01
GLN	42 CD	357 SER	494 OG	2458	0.101E+01	-0.324E+00	-0.944E-03
GLN	42 CD	357 GLN	498 OE1	2519	0.673E+00	-0.639E+00	-0.960E-02
GLN	42 CD	357 GLN	498 NE2	2520	0.513E+00	-0.162E+01	-0.540E-01
GLN	42 OE1	358 LYS	444 C	1657	0.103E+01	-0.336E+00	-0.761E-03
GLN	42 OE1	358 GLY	446 C	1695	0.587E+00	-0.738E+00	-0.215E-01
GLN	42 OE1	358 GLY	447 C	1702	0.714E+00	-0.495E+00	-0.675E-02
GLN	42 OE1	358 ASN	448 C	1709	0.923E+00	-0.337E+00	-0.146E-02
GLN	42 OE1	358 TYR	449 OH	1732	0.440E+00	0.105E+01	-0.126E+00
GLN	42 OE1	358 TYR	449 HH	1741	0.465E+00	-0.720E+00	0.000E+00
GLN	42 OE1	358 TYR	495 C	2466	0.993E+00	-0.303E+00	-0.939E-03
GLN	42 OE1	358 GLY	496 C	2487	0.872E+00	-0.347E+00	-0.204E-02
GLN	42 OE1	358 PHE	497 C	2494	0.919E+00	-0.301E+00	-0.149E-02
GLN	42 OE1	358 GLN	498 CD	2518	0.538E+00	-0.103E+01	-0.356E-01
GLN	42 OE1	358 GLN	498 NE2	2520	0.418E+00	0.252E+01	-0.172E+00
GLN	42 OE1	358 GLN	4981HE2	2527	0.424E+00	-0.112E+01	-0.424E-02
GLN	42 OE1	358 GLN	4982HE2	2528	0.340E+00	-0.202E+01	-0.155E-01
GLN	42 NE2	359 GLY	446 C	1695	0.788E+00	-0.580E+00	-0.439E-02
GLN	42 NE2	359 GLY	447 C	1702	0.871E+00	-0.489E+00	-0.242E-02

GLN	42 NE2	359 ASN	448 C	1709	0.104E+01	-0.395E+00	-0.832E-03
GLN	42 NE2	359 TYR	449 CZ	1731	0.611E+00	-0.338E+00	-0.199E-01
GLN	42 NE2	359 TYR	449 HH	1741	0.469E+00	-0.991E+00	0.000E+00
GLN	42 NE2	359 SER	494 HG	2463	0.965E+00	-0.319E+00	-0.422E-04
GLN	42 NE2	359 TYR	495 C	2466	0.105E+01	-0.395E+00	-0.801E-03
GLN	42 NE2	359 GLY	496 C	2487	0.991E+00	-0.399E+00	-0.112E-02
GLN	42 NE2	359 GLN	498 CD	2518	0.746E+00	-0.737E+00	-0.611E-02
GLN	42 NE2	359 GLN	4981HE2	2527	0.605E+00	-0.683E+00	-0.692E-03
GLN	42 NE2	359 GLN	4982HE2	2528	0.549E+00	-0.845E+00	-0.124E-02
GLN	42 HN	360 GLN	498 OE1	2519	0.653E+00	-0.306E+00	-0.322E-03
GLN	42 HN	360 GLN	498 NE2	2520	0.530E+00	-0.676E+00	-0.153E-02
GLN	42 HA	361 GLN	498 NE2	2520	0.527E+00	-0.345E+00	-0.834E-02
GLN	421HE2	366 TYR	449 OH	1732	0.565E+00	-0.377E+00	-0.890E-03
GLN	421HE2	366 GLN	498 NE2	2520	0.699E+00	-0.509E+00	-0.291E-03
GLN	422HE2	367 GLY	446 O	1696	0.670E+00	-0.319E+00	-0.276E-03
GLN	422HE2	367 TYR	449 OH	1732	0.427E+00	-0.739E+00	-0.478E-02
GLN	422HE2	367 GLN	498 NE2	2520	0.633E+00	-0.622E+00	-0.529E-03
SER	43 N	368 GLN	498 CD	2518	0.866E+00	-0.348E+00	-0.251E-02
SER	43 N	368 GLN	4982HE2	2528	0.704E+00	-0.308E+00	-0.279E-03
SER	43 C	370 GLN	498 NE2	2520	0.979E+00	-0.338E+00	-0.120E-02
SER	43 HN	374 GLN	498 NE2	2520	0.769E+00	-0.360E+00	-0.165E-03
SER	44 C	381 GLN	498 NE2	2520	0.947E+00	-0.356E+00	-0.147E-02
SER	44 OG	384 GLN	498 CD	2518	0.946E+00	-0.357E+00	-0.139E-02
SER	44 HN	385 GLN	498 NE2	2520	0.854E+00	-0.300E+00	-0.880E-04
SER	44 HG	389 GLN	498 NE2	2520	0.852E+00	-0.390E+00	-0.891E-04
LEU	45 C	392 GLN	498 OE1	2519	0.796E+00	-0.400E+00	-0.355E-02
LEU	45 C	392 GLN	498 NE2	2520	0.876E+00	-0.478E+00	-0.234E-02
LEU	45 C	392 THR	500 OG1	2548	0.857E+00	-0.338E+00	-0.251E-02
LEU	45 O	393 GLN	498 CD	2518	0.936E+00	-0.317E+00	-0.134E-02
LEU	45 CG	395 GLN	498 OE1	2519	0.529E+00	-0.308E+00	-0.442E-01
LEU	45 CD2	397 GLN	498 OE1	2519	0.390E+00	0.419E+00	-0.204E+00
